# Single-cell profiling of low-stage endometrial cancers identifies low epithelial vimentin expression as a marker of recurrent disease

**DOI:** 10.1016/j.ebiom.2023.104595

**Published:** 2023-05-03

**Authors:** Hilde E. Lien, Hege F. Berg, Mari K. Halle, Jone Trovik, Ingfrid S. Haldorsen, Lars A. Akslen, Camilla Krakstad

**Affiliations:** a Centre for Cancer Biomarkers, Department of Clinical Science, University of Bergen, Bergen, Norway; b Department of Gynecology and Obstetrics, Haukeland University Hospital, Bergen, Norway; c Mohn Medical Imaging and Visualization Centre, Department of Radiology, Haukeland University Hospital, Bergen, Norway; d Section for Radiology, Department of Clinical Medicine, University of Bergen, Bergen, Norway; e Department of Pathology, Haukeland University Hospital, Bergen, Norway

**Keywords:** Endometrial cancer, Recurrence, FIGO I, Imaging mass cytometry, Tumor heterogeneity, Vimentin, Prognosis

## Abstract

**Background:**

Identification of aggressive low-stage endometrial cancers is challenging. So far, studies have failed to pinpoint robust features or biomarkers associated with risk of recurrence for these patients.

**Methods:**

Imaging mass cytometry was used to examine single-cell expression of 23 proteins in 36 primary FIGO IB endometrial cancers, of which 17 recurred. Single-cell information was extracted for each tumor and unsupervised clustering was used to identify cellular phenotypes. Distinct phenotypes and cellular neighborhoods were compared in relation to recurrence. Cellular differences were validated in a separate gene expression dataset and the TCGA EC dataset. Vimentin protein expression was evaluated by IHC in pre-operative samples from 518 patients to validate its robustness as a prognostic marker.

**Findings:**

The abundance of epithelial, immune or stromal cell types did not associate with recurrence. Clustering of patients based on tumor single cell marker expression revealed distinct patient clusters associated with outcome. A cell population neighboring CD8+ T cells, defined by vimentin, ER, and PR expressing epithelial cells, was more prevalent in non-recurrent tumors. Importantly, lower epithelial vimentin expression and lower gene expression of *VIM* associated with worse recurrence-free survival. Loss and low expression of vimentin was validated by IHC as a robust marker for recurrence in FIGO I stage disease and predicted poor prognosis also when including all patients and in endometrioid patients only.

**Interpretation:**

This study reveals distinct characteristics in low-stage tumors and points to vimentin as a clinically relevant marker that may aid in identifying a here to unidentified subgroup of high-risk patients.

**Funding:**

A full list of funding that contributed to this study can be found in the Acknowledgements section.

**Research in context**

**Evidence before this study**

Endometrial cancer is one of the most common gynecological malignancies in the world and despite the good survival rates associated with this disease, recurrent tumors are difficult to treat leading to dramatically reduced survival for these patients. Patients with low-stage tumors are generally treated with surgery alone and most often have good prognosis, but a subgroup experience recurrence. Adjuvant therapy alongside surgery could be beneficial for this patient group, but no robust feature of low-stage tumors at risk of recurrence has been identified, despite multiple studies on the topic.

**Added value of this study**

Several studies report that high vimentin expression is a marker of aggressive disease and worse prognosis in cancer. Conversely, our present study identifies lower expression of vimentin as a biomarker for worse prognosis in low-stage endometrial cancer and points to important differences between cancer types. We report that low-stage endometrial tumors that later recurred show lower epithelial vimentin expression than tumors that did not recur. The finding was validated in two separate gene expression datasets and by immunohistochemical evaluation of epithelial vimentin expression in 518 patients. This study emphasizes tissue specific differences in endometrial cancer and can help better patient outcomes.

**Implications of all the available evidence**

This study reports an important distinction of epithelial vimentin protein expression in endometrial cancers and identifies low vimentin expression as a marker for better patient stratification and precision treatment in a subgroup of patients.

## Introduction

In developed countries, the number of patients diagnosed with endometrial cancer is rising each year, due to longer life expectancies and increased obesity.^1^ The two histological types of endometrial cancer are endometrioid and non-endometrioid. The endometrioid type constitute about 80% and has significantly better prognosis than the non-endometrioid type.^2^ Patients with endometrioid tumors that are diagnosed at low-stages (FIGO stage IA and IB), have good prognosis and surgery is often sufficient treatment. However, 7–10% of low-stage tumors recur, which dramatically reduces survival rates.^3^^,^^4^

Adjuvant therapy may improve outcomes for patients with low-stage, high recurrence-risk endometrioid tumors. Early detection of these patients could inform treatment decisions.^5^ Currently, there are no tools available in the clinic to accurately identify the low-stage endometrioid tumors that will recur. Efforts have been made to reveal markers that may help identify high-risk, low-stage patients. Previous reports have linked both histological features, like MELF (Microcystic, elongated and fragmented) myoinvasion pattern^6^, ^7^, ^8^, ^9^ and genetic alterations, including microsatellite-instability^9^, ^10^, ^11^, ^12^ and CTNNB1 exon 3 hotspot mutation to worse prognosis for low-stage tumors,^13^ however no marker has been identified that robustly predicts risk of recurrence in low-stage tumors. We postulate that the cellular composition of the tumor may affect its ability to spread.

Comprehensive characterization of the single-cell tumor landscape in other cancers has uncovered cellular compositions that can improve patient stratification. In triple negative breast cancer, a patient subgroup enriched with an epithelial phenotype positive for p53 and apoptotic markers showed notably better survival.^14^ The description of distinct immune microenvironments in melanomas revealed that tumors with CD8+ T cells surrounded by human leukocyte antigen DR isotype (HLA-DR) negative myeloid cells were more resistant to immune-checkpoint inhibitors.^15^ In high grade serous ovarian cancer, *BRCA1/2* mutated tumors showed more interaction between T cells and proliferative epithelial cells than wild-type tumors, which may point to differential treatment strategies in clinical subgroups.^16^ Increased knowledge on whether cellular heterogeneity in the tumor microenvironment is associated with risk of recurrence in low-stage endometrial cancer may help give a more optimal patient stratification. This will also aid the pursuit of better and more personalized treatment options for endometrial cancer patients.

We performed imaging mass cytometry (IMC) to study multiple targets simultaneously within a tissue, while preserving the tissue architecture.^17^ We utilized 23 markers in 36 FIGO stage IB endometrioid tumors with similar clinical characteristics and phenotyping of 245,296 single cells to uncover the roles of the tumor-immune- and stromal cell populations in relation to recurrence. We report phenotypically divergent features in the non-recurrent compared to the recurrent tumors with distinct cellular subpopulations. Distinct protein expression associates with clinical outcome in low-stage endometrioid endometrial cancers, suggesting vimentin as a relevant marker to identify patients with poor prognosis.

## Methods

### Patient series and clinical data

Tissue samples were prospectively collected from patients treated for endometrial cancer at Haukeland University Hospital, Bergen, Norway from 2001 to 2015. All patients gave written informed consent before sample collection. Clinical and pathological variables were collected from medical records as previously described.^18^ Tumors were surgically staged according to FIGO 2009 criteria. Patients were treated with hysterectomy with or without adjuvant therapy, according to national protocol. For the IMC analyses, 36 FIGO IB endometrial cancers were selected from the prospectively collected cohort, including 17 recurrent tumors. Recurrence was defined as regrowth of cancer in the vaginal vault, pelvic wall or distant metastasis following primary treatment. For the included tumors, the median time to recurrence was 18 months (range 3–71 months). The recurrent tumors were matched with non-recurrent FIGO IB endometrioid tumors (n = 19) with similar age at diagnosis, histologic grade, body mass index (BMI) and lymph node status. Clinical characteristics for the IMC cohort are given in Supplementary Table S1. Validation of vimentin as a biomarker was performed using immunohistochemical staining (IHC) in pre-operative formalin-fixed samples from a subset of 518 patients from the prospectively collected population-based patient cohort. The study has been approved by the Regional Committee for Medical and Health Research Ethics (REK 2018/594).

### Tissue microarray

Tissue microarrays (TMAs) of hysterectomy specimens (IMC) or pre-operative curettage samples (IHC) from endometrial cancer were constructed as previously described.^19^ Briefly, the area with highest tumor purity was identified on hematoxylin and eosin-stained full sections. Three tissue cylinders per tumor of 0.6 mm diameter were punched out and mounted in a paraffin block using a custom-made precision instrument (Beecher Instruments).

### Antibody conjugation and validation

Antibody conjugation was performed using the Maxpar Antibody labeling kits (Fluidigm), following the manufacturer’s protocol. The concentration was measured using NanoDrop (Thermo Scientific) and the antibody diluted to 0.5 mg/mL in Antibody stabilizer (Candor, Cat# 131050) and stored at 4 °C. In-house conjugated antibodies were validated with immunohistochemistry (IHC) on endometrial tumor tissue to check specificity. All antibodies (Supplementary Table S2) were tested with IMC to define optimal staining concentration on endometrial tumor tissue using the conditions described below.

The antibody panel was designed to include relevant markers for endometrial cancer (ER, PR, p53 and B-catenin), general cancer markers (cytokeratin, vimentin, E-cadherin, Ki-67, pERK1/2, pS6 and VEGF), stromal markers (aSMA, CD31, Podoplanin and Collagen type I) and immune cell markers (CD45, CD4, CD68, CD20, CD3 and CD8a). Anti-Histone H3 and Intercalator-Iridium (Supplementary Table S2) were used to identify cell nuclei.

### Imaging mass cytometry

TMA slides were incubated at 60 °C for 2 h before being deparaffinized in xylene, rehydrated in a series of ethanol (100%, 95%, 80%, 70% and 50%) and washed in Maxpar water (Fluidigm, Cat# 201069). For antigen retrieval, slides were boiled for 30 min in Tris buffer (pH 9, Dako, Cat# S2367), then washed in Maxpar water and TBS (2 × 10 min) before blocking using Superblock (Thermo Scientific, Cat# 37581) for 30 min. Slides incubated with the antibody panel (Supplementary Table S2) diluted in TBS, 0.1% Triton X-100 and 1% BSA at 4 °C over night and washed in 0.1% Triton 100-x TBS and TBS (2 × 8 min). For visualization of DNA, slides were stained with 0.42 μM Intercalator-Ir (Supplementary Table S2). Slides were washed in Maxpar water and air-dried for 20 min. Four samples were excluded due to missing or poor tissue quality. For the remaining cores, high dimensional images were generated by ablating tumor tissue using Hyperion Imaging System (Fluidigm) at a frequency of 200 Hz. Four TMA cores were ablated first and processed to verify the staining and image processing pipeline. Then one TMA core from each tumor was ablated. A second TMA core was ablated from the same tumors 6 months later in order to increase cell numbers for analysis power. Batch correction was performed on signal intensities of all cells using the range algorithm in the Cydar package in R/RStudio,^20^ resulting in reduced batch specific clustering (Supplementary Fig. S1a and b). The range algorithm has been used as unanchored batch correction for mass cytometry data.^21^ No association was found between median signal intensity of all markers per tumor and the patient inclusion year (Spearman correlation = 0.11, p = 0.53), indicating that results are not affected by storage time. In total, 36 samples were evaluated; six samples were represented by one image, 29 samples by two images and one sample by three images.

### Image and data processing

MCD viewer (v. 1.0.560.6, RRID:SCR_023007) was used to visualize and export tiff files of raw data. Images were prepared for single-cell segmentation using an established IMC preprocessing pipeline^22^ and the imctools package (RRID:SCR_017132) in Python (RRID:SCR_001658). A probability map of each image was generated by a pixel classifier in Ilastik (v. 1.3.3, RRID:SCR_015246)^23^ using selected membrane and nuclear markers. Probability maps were segmented in CellProfiler (v. 3.1.9, RRID:SCR_007358)^24^ to produce single-cell masks. Images and corresponding cell masks were imported and arcsinh normalized using a cofactor of 5 in HistoCat (v. 1.76).^25^

### Defining cell clusters and manual gating of major cell populations

Manually gated cell types were defined by gating on scatterplots in HistoCat. CD8+ T cells were gated based on expression of CD8 and CD3. CD4+ T cells were gated based on CD4 and CD3 expression. Proliferative cells were identified by Ki-67 expression. Epithelial cells were defined by expression of E-cadherin, cytokeratin and lack of αSMA. Non-epithelial cells were gated based on expression of αSMA and lack of E-cadherin signal. Expression cut-off was determined individually for each image. Defined populations were mapped back on images of the samples to check the validity of the gating. To extract immune and proliferative cells from the epithelial and non-epithelial compartment, a cut-off was used to identify immune and proliferative cells within the non-epithelial compartment. By subtracting that value from the total immune and proliferative populations, the number of immune cells in the epithelial compartment was estimated. The number of stromal cells was identified by subtracting the immune cells from the non-epithelial cells.

The Rphenograph (RRID:SCR_022603) package was used to perform unsupervised phenograph^26^ clustering of the cells. Markers used for clustering: αSMA, vimentin, cytokeratin, CD31, CD45, ER, CD4, E-cadherin, CD68, p53, CD20, CD8a, VEGF, B-catenin, Podoplanin, Ki-67, collagen type I, CD3, pERK1/2, PR and pS6. Marker enrichment modeling (MEM)^27^ with hierarchical clustering of phenograph clusters was used to define meta clusters. Epithelial meta clusters were identified by using hierarchical clustering of marker expression in phenograph clusters of epithelial cells. Non-epithelial meta clusters were identified by hierarchical clustering of marker expression in phenograph clusters of non-epithelial cells. Hierarchical clustering was used to identify tumor groups based on marker expression of single cells in each tumor. Cells from different images of the same tumor were merged prior to analysis. Cell abundances were calculated as percentages per cluster or sample and visualized using GraphPad (v 8.0.1, GraphPad Software, RRID:SCR_002798) and R/Rstudio (RRID:SCR_001905). Cells directly adjacent to epithelial and CD8+ T cells were identified in HistoCat using the import cell neighbors function, then exported to perform batch correction and phenograph clustering in R/RStudio.

### Gene expression data

Gene expression alterations associated with recurrence were investigated in previously generated microarray gene expression data from 254 primary endometrial tumors.^28^ The microarray dataset is available at ArrayExpress (accession number E-MTAB-2532). For validation of results, TCGA mRNA data of endometrial tumors was downloaded from cbioportal.^29^^,^^30^ To specifically focus on expression levels in epithelial cells, an ESTIMATE (Estimation of Stromal and Immune cells in MAlignant Tumor tissue using Expression)^31^ score was calculated. Low score indicates high tumor purity, indicating high epithelial content. Samples with the lowest 33% ESTIMATE score (n = 83) were used to compare gene expression values. Clinical characteristics of the patient cohort used for gene expression analyses are found in Supplementary Table S3.

### Immunohistochemistry

Unstained TMA sections of pre-operative curettage tissue were deparaffinized in xylene and rehydrated in a series of ethanol (100%, 95%, 80%, 70% and 50%). Antigen retrieval was performed by boiling for 30 min in an antigen retrieval buffer at pH 9 (Dako, Cat# S2367) before peroxidase blocking (Dako, Cat# S2023) for 8 min. The sections were incubated over night at 4 °C with rabbit anti-human vimentin monoclonal antibody (1:200, D21H3, Fluidigm, Cat# 3143027D, RRID:AB_2811069) followed by incubation with anti-rabbit, horseradish peroxidase (HRP)-conjugated secondary antibody (Agilent Technologies, Cat# K4003) for 30 min. Finally, sections were incubated with diaminobenzidine peroxidase (DAB-chromogen; EnVision detection system, Cat# K3468) for 5 min. Sections were counterstained with Hematoxylin (Dako, Cat# S3301) before hydration and mounting.

### Evaluation of immunohistochemistry staining

To evaluate vimentin protein expression from IHC, the semi-quantitative staining index (SI) scoring method was used. SI was calculated by multiplying a staining intensity score (loss = 0, weak = 1, moderate = 2, strong = 3) with the percent area of positive stained tumor tissue (<10% = 1, 10–50% = 2, >50% = 3). Scoring of the tissue was performed blinded for clinical characteristics and only staining of tumor cells was evaluated. Statistics was performed between tumors with loss of vimentin (SI = 0) and vimentin positive tumors (SI = 1–9) and between the lowest quartile (vimentin low tumors, SI = 0–3) and three highest quartiles (vimentin high tumors, SI = 4–9) in the full cohort of 518 patients, a subset of endometrioid tumors only and a subset of FIGO I endometrioid tumors. Clinical characteristics of the patient cohort used for IHC staining are found in Supplementary Table S3.

### Statistical analyses

Statistical analyses were performed with SPSS (v.25, IBM, RRID:SCR_002865) and R/Rstudio. Categorical variables were correlated using the Fisher’s exact test and continuous variables were compared using Mann–Whitney U test. Spearman correlation was used to compare cell numbers per patient with the number of cell types (meta clusters) present in each tumor. The Kaplan–Meier method was used to analyze recurrence-free survival where the difference was calculated using log-rank test (Mantel–Cox). Primary treatment was defined as entry date and patients with death from other causes were censored at the date of death. p-values were two sided and considered significant if p < 0.05.

### Role of funders

Funding sources had no role in study design, data collection, data analysis, interpretation or writing of the manuscript.

## Results

### Distribution of major cell populations does not account for the difference in recurrence

**Fig. 1:**
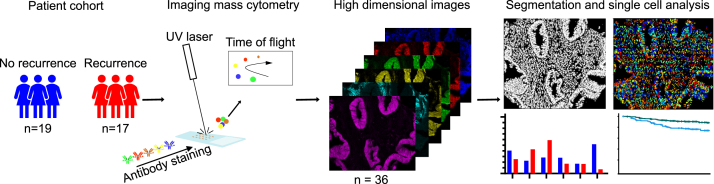
**Workflow of high dimensional image acquisition by imaging mass cytometry.** Tissue from recurrent and non-recurrent FIGO IB endometrial tumors were constructed as tissue microarrays and stained for imaging mass cytometry by a metal-conjugated antibody panel. Tumor tissue was ablated by a UV laser in a 1 μm resolution and ablated particles passed through a time-of-flight detector in a mass cytometer. Resulting high dimensional images were segmented into single cells to perform phenotyping and analyses of the tumor heterogeneity.

**Fig. 2:**
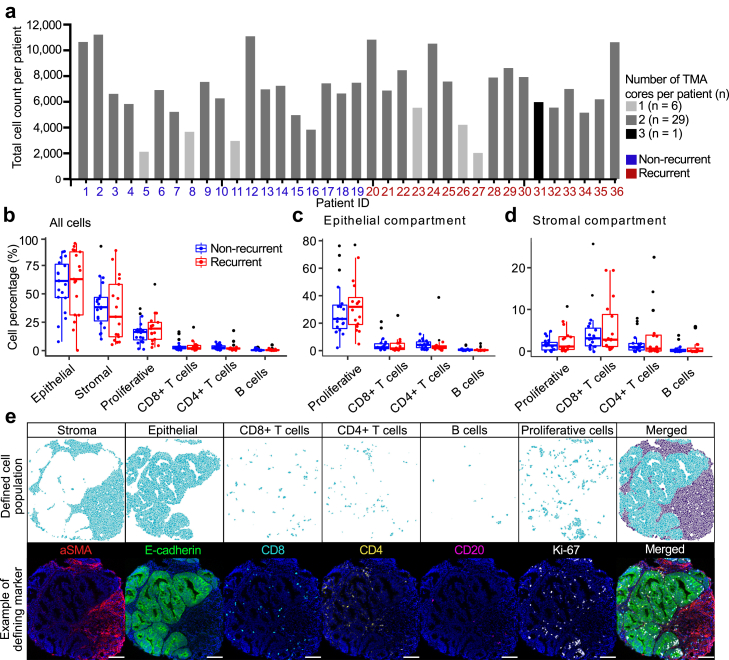
**Cell types were defined by marker expression and were similar between non-recurrent and recurrent tumors.** a) Bar chart showing the number of cells segmented from each patient sample. Patient IDs are indicated in blue for non-recurrent tumors and red for recurrent tumors. Percent abundance of cell types between non-recurrent and recurrent tumors, based on b) all cells, c) cells in the epithelial compartment and d) cells in the stromal compartment. Individual points represent the percentage for each tumor. e) Representative IMC images of a sample with pseudo colors for selected markers used to define cell types mapped back using coordinates on IMC images; α-smooth muscle actin aSMA (red), E-cadherin (green), CD8 (cyan), CD4 (yellow), CD20 (magenta) and Ki-67 (white). Distribution of cell abundance in non-recurrent and recurrent tumors was compared using Mann–Whitney U test. Scale bar in white = 100 μm.

To investigate if cellular composition differed between recurrent and non-recurrent low-stage endometrial carcinomas, tissue images were segmented into single cells (Fig. 1). Segmentation returned data from 245,296 cells from 36 tumors, ranging from 2000 to more than 10,000 cells per tumor (median 6883 cells/tumor; Fig. 2a). Overall, the distribution of manually annotated cell types was similar between non-recurrent and recurrent tumors (Fig. 2b). Epithelial cells were the most abundant cell type with a mean of 58% in non-recurrent and 60% in recurrent tumors. B cells accounted for less than 1% of the total cell abundance in both groups. CD8+ T cells and CD4+ T cells had a mean of 4% and 3%, respectively. The abundance and distribution of immune cells were similar between the epithelial and stromal compartments and did not differ between non-recurrent and recurrent tumors. In non-recurrent tumors, 16% of the cells were proliferative, while 20% were proliferative in recurrent tumors. Most proliferative cells were found in the epithelial compartment (average 30%) compared to the stromal compartment (2%). There was no significant difference in abundance of proliferative cells between non-recurrent and recurrent tumors (Fig. 2c and d). Defined cell populations were visually verified by overlaying the cell populations onto the tissue images (Fig. 2e).

### Unsupervised clustering of single cell data identified multiple cell phenotypes and revealed higher vimentin expression in epithelial cells from non-recurrent tumors

**Fig. 3:**
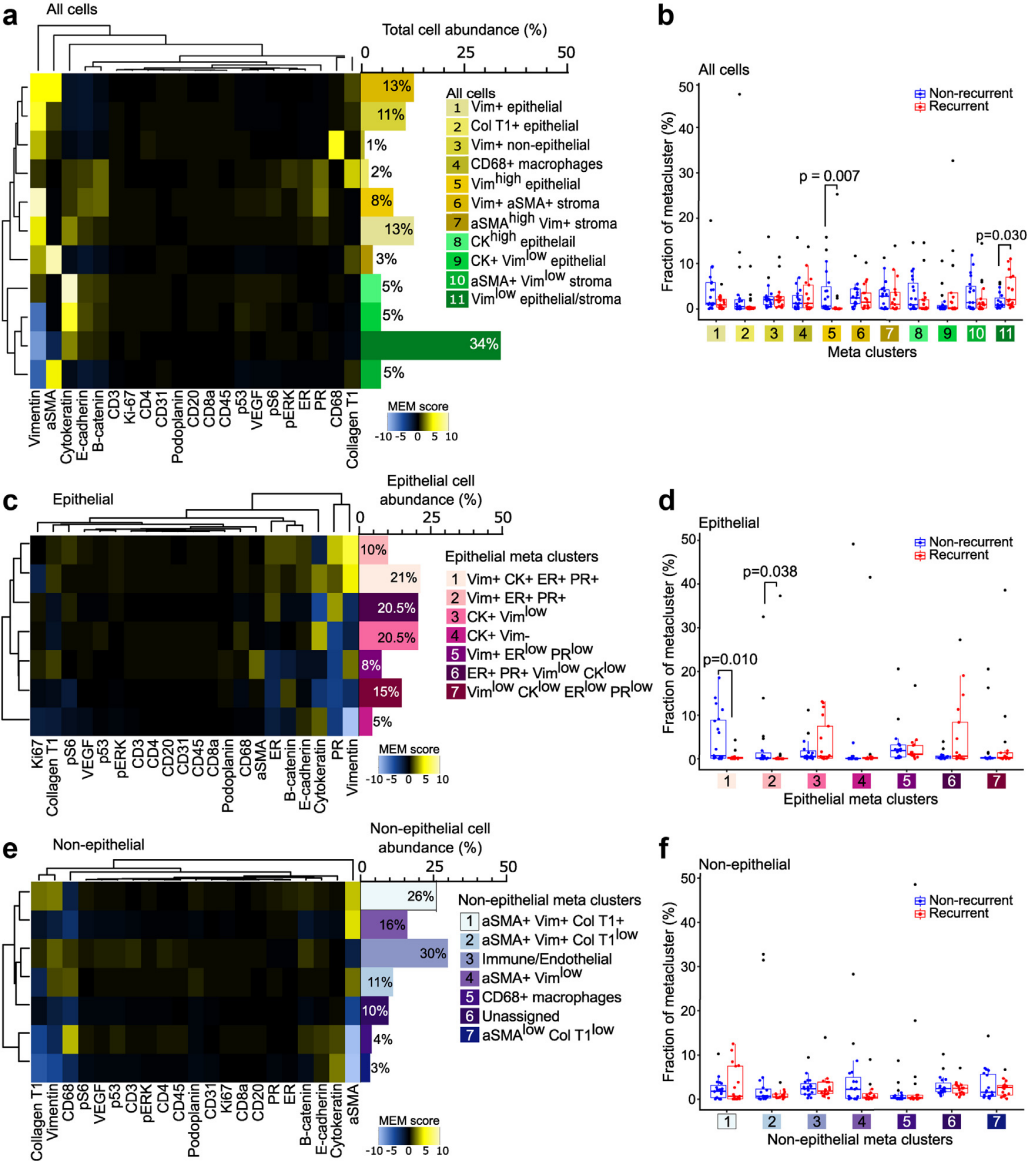
**Single cell clustering revealed higher prevalence of vimentin high epithelial cells in non-recurrent tumors.** Heatmap showing relative protein marker expression of one cell cluster compared to the other clusters with corresponding bar chart, which indicates the percent abundance of each meta cluster from the total cell number for each compartment between non-recurrent and recurrent tumors for a) meta clusters based on all cells, c) meta clusters based on epithelial cells and e) meta clusters based on non-epithelial cells. Comparison of meta cluster abundance between non-recurrent and recurrent tumors for b) all cells, d) epithelial cells and f) non-epithelial cells. Yellow indicates higher expression and blue lower expression. Individual points represent individual tumors. Vim = vimentin, Col T1 = collagen type I and CK = cytokeratin. Distribution of meta clusters in non-recurrent and recurrent tumors was compared using Mann–Whitney U test.

Unsupervised cell phenotyping was performed using phenograph. Thirty-eight cell clusters were identified in a primary clustering based on single cell expression. To identify common cell phenotypes between the tumors, the 38 clusters were subjected to a hierarchical clustering and clusters with similar expression patterns were merged, resulting in 11 meta clusters (Supplementary Fig. S2a; Fig. 3a). No association was seen between the number of cells per patient and the number of cell phenotypes (meta clusters) identified per tumor (Spearman correlation = 0.28, p = 0.098). Meta clusters 1–7 (yellow) had higher expression of vimentin than meta clusters 8–11 (green). Meta cluster five, characterized by high epithelial vimentin expression, was enriched in non-recurrent tumors (Mann–Whitney U test p = 0.007). Meta cluster 11, consisting of epithelial and stromal cells, was enriched in recurrent tumors (Mann–Whitney U test p = 0.030), and was characterized by lower vimentin expression. Meta clusters consisting of mainly stromal cells did not differ markedly between recurrence groups (Fig. 3b).

To further investigate the difference in the cell types of epithelial origin between non-recurrent and recurrent tumors, we performed a new phenograph clustering on epithelial cells only, identifying 36 epithelial clusters. Hierarchical clustering merged the clusters into 7 epithelial meta clusters (Supplementary Fig. S2b; Fig. 3c). Meta cluster 1, 2 and 5 had higher vimentin expression than the other clusters. In addition, meta cluster 1, 2 and 6 had higher ER and PR expression. When comparing the distribution of the epithelial meta clusters, we observed a significant enrichment of meta clusters 1 and 2 in non-recurrent tumors (Mann–Whitney U test p = 0.010 and p = 0.038, respectively, Fig. 3d).

A phenograph clustering on non-epithelial cells identified 27 phenograph clusters that subsequently were merged into seven meta clusters (Supplementary Fig. S2d; Fig. 3e). Meta cluster 1, 2 and 4 were aSMA+ stromal cells. T cells, B cells, endothelial and proliferative cells were clustered together in meta cluster 3, while meta cluster 5 was CD68+ macrophages. No distinct differences in abundance of the non-epithelial meta clusters were found between non-recurrent and recurrent tumors (Fig. 3f).

### Clustering of tumors based on single cell expression revealed distinct tumor groups associated with patient outcome

**Fig. 4:**
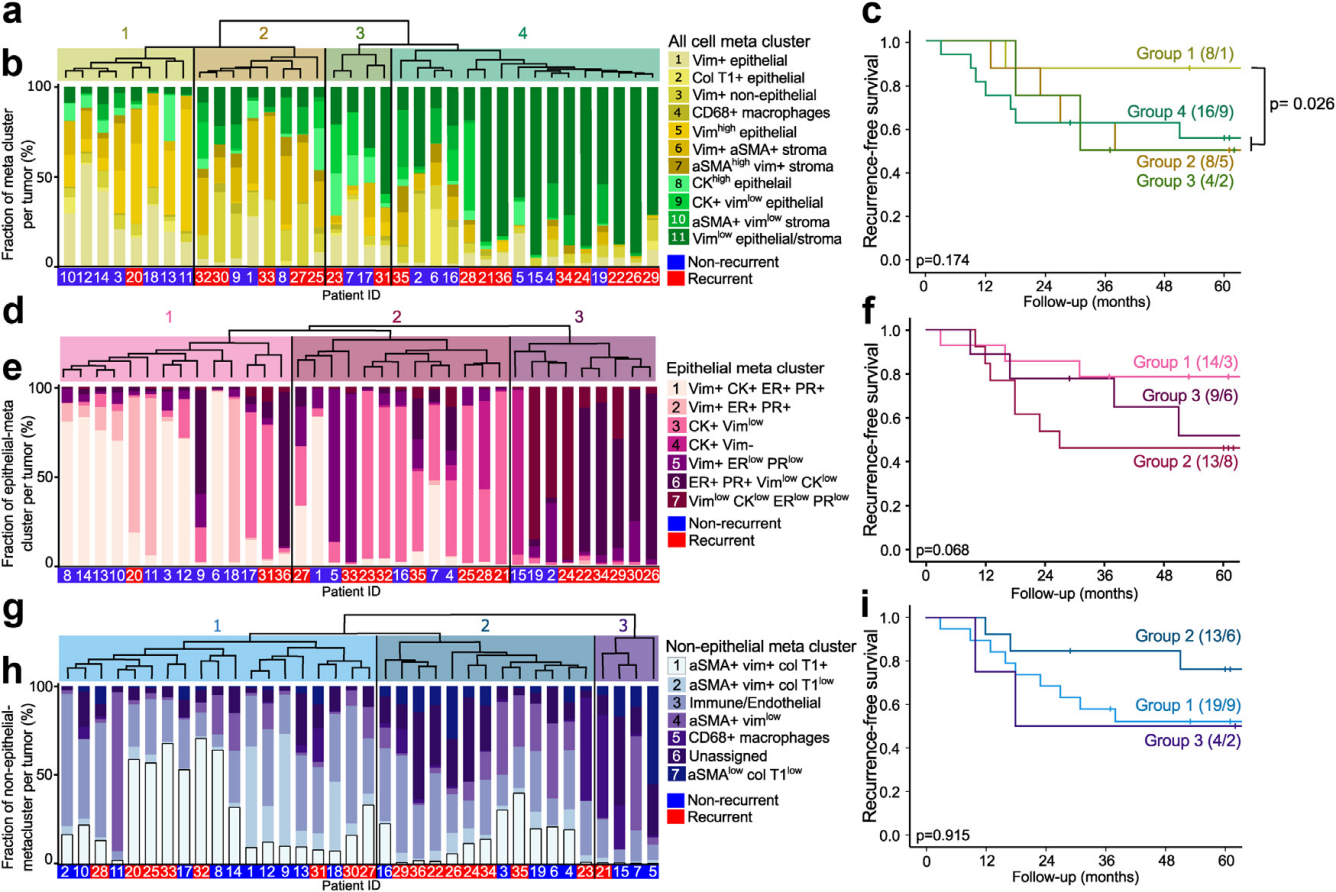
**Higher tumor fraction of vimentin high phenotypes associated with better recurrence-free survival.** Hierarchical clustering of tumors based on single cell expression with corresponding bar plots showing the meta cluster distribution and Kaplan–Meier curves of recurrence-free survival between tumor groups identified by the hierarchical clustering for a, b, c) all cells, d, e, f) epithelial cells and g, h, i) non-epithelial cells. Kaplan–Meier survival curves presented with number of patients in each group and number of events in parentheses (patients/events). p-values from Mantel Cox log-rank test. Vim = vimentin, Col T1 = collagen type I and CK = cytokeratin.

To identify patient groups with similar cellular composition and phenotypes, tumors were clustered based on cellular marker expression. To visualize differential cellular composition, the previously defined meta clusters were indicated for individual tumors. Analyses were performed both for all cells, epithelial cells only and non-epithelial cells only. When analyzing all cells, four tumor groups were identified by hierarchical clustering based on similar single-cell expression patterns within the tumors (Fig. 4a). Tumors in group 1 had higher abundance of cells in meta cluster 1 and 5 (vimentin high epithelial cells) than the other tumor groups (Fig. 4b). Tumors in group 2 had a higher number of cells in meta cluster 3 and 6 (vimentin+ stroma cells), while tumors in group 3 had a more diverse distribution of the meta clusters. Most of the tumors in group 4 had higher numbers of cells from meta cluster 11 (vimentin^low^ cells; Fig. 4b). Overall, there was no difference in recurrence-free survival between the tumor groups. When comparing tumor group 1 with the other groups combined, we observed a significantly better recurrence-free survival of patients with tumors in group 1 (Fig. 4c). When analyzing epithelial cells only, we identified 3 tumor groups (Fig. 4d). Most tumors in group 1 had a higher number of cells in meta cluster 1 (vimentin+ cytokeratin+ ER+ PR+) and meta cluster 2 (vimentin+ ER+ PR+; Fig. 4e). Tumors in group 2 had higher abundance of cells in meta cluster 3 (cytokeratin+ vimentin^low^) and two tumors had higher numbers of cells in meta cluster 5 (vimentin+ ER^low^ PR^low^), while tumors in group 3 had high numbers of cells in meta cluster 6 (ER+ PR+ vimentin^low^ cytokeratin^low^) and 7 (cytokeratin^low^ vimentin^low^ ER^low^ PR^low^; Fig. 4e). Patients with tumors in group 2, although not statistically significant, tended to have worse recurrence-free survival than group 1 and 3 (Fig. 4f). In the non-epithelial clustering, we identified 3 tumor groups (Fig. 4g). Most tumors in group 1 were characterized by high numbers of cells in non-epithelial meta cluster 1, 2 and 3, mostly aSMA and vimentin+ stroma cells (Fig. 4h). Tumors in group 2 had higher number of cells in meta cluster 1, 3, 4 and 6, while group 3 was characterized by higher number of cells in meta cluster 4, 5, 6 and 7 (Fig. 4h). The non-epithelial tumor groups were not associated with patient outcome (Fig. 4i).

### Non-recurrent tumors have a higher number of a vimentin, ER and PR positive epithelial cell population directly adjacent to CD8+ T cells

**Fig. 5:**
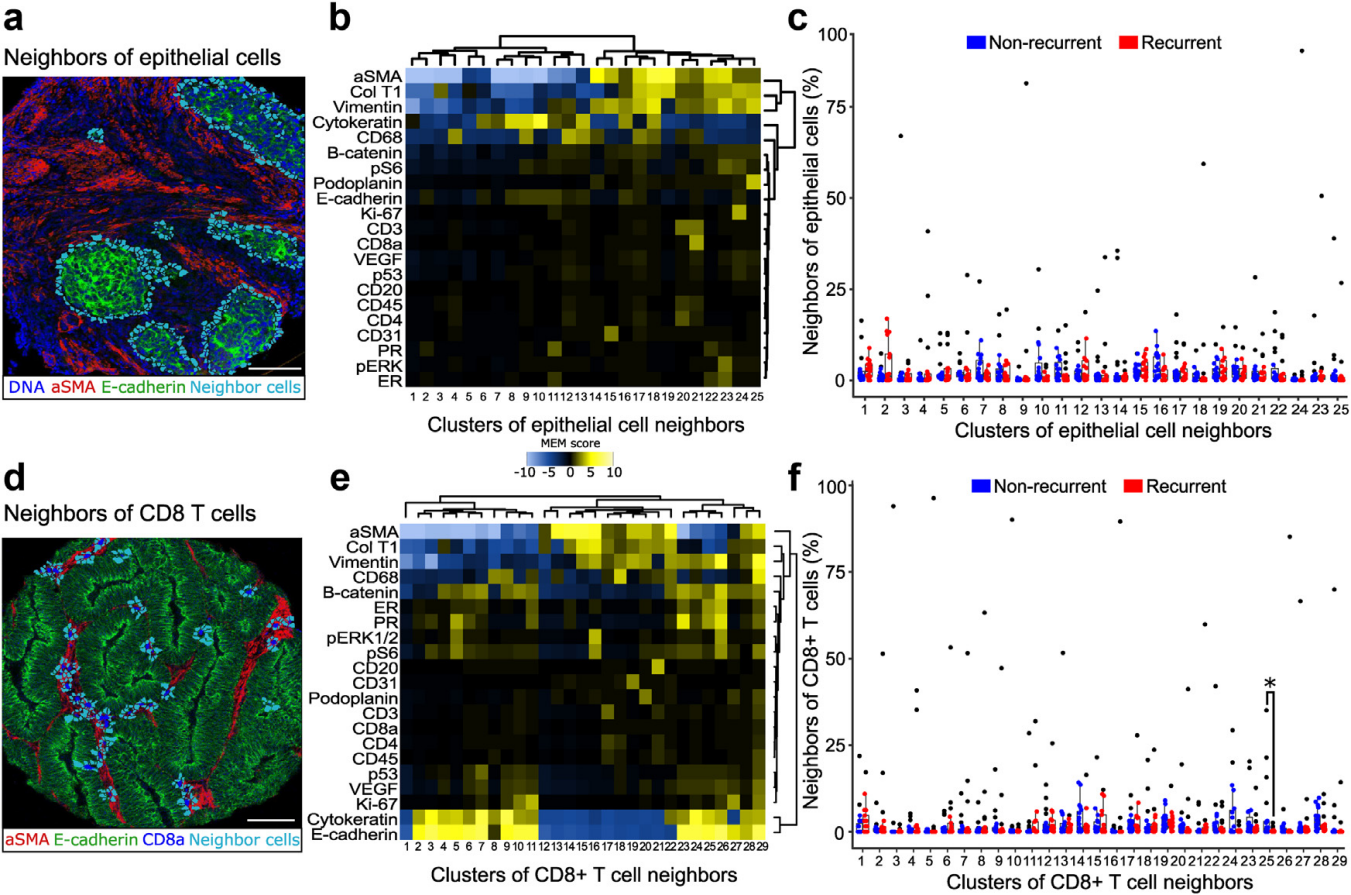
**Characterization of neighboring cell types of epithelial and CD8+ T cells revealed a higher number of a vimentin and ER/PR+ epithelial cell type directly adjacent to CD8+ T cells in non-recurrent tumors.** a) Neighbors of epithelial cells are shown in teal and superimposed on a selected tissue image. b) Heatmap showing the relative expression levels of epithelial neighbor cells in each cluster. Yellow indicates higher expression, while blue indicates lower expression. c) The percent abundance of each epithelial neighbor cell cluster compared between non-recurrent (blue) and recurrent tumors (red). d) Neighbors of CD8+ T cells are shown in teal, superimposed on a selected tissue image. e) Heatmap showing the relative expression levels CD8+ T cell neighbor cells in each cluster. f) The percent abundance of each cluster compared between non-recurrent and recurrent tumors. Points represent values of individual tumors. Col T1 = collagen type I. Distribution of clusters in non-recurrent and recurrent tumors was compared using Mann–Whitney U test (∗p < 0.05). Scale bar in white = 100 μm.

We characterized cell types directly adjacent to epithelial cells to investigate if the immediate epithelial environment could provide information on disease aggressiveness. The neighboring cells were identified (Fig. 5a) and 25 clusters were found by phenograph clustering (Fig. 5b). Most of the cell clusters adjacent to epithelial cells were of stromal character, but we also identified CD8+ and CD4+ T cells, endothelial cells, lymphovascular cells and proliferative cells. The percent abundance of the cell clusters between non-recurrent and recurrent tumors was similar between the two groups (Fig. 5c). We characterized and clustered cells directly adjacent to CD8+ T cells to investigate if the CD8 T cell neighborhood was different in recurrent and non-recurrent tumors (Fig. 5d). Twenty-nine clusters were identified and the clusters consisted of epithelial-, stromal- and other immune cell phenotypes (Fig. 5e). Of these, cluster 25 was significantly enriched in non-recurrent tumors and was characterized by higher expression of vimentin, ER, PR and E-cadherin (Fig. 5f).

### Vimentin expression is lower in recurrent tumors and significantly associates with worse recurrence-free survival

**Fig. 6:**
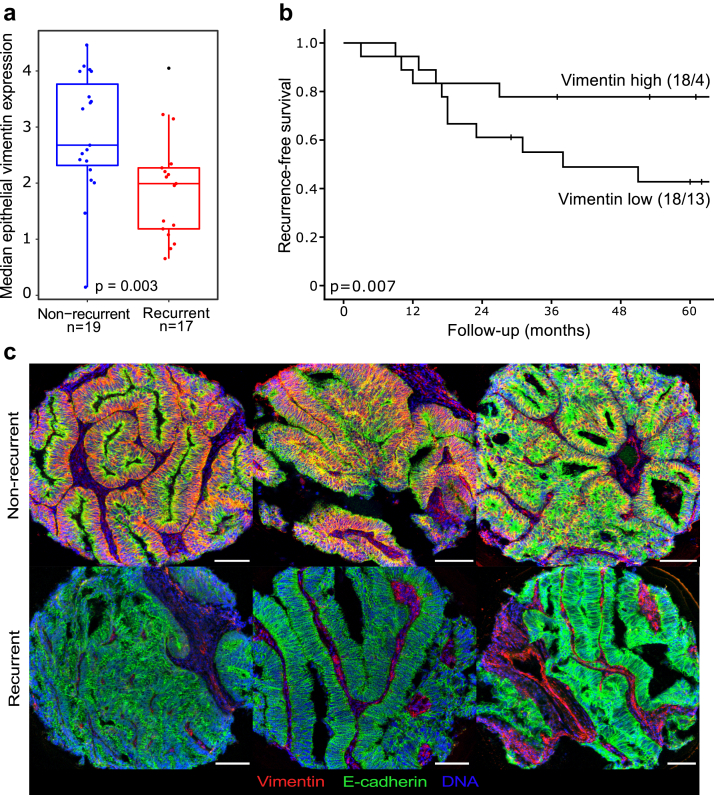
**Lower epithelial vimentin expression was associated with recurrent tumors.** a) Comparison of median epithelial vimentin expression from IMC data in non-recurrent and recurrent tumors. p-value from Mann–Whitney U test. b) Kaplan–Meier of recurrence-free survival of tumors divided into two groups based on median epithelial vimentin expression. Survival curves presented with number of patients and number of events in parentheses (patients/events). p-values from Mantel Cox log-rank test. c) Selected IMC images with pseudo colors of vimentin (red), E-cadherin (green) and DNA (blue) of non-recurrent tumors (top row) and recurrent tumors (bottom row). Scale bar in white = 100 μm.

To investigate if epithelial expression of single markers was different between recurrent and non-recurrent tumors, median single-maker intensities were calculated. The median vimentin intensity in epithelial cells was significantly lower in recurrent tumors (Mann–Whitney U test p = 0.003; Fig. 6a and c). Besides vimentin, only epithelial PR expression was significantly different (Mann–Whitney U test p = 0.049), with lower expression in recurrent tumors. As the strong association between low vimentin expression and recurrence was intriguing, we focused our following analyses on vimentin. All tumors were dichotomized based on the epithelial vimentin expression to vimentin high and vimentin low. Patients with vimentin low tumors had significantly worse recurrence-free survival (Fig. 6b).

**Fig. 7:**
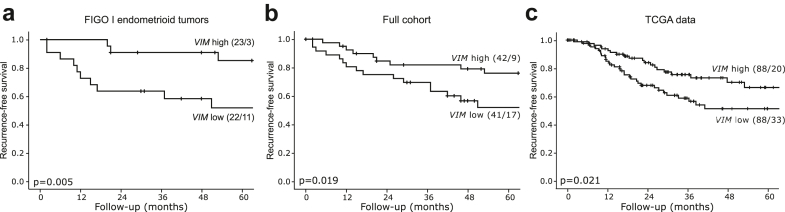
**Lower gene expression of *VIM* associated with worse recurrence-free survival.** Kaplan–Meier curves of recurrence-free survival between patients with *VIM* high and *VIM* low tumors with high epithelial component for a) FIGO I endometrioid tumors (n = 45), b) for all histologic types and FIGO stages (n = 84) and c) TCGA mRNA endometrial cancer data (n = 176). Kaplan–Meier survival curves presented with number of patients and number of events in parentheses (patients/events). p-values from Mantel Cox log-rank test.

To further explore the vimentin expression in endometrial tumors, we investigated *VIM* mRNA expression in an independent gene expression dataset from our population-based cohort (n = 254). Samples with high epithelial component defined by the ESTIMATE score were selected (n = 83). Lower expression of *VIM* was significantly associated with worse recurrence-free survival in the FIGO I endometrioid subgroup (n = 45; Fig. 7a), as well as in all patients (n = 83; Fig. 7b). These findings were validated in the TCGA dataset of endometrial tumors (n = 176), where patients with high epithelial component and low *VIM* tumors had significantly worse recurrence-free survival (Fig. 7c).

### Low vimentin expression in pre-operative samples is associated with worse recurrence-free survival

**Fig. 8:**
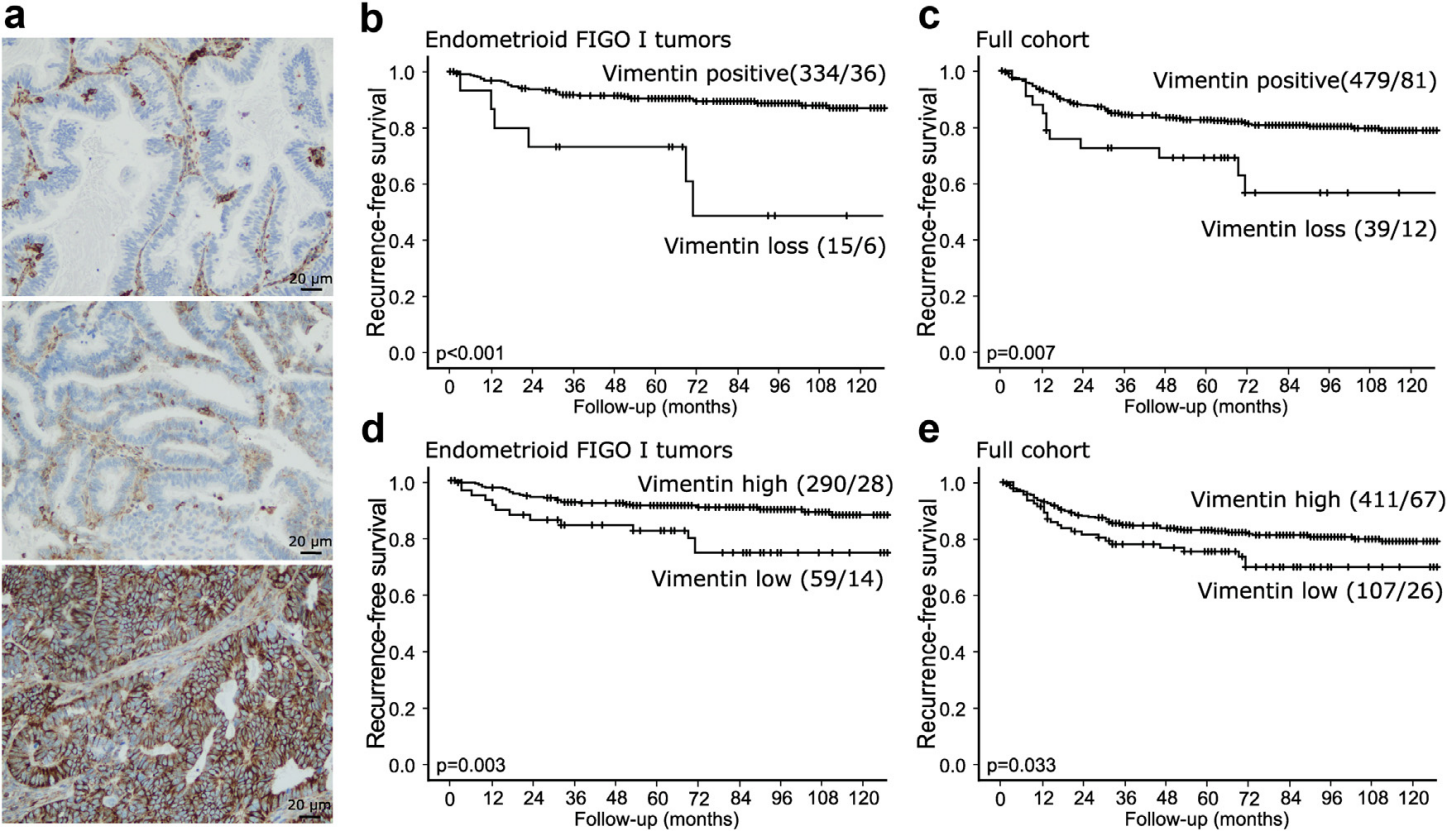
**Low vimentin expression in pre-operative samples are associated with worse recurrence-free survival.** a) Example images of endometrial tumors with loss of vimentin expression in epithelial cells (top panel), weak expression of vimentin in epithelial cells (middle panel) and high expression of vimentin in epithelial cells (bottom panel). Scale bar = 20 μm. Kaplan–Meier curves of recurrence-free survival between patients with complete vimentin loss and vimentin positive epithelial cells for b) FIGO I endometrioid tumors only (n = 349) and c) the full cohort of all types of endometrial cancers (n = 518). Kaplan–Meier curves of recurrence-free survival for patients with low or high expression of vimentin in epithelial cells for d) FIGO I endometrioid tumors only (n = 349) and e) the full cohort (n = 518). Kaplan–Meier survival curves presented with number of patients and number of events in parentheses (patients/events). p-values from Mantel Cox log-rank test.

To further explore the robustness of vimentin as a prognostic marker in endometrial cancer, pre-operative curettage samples from 518 patients in our population-based cohort were assessed for epithelial vimentin protein expression by immunohistochemistry. Examples of loss, weak and strong expression are given in Fig. 8a. Patients with complete loss of vimentin expression in epithelial cells had significantly worse recurrence-free survival compared to patients with vimentin positive tumors in the FIGO I endometrioid tumors (Log rank p < 0.001; Fig. 8b). Interestingly, loss of vimentin was also highly significantly associated with poor recurrence-free survival when including all histologic types of endometrial cancers (Log rank p = 0.007; Fig. 8c) and in the subgroup of patients with endometrioid tumors (Log rank p = 0.005, data not shown). Similar prognostic effect was seen when using the lowest quantile as cut-off, where low expression (Fig. 8a, middle panel) associated significantly with poor recurrence-free survival in all analyses (Fig. 8d; FIGO I endometrioid patients Log rank p = 0.003, Fig. 8e; full cohort Log rank p = 0.033 and endometrioid only Log rank p = 0.032, data now shown).

## Discussion

Most patients with endometrioid endometrial FIGO I tumors have good prognosis and are often cancer free following hysterectomy, however some of these patients experience recurrence. Patients with recurrence have a dramatically worse prognosis and are difficult to treat. Identifying markers of poor prognosis in low-stage tumors at primary diagnosis may improve the identification of tumors with recurrence potential. Several recent reports have identified single-cell features that associate with prognosis in other tumors.^14^, ^15^, ^16^ Here, we report single cell analysis of low-stage endometrioid tumors, aiming to uncover cellular compositions that may identify low-stage tumors with a higher risk of recurrence. We did not detect any differences in distribution of major cell populations including immune cells and stromal cells between recurrence groups. Expression patterns in epithelial cells are associated with patient outcome, while patterns in non-epithelial cells are not. Importantly, we identified vimentin expression in epithelial cells to be a robust marker for recurrence in low-stage endometrial cancer.

Our analyses did not reveal any significant difference in stromal phenotypes between non-recurrent and recurrent tumors. Although this finding is affected by the included markers in our panel, we included aSMA, vimentin and collagen type 1, all typical markers for cancer associated fibroblasts (CAFs). CAFs are one of the most abundant stromal cell types in solid tumors and have been linked to tumor progression and resistance to therapy. Whether secretion of growth factors (TGFβ, GAS6, FGF5 and HGF), cytokines and chemokines (IL-6, CXCL9) from CAFs,^32^ may affect aggressiveness and thereby associate with recurrence of endometrial cancer, is not known and could be investigated in future studies. Similar results were found when investigating the abundance of infiltrating immune cells. We did not detect any differences of immune cell abundance in epithelial or stromal compartments or from total cell numbers between recurrent and non-recurrent tumors. Immune cell infiltration, especially CD8+ T cells, is generally associated with favorable prognosis in cancer,^33^ but recent reports reveal inconsistencies of their prognostic value in endometrial cancer.^34^^,^^35^ Our findings could indicate that the extent of immune cell infiltration within areas of high tumor purity is not associated with risk of recurrence. This is in line with a previous study, where only the abundance of CD8+ T cells in the immediate invasive front was associated with patient outcome.^36^ Further studies with larger patient cohorts using stromal and immune cell specific antibody panels could elucidate the role of specific cell subsets in recurrence of endometrial cancer.

Among the markers included in our panel, vimentin expression was prominent in the stratification of cell phenotypes and was highly expressed in epithelial cells in non-recurrent tumors. In contrast, we did not observe any association with recurrence in relation to protein expression levels of the other markers, also including B-catenin, although mutations in the *CTNNB1* gene have been linked to low-stage recurrence in endometrial cancer.^13^ Vimentin is a member of the type III intermediate filament protein family and is involved in organizing the cytoplasmic space and cell motility.^37^ Contrary to our data, higher expression of vimentin has been linked to epithelial-mesenchymal transition (EMT) and more aggressive disease in several cancers, including endometrial cancer.^38^, ^39^, ^40^, ^41^, ^42^, ^43^, ^44^, ^45^ Inhibition of caspase-induced proteolysis of vimentin through phosphorylation by AKT1 has been suggested to be a contributing factor for increased migration and invasiveness in several cancers.^46^, ^47^, ^48^ We did not observe any elevated phosphorylation of S6, suggesting that increased activation in the AKT pathway is not present in vimentin high cells in tumors included in this study.

Vimentin is also expressed in stromal cells and has been used solely as a stromal cell marker in endometrial cancer research.^49^ In our study, vimentin and E-cadherin expression co-localized in the epithelial cells and there was no decrease in E-cadherin expression in vimentin high tumors. Loss of E-cadherin and gain of vimentin expression is a feature usually linked to EMT.^39^ This indicates that vimentin expression is not related to increased invasiveness and could be a poor marker for EMT in endometrial cancer. We also observed a higher number of an epithelial cell phenotype expressing vimentin, ER and PR directly adjacent to CD8+ T cells in non-recurrent tumors. CD8+ T cells are cytotoxic and positioned directly next to the cancer cells could indicate a greater anti-tumor potential.^50^

To further support our results, we validated our findings in a local independent mRNA dataset and the TCGA mRNA dataset of endometrial cancer, focusing on tumors with high epithelial component. In concordance with protein levels detected by IMC, lower *VIM* expression was associated with worse recurrence-free survival in both mRNA data sets.

Unfavorable prognosis in relation to lower vimentin expression in tumor epithelial cells may be tissue specific considering higher epithelial vimentin expression usually is linked to EMT and metastases in cancer.^43^, ^44^, ^45^ To further investigate whether low expression of vimentin can have clinical importance in endometrial cancer, we evaluated vimentin expression by IHC in pre-operative lesions from 518 endometrial cancer patients, including all types and grades. Our data clearly shows that loss of vimentin expression is highly prognostic in the subset of patients with FIGO I tumors as well as in endometrioid patients and in the full cohort. Our data is in line with previous reports^51^^,^^52^ describing an association between loss of vimentin expression and non-endometrioid tumors, higher FIGO stage, higher tumor grade and worse patient outcome and we here add important information on vimentin in low-stage tumors. Our data also indicate that the cut-off for detecting low expression of vimentin is broad, as both loss and low expression (lowest quartile) predict poor prognosis, further supporting the robustness of this marker to identify patients at risk of recurrence. In our cohort, complete loss was more significant in all subgroup analyses but the cut-off for low vimentin should be validated in larger, independent cohorts. As in particular the FIGO I patient subgroup lack good makers to identify high-risk tumors, detection of vimentin in pre-operative tissue could help identify patients in need of more aggressive treatment and closer follow-up. Vimentin is expressed in normal endometrial epithelial cells^53^^,^^54^ and has been used to distinguish endometrial cancer from ovarian cancer,^55^ illustrating tissue specific differences. Worse patient prognosis with decreased vimentin expression may indicate that endometrial cancer cells with lower levels of vimentin are less similar to normal endometrial cells and are more malignant. The functional mechanism of vimentin in endometrial cancer should be further explored.

Our analyses have revealed cellular heterogeneity in low-stage endometrioid tumors and confirmed that the cellular composition is relevant for patient prognosis. Most importantly, low epithelial vimentin expression is associated with high-risk of recurrence in low-stage endometrial cancer, an important distinction from other cancer types where vimentin is linked to worse patient outcome.^38^ Epithelial vimentin expression is a robust marker that could aid in identifying low-stage patients with higher risk of recurrence.

## Contributors

H.E.L., I.S.H., L.A.A. and C.K. conceived the experiment. H.E.L. conducted the experiments. J.T. contributed to collection of samples and clinical data. H.E.L., H.F.B., M.K.H., L.A.A. and C.K. contributed to the interpretation of the results. H.E.L. and C.K. wrote the manuscript. C.K. supervised the project. H.E.L. and C.K. have verified the underlying data. All authors provided feedback on the research and revision of the manuscript. All authors read and approved the final version of the manuscript.

## Data sharing statement

Data supporting the findings of this study can be obtained from the corresponding author upon reasonable request.

## Declaration of interests

The authors declare no competing interests.

## Appendix ASupplementary data

Supplementary Material Supplementary Figure S1: Range correction of signal intensities corrected for batch effects in IMC data. a) Left panel show histogram of uncorrected E-cadherin signal of three different batches. Batch 1 was collected first, batch 2 collected second and batch 3 collected last. The right panel show unanchored range corrected E-cadherin signal for all three batches. b) Left plot show tSNE of uncorrected intensity value, colored by batch. Right plot show tSNE of range corrected intensity values with reduced batch effects, colored by batch. Supplementary Figure S2: Hierarchical clustering of phenograph clusters were used to identify meta clusters. Heatmap showing relative marker expression of phenograph clusters for a) all cells, b) epithelial cells and c) non-epithelial cells. The right panel on each heatmap indicates the different meta cluster annotations. Yellow indicates higher expression and blue lower expression.

Combined-datasheets
