# Corrigendum to “Single-cell profiling of low-stage endometrial cancers identifies low epithelial vimentin expression as a marker of recurrent disease” [EBioMedicine 92 (2023) 104595]

**DOI:** 10.1016/j.ebiom.2023.104876

**Published:** 2023-11-15

**Authors:** Hilde E. Lien, Hege F. Berg, Mari K. Halle, Jone Trovik, Ingfrid S. Haldorsen, Lars A. Akslen, Camilla Krakstad

**Affiliations:** a Centre for Cancer Biomarkers, Department of Clinical Science, University of Bergen, Bergen, Norway; b Department of Gynecology and Obstetrics, Haukeland University Hospital, Bergen, Norway; c Mohn Medical Imaging and Visualization Centre, Department of Radiology, Haukeland University Hospital, Bergen, Norway; d Section for Radiology, Department of Clinical Medicine, University of Bergen, Bergen, Norway; e Department of Pathology, Haukeland University Hospital, Bergen, Norway

The authors wish to point out an inadvertent error in Fig. 4 in the published article. The Kaplan–Meier curve given in Fig. 4i does not correspond to the clusters in 4 g/h. The correct Fig. 4i is presented below. The authors would like to emphasize that the error does not affect the description, interpretation or original conclusions. The authors sincerely apologize for any inconvenience caused by this error.

**Fig. 4:**
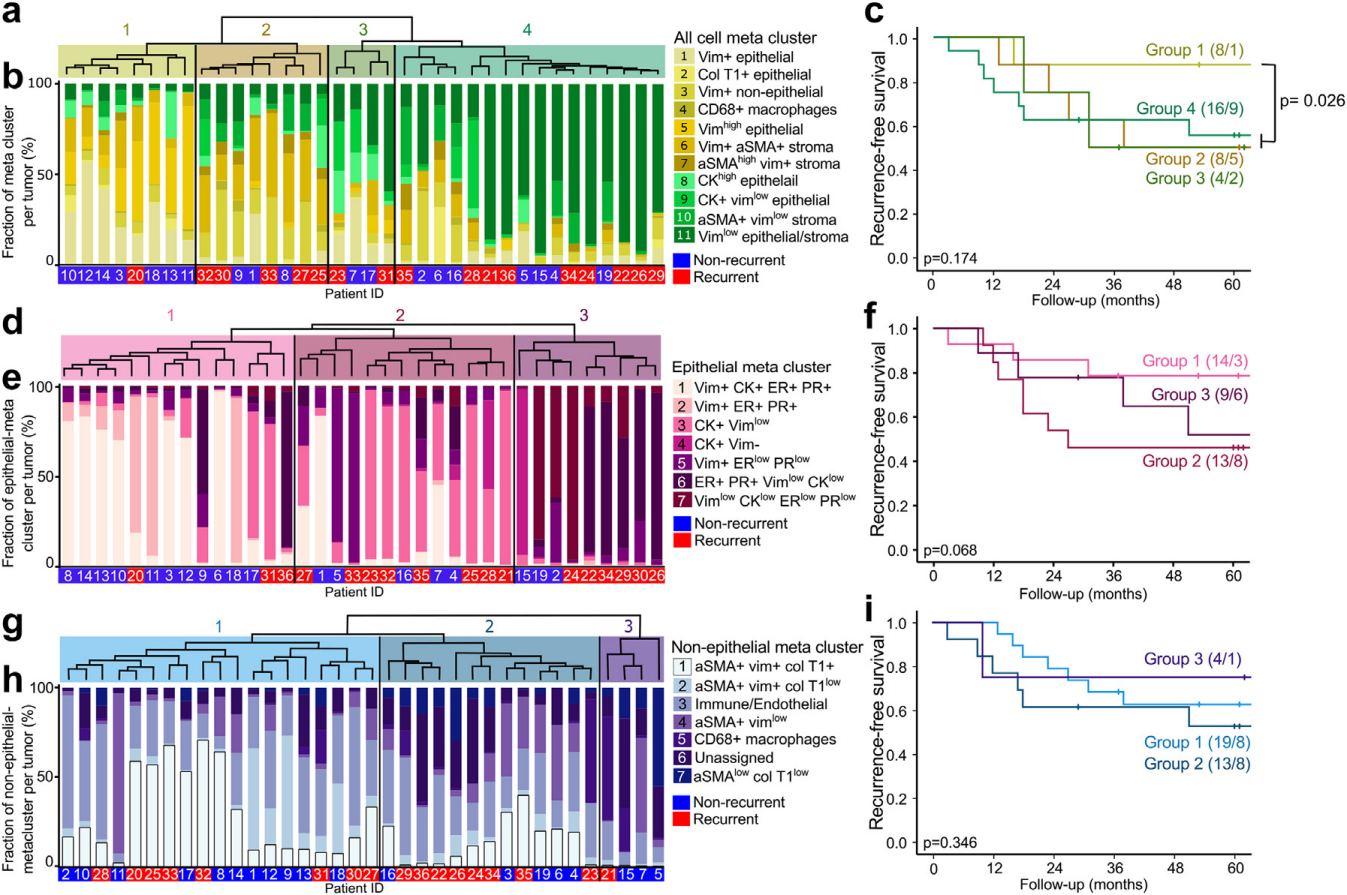
**Higher tumor fraction of vimentin high phenotypes associated with better recurrence-free survival.** Hierarchical clustering of tumors based on single cell expression with corresponding bar plots showing the meta cluster distribution and Kaplan–Meier curves of recurrence-free survival between tumor groups identified by the hierarchical clustering for a, b, c) all cells, d, e, f) epithelial cells and g, h, i) non-epithelial cells. Kaplan–Meier survival curves presented with number of patients in each group and number of events in parentheses (patients/events). p-values from Mantel Cox log-rank test. Vim = vimentin, Col T1 = collagen type I and CK = cytokeratin.

