# Supplementary material for: Single-cell profiling of low-stage endometrial cancers identifies low epithelial vimentin expression as a marker of recurrent disease
Source: eBioMedicine. 2023 May 3;92:104595. doi: 10.1016/j.ebiom.2023.104595 (PMC10277918; doi:10.1016/j.ebiom.2023.104595)
Supplement: Supplementary Material — Supplementary Figure S1: Range correction of signal intensities corrected for batch effects in IMC data. a) Left panel show histogram of uncorrected E-cadherin signal of three different batches. Batch 1 was collected first, batch 2 collected second and batch 3 collected last. The right panel show unanchored range corrected E-cadherin signal for all three batches. b) Left plot show tSNE of uncorrected intensity value, colored by batch. Right plot show tSNE of range corrected intensity values with reduced batch effects, colored by batch. Supplementary Figure S2: Hierarchical clustering of phenograph clusters were used to identify meta clusters. Heatmap showing relative marker expression of phenograph clusters for a) all cells, b) epithelial cells and c) non-epithelial cells. The right panel on each heatmap indicates the different meta cluster annotations. Yellow indicates higher expression and blue lower expression. [file mmc1.pdf]

Supplementary material

Table of content:

p.2 – Supplementary table 1

p.3 – Supplementary table 2

p.4 – Supplementary table 3

p.5 – Supplementary figure 1

p.6 – Supplementary figure 2

Supplementary tables and figures

Supplementary table 1 Clinical characteristics of patients with non-recurrent and recurrent tumors in the IMC cohort

Characteristic	Non-recurrence, n=19 n(%)	Recurrence, n=17 n(%)	P-value
**Age (years)**			0.156
<66	8 (42)	3 (18)	
≥66	11 (58)	14 (82)	
**Body mass index (BMI)**			0.169
<30	10 (52)	13 (76)	
≥30	5 (27)	1 (6)	
Missing	4 (21)	3 (18)	
**Histologic type**			-
Endometrioid	19 (100)	17 (100)	
Non-endometrioid	0	0	
**Histologic Grade**			0.482
I	9 (47)	5 (29)	
II	7 (37)	10 (59)	
III	3 (16)	2 (12)	
**Myometrial infiltration**			-
<50%	0	0	
≥50%	19 (100)	17 (100)	
**Lymph node metastasis**			-
No	16 (84)	13 (76)	
Yes	0	0	
Missing	3 (16)	4 (24)	
**Adjuvant treatment**			0.462
No additional treatment	17 (90)	12 (70)	
External radiation	1 (5)	2 (11)	
Chemotherapy	1 (5)	2 (11)	
Chemoradiation	0	1 (6)	
**Follow-up time, months**			
Mean (min-max)	137.05 (37-226)	90.24 (14-192)	

Supplementary table 2 IMC panel of metal tagged antibodies

Tag	Target	Clone	Dilution (μg/ml)	Vendor	Cat#	RRID
**141Pr**	Alpha-SMA	1A4	2.5	Fluidigm	3141017D	AB_2890139
**143Nd**	Vimentin	D21H3	5	Fluidigm	3143027D	AB_2811069
**148Nd**	Pan-cytokeratin	AE1/AE3	10	Fluidigm	3148022D	AB_2927682
**151Eu**	CD31	EPR3094	5	Fluidigm	3151025D	AB_2890140
**152Sm**	CD45	CD45-2B11	6.67	Fluidigm	3152016D	AB_2927683
**154Sm***	ER	SP1	20	Abcam	ab187260	AB_2927684
**156Gd**	CD4	EPR6115	5	Fluidigm	3156033D	AB_2811051
**158Gd**	E-Cadherin	24E10	6.67	Fluidigm	3158029D	AB_2893074
**159Tb**	CD68	KP1	6.67	Fluidigm	3159035D	AB_2810859
**160Gd***	p53	DO-7	20	Cell signaling	48818BF	AB_2713958
**161Dy**	CD20	H1	2.5	Fluidigm	3161029D	AB_2811016
**162Dy**	CD8a	D8A8Y	10	Fluidigm	3162035D	AB_2909535
**163Dy**	VEGF	G153-694	10	Fluidigm	3163028D	AB_2927685
**165Ho**	β-Catenin	D13A1	6.67	Fluidigm	3165032D	AB_2909539
**167Er***	Podoplanin	D2-40	10	Biolegend	916606	AB_2565820
**168Er**	Ki-67	B56	5	Fluidigm	3168022D	AB_2811061
**169Tm**	Collagen Type I	Polyclonal	5	Fluidigm	3169023D	AB_2810857
**170Er**	CD3	Polyclonal	10	Fluidigm	3170019D	AB_2811048
**171Yb**	pERK1/2	D13.14.4E	20	Fluidigm	3171021D	AB_2927686
**173Yb***	PR	SP2	10	Abcam	ab239793	AB_2927687
**175Lu**	pS6	N7-548	6.67	Fluidigm	3175031D	AB_2864737
**176Yb**	histone H3	D1H2	1.25	Fluidigm	3176023D	AB_2811058
**191/193Ir**	DNA	-	0.08	Fluidigm	201192A	-

* In-house conjugated

Supplementary table 3 Clinical characteristics of patients in the IHC cohort and the gene expression cohort

Characteristic	IHC cohort, n=518 n(%)	Gene expression cohort, n=83 n(%)
**Age (years)**		
<66	270 (52)	31 (37)
≥66	248 (48)	52 (63)
**Body mass index (BMI)**		
<30	288 (56)	50 (60)
≥30	150 (29)	28 (34)
Missing	80 (15)	5 (6)
**Histologic type**		
Endometrioid	429 (83)	62 (75)
Non-endometrioid	89 (17)	21 (25)
**FIGO stage**		
I	390 (75)	59 (71)
II	45 (9)	7 (8)
III	54 (10)	13 (16)
IV	29 (6)	4 (5)
**Histologic Grade***		
I	201 (47)	21 (34)
II	157 (37)	21 (34)
III	67 (15)	18 (29)
Missing	4 (1)	2 (3)

* **Endometrioid tumors only**

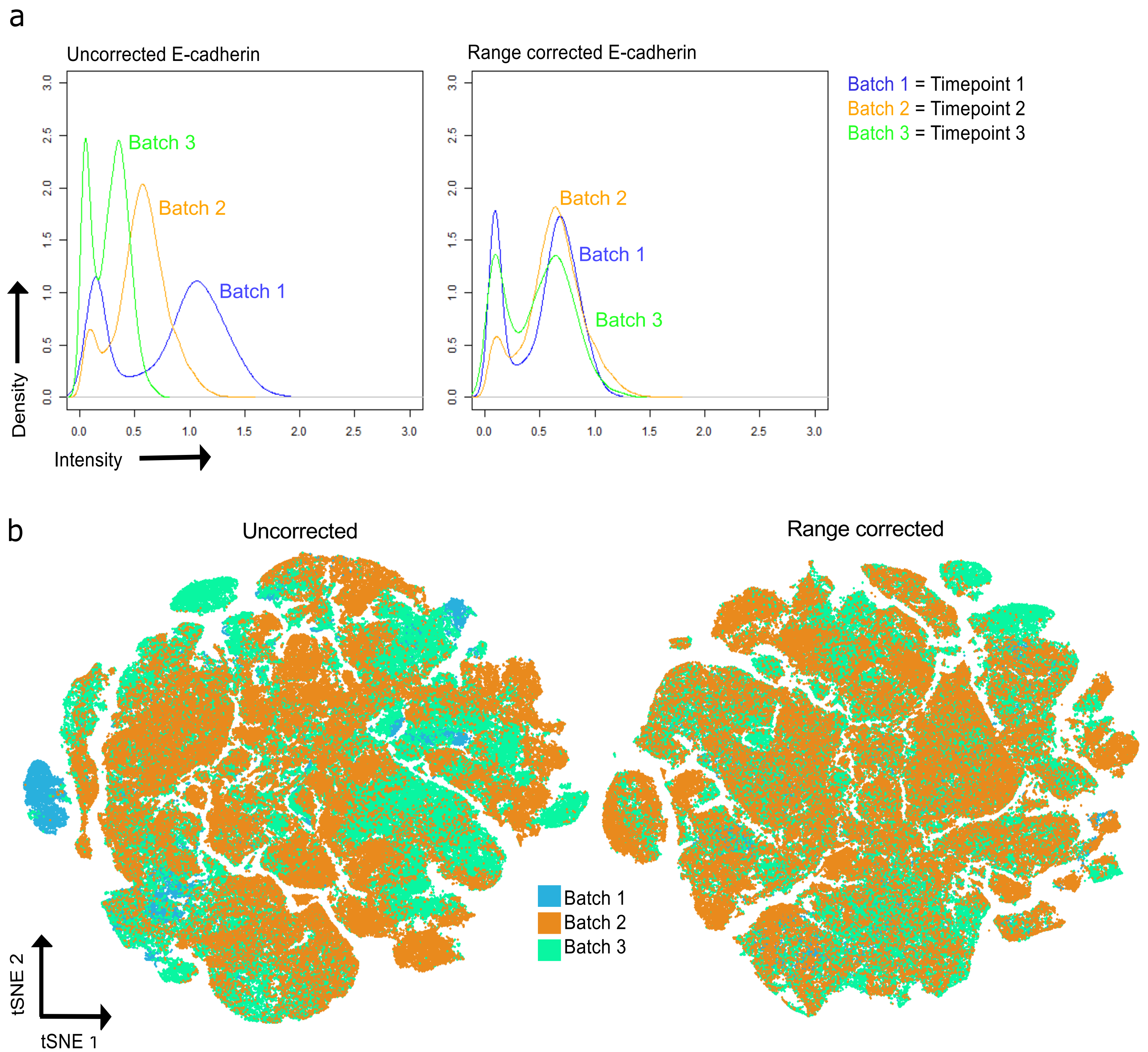

a
Uncorrected E-cadherin
Range corrected E-cadherin
Batch 1 = Timepoint 1
Batch 2 = Timepoint 2
Batch 3 = Timepoint 3
Batch 3
Batch 2
Batch 1
Density
Intensity
0.0
0.5
1.0
1.5
2.0
2.5
3.0
b
Uncorrected
Range corrected
Batch 1
Batch 2
Batch 3
tSNE 2
tSNE 1

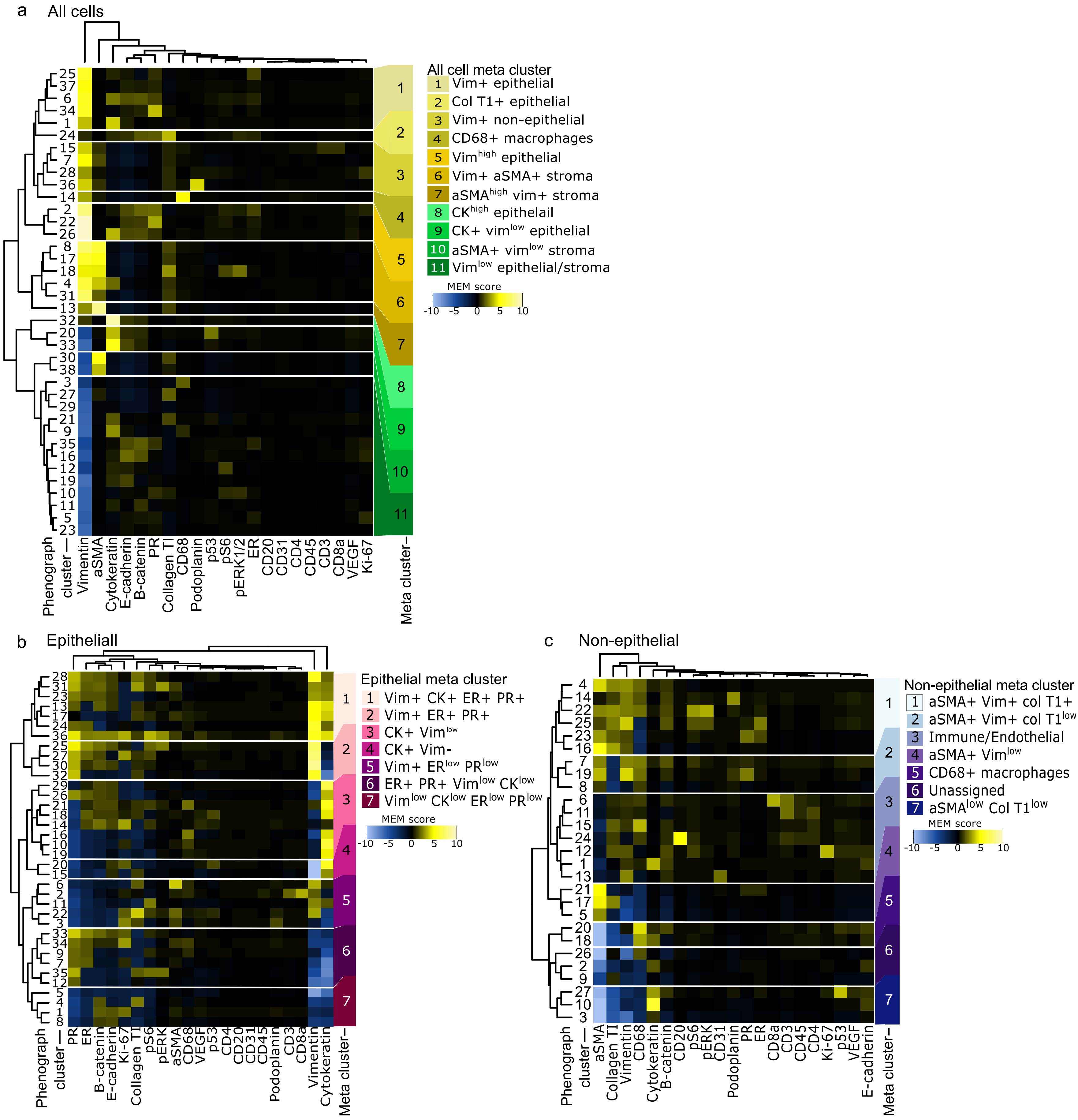

a
All cells
All cell meta cluster
1 Vim+ epithelial
2 Col T1+ epithelial
3 Vim+ non-epithelial
4 CD68+ macrophages
5 Vimhigh epithelial
6 Vim+ aSMA+ stroma
7 aSMAhigh vim+ stroma
8 CKhigh epithelail
9 CK+ vimlow epithelial
10 aSMA+ vimlow stroma
11 Vimlow epithelial/stroma
MEM score
-10 -5 0 5 10
Phenograph cluster
Vimentin
aSMA
Cytokeratin
E-cadherin
B-catenin
PR
Collagen TI
CD68
Podoplanin
p53
pS6
pERK1/2
ER
CD20
CD31
CD4
CD45
CD3
CD8a
VEGF
Ki-67
Meta cluster
b
Epitheliall
Epithelial meta cluster
1 Vim+ CK+ ER+ PR+
2 Vim+ ER+ PR+
3 CK+ Vimlow
4 CK+ Vim-
5 Vim+ ERlow PRlow
6 ER+ PR+ Vimlow CKlow
7 Vimlow CKlow ERlow PRlow
MEM score
-10 -5 0 5 10
Phenograph cluster
PR
ER
B-catenin
E-cadherin
Ki-67
Collagen TI
pS6
pERK
aSMA
CD68
VEGF
p53
CD4
CD20
CD31
CD45
Podoplanin
CD3
CD8a
Vimentin
Cytokeratin
Meta cluster
c
Non-epithelial
Non-epithelial meta cluster
1 aSMA+ Vim+ col T1+
2 aSMA+ Vim+ col T1low
3 Immune/Endothelial
4 aSMA+ Vimlow
5 CD68+ macrophages
6 Unassigned
7 aSMAlow Col T1low
MEM score
-10 -5 0 5 10
Phenograph cluster
aSMA
Collagen TI
Vimentin
CD68
Cytokeratin
B-catenin
CD20
pS6
pERK
CD31
Podoplanin
PR
ER
CD8a
CD3
CD45
CD4
Ki-67
p53
VEGF
E-cadherin
Meta cluster
