# Supplementary material for: Single-cell profiling of low-stage endometrial cancers identifies low epithelial vimentin expression as a marker of recurrent disease
Source: eBioMedicine. 2023 May 3;92:104595. doi: 10.1016/j.ebiom.2023.104595 (PMC10277918; doi:10.1016/j.ebiom.2023.104595)
Supplement: Combined-datasheets [file mmc2.pdf]

Anti-Alpha-Smooth Muscle Actin (1A4)-141Pr

Pathologist-Verified Clone for Imaging Mass Cytometry™

Catalog number: 3141017D
Package size and concentration: 25 µg, 0.5 mg/mL
Clone: 1A4
Isotype: Mouse IgG2a
Pathologist-verified on: Human FFPE, Human Frozen
Fluidigm tested on: Human FFPE, Human Frozen, Mouse FFPE
Reported reactivity: Human, Mouse
Formulation: Antibody stabilizer with 0.05% sodium azide
Storage: Store at 4 °C. Do not freeze.
Application: IMC paraffin, IMC frozen

Technical Information

Description: Alpha-smooth muscle actin is a 42 kDa cytoskeletal protein expressed in smooth muscle cells of blood vessels, myofibroblasts, and myoepithelial cells. It also is found in tumors of smooth muscle and myoepithelial origin.

Application: The metal-tagged antibody is designed and formulated for the application of Imaging Mass Cytometry™ (IMC™) using the Fluidigm Hyperion™ Imaging System on formalin-fixed, paraffin-embedded (FFPE) tissue sections, and frozen tissue sections.

Quality control: Each lot of conjugated antibody is quality control- tested by Imaging Mass Cytometry on tissue sections

Recommended concentration: For optimal performance it is recommended that the antibody be titrated for the desired application. Suggested initial dilution range:
IMC-Paraffin: 1:100 to 1:400
IMC-Frozen: 1:50 to 1:200

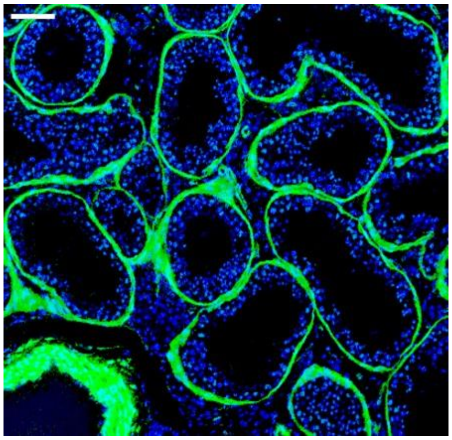

Human testis (FFPE) stained with 141Pr-anti-aSMA (1A4) at a dilution of 1:200 (green pseudocolor) and iridium DNA intercalator (blue pseudocolor). Heat-mediated antigen retrieval was performed using Tris/EDTA buffer pH 9.
Scale bar size = 100 µm.

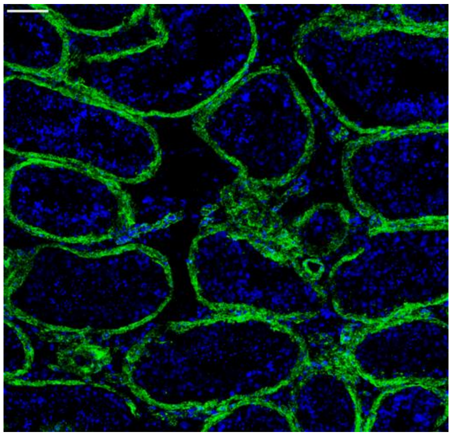

Human frozen testis stained with 141Pr-anti-aSMA (1A4) at a dilution of 1:400 (green pseudocolor) and iridium DNA intercalator (blue pseudocolor). Tissue section was fixed in 4% paraformaldehyde for 30 minutes at 4 °C.
Scale bar size = 100 µm.

References

Chang, Q. et al. "Staining of frozen and formalin-fixed, paraffin-embedded tissues with metal-labeled antibodies for Imaging Mass Cytometry analysis." Current Protocols in Cytometry 82 (2017): 12.47.1–12.47.8.

Giesen, C. et al. "Highly multiplexed imaging of tumor tissues with subcellular resolution by mass cytometry." Nature Methods 11 (2014): 417–22.

Safety

Use standard laboratory safety protocols. Read and understand the safety data sheets (SDSs) before handling chemicals. To obtain SDSs, go to fluidigm.com/sds and search for the SDS using either the product name or the part number.

For technical support visit techsupport.fluidigm.com. | For general support visit fluidigm.com/support.

For Research Use Only. Not for use in diagnostic procedures.

This product contains antibodies manufactured by and sold under license from BioLegend® and licensees thereof.

Information in this publication is subject to change without notice. **Limited Use Label License:** The purchase of this Fluidigm Instrument and/or Consumable product conveys to the purchaser the limited, nontransferable right to use with only Fluidigm Consumables and/or Instruments respectively except as approved in writing by Fluidigm. **Patent and license information:** fluidigm.com/legal/notices. **Trademarks:** Fluidigm, the Fluidigm logo, Hyperion, Imaging Mass Cytometry, and IMC are trademarks and/or registered trademarks of Fluidigm Corporation in the United States and/or other countries. All other trademarks are the sole property of their respective owners. © 2020 Fluidigm Corporation. All rights reserved. 24-Apr-2020

Anti-Vimentin (D21H3)-143Nd

Pathologist-Verified Clone for Imaging Mass Cytometry™

Catalog number: 3143027D
Package size and concentration: 25 µg, 0.5 mg/mL
Clone: D21H3
Isotype: Rabbit IgG
Pathologist-verified on: Human FFPE
Fluidigm tested on: Human FFPE, Mouse FFPE

Reported reactivity: Rat, Mouse, Human, Monkey
Formulation: Antibody stabilizer with 0.05% sodium azide
Storage: Store at 4 °C. Do not freeze.
Application: IMC paraffin

Technical Information

Description: Vimentins are type III intermediate filaments found in various non-epithelial cells, especially mesenchymal cells. They are highly expressed in fibroblasts, with low expression in T and B lymphocytes and little or no expression in Burkitt's lymphoma cell lines. They are also expressed in many hormone-independent mammary carcinoma cell lines. Dynamic structural changes and spatial reorganization of vimentin in response to extracellular stimuli help to coordinate various signaling pathways. Remodeling of vimentin and other intermediate filaments is important during lymphocyte adhesion and migration through the endothelium.

Application: The metal-tagged antibody is designed and formulated for the application of Imaging Mass Cytometry™ (IMC™) using the Fluidigm Hyperion™ Imaging System on formalin-fixed, paraffin-embedded (FFPE) tissue sections.

Quality control: Each lot of conjugated antibody is quality control- tested by Imaging Mass Cytometry on tissue sections

Recommended concentration: For optimal performance it is recommended that the antibody be titrated for the desired application. Suggested initial dilution range:
IMC-Paraffin: 1:50 to 1:200

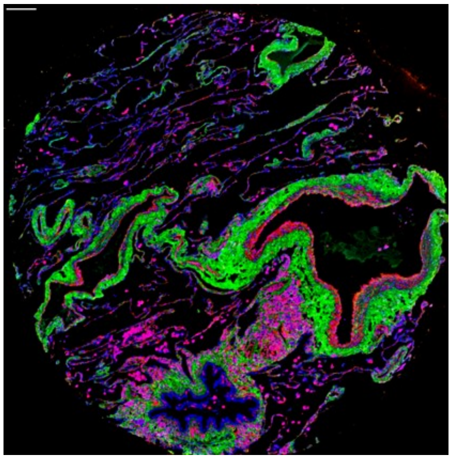

Human lung (FFPE) stained with 143Nd-anti-vimentin (D21H3) at a dilution of 1:100 (red pseudocolor), 169Tmanti-collagen I (poly) (green pseudocolor), and iridium DNA intercalator (blue pseudocolor). Heat-mediated antigen retrieval was performed using Tris/EDTA buffer pH 9. Scale bar size = 100 µm.

References

Chang, Q. et al. "Staining of frozen and formalin-fixed, paraffin-embedded tissues with metal-labeled antibodies for Imaging Mass Cytometry analysis." Current Protocols in Cytometry 82 (2017): 12.47.1–12.47.8.

Giesen, C. et al. "Highly multiplexed imaging of tumor tissues with subcellular resolution by mass cytometry." Nature Methods 11 (2014): 417–22.

Safety

Use standard laboratory safety protocols. Read and understand the safety data sheets (SDSs) before handling chemicals. To obtain SDSs, go to fluidigm.com/sds and search for the SDS using either the product name or the part number.

For Research Use Only. Not for use in diagnostic procedures.

This product contains antibodies manufactured by and sold under license from CST™ and licensees thereof.

Information in this publication is subject to change without notice. **Limited Use Label License:** The purchase of this Fluidigm Instrument and/or Consumable product conveys to the purchaser the limited, nontransferable right to use with only Fluidigm Consumables and/or Instruments respectively except as approved in writing by Fluidigm. **Patent and license information:** fluidigm.com/legal/notices. **Trademarks:** Fluidigm, the Fluidigm logo, Hyperion, Imaging Mass Cytometry, and IMC are trademarks and/or registered trademarks of Fluidigm Corporation in the United States and/or other countries. All other trademarks are the sole property of their respective owners. © 2020 Fluidigm Corporation. All rights reserved. 04-2020

Anti-Pan-Cytokeratin (AE1/AE3)-148Nd

Pathologist-Verified Clone for Imaging Mass Cytometry™

Catalog number: 3148022D
Package size and concentration: 25 µg, 0.5 mg/mL
Clone: AE-1/AE-3
Isotype: Mouse IgG1
Pathologist-verified on: Human FFPE, Human Frozen
Fluidigm tested on: Human FFPE, Human Frozen, Mouse FFPE

Reported reactivity: Human, Mouse, Dog, Primate, Rat
Formulation: Antibody stabilizer with 0.05% sodium azide
Storage: Store at 4 °C. Do not freeze.
Application: IMC paraffin, IMC frozen

Technical Information

Description: AE1/AE3 is a broad-spectrum anti-pan-cytokeratin antibody cocktail that differentiates epithelial tumors from non-epithelial tumors. AE1 immunoreacts with an antigenic determinant present on most of the subfamily A cytokeratins, including cytokeratins with molecular weights of 56.5, 50, 48, and 40 kDa. Antibody AE3 reacts with an antigenic determinant shared by the subfamily B cytokeratins including cytokeratins with molecular weights of 64, 59, 58, 56, and 52 kDa. This antibody stains cytokeratins present in normal and abnormal human tissues and has shown high sensitivity in the recognition of epithelial cells and carcinomas.

Application: The metal-tagged antibody is designed and formulated for the application of Imaging Mass Cytometry™ (IMC™) using the Fluidigm Hyperion™ Imaging System on formalin-fixed, paraffin-embedded (FFPE) tissue sections, and frozen tissue sections.

Quality control: Each lot of conjugated antibody is quality control- tested by Imaging Mass Cytometry on tissue sections

Recommended concentration: For optimal performance it is recommended that the antibody be titrated for the desired application. Suggested initial dilution range:

IMC-Paraffin: 1:50 to 1:200

IMC-Frozen: 1:50 to 1:200

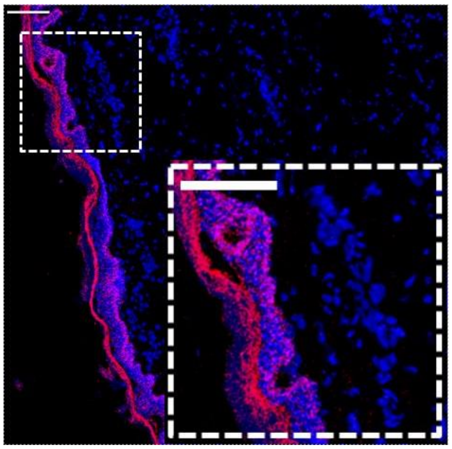

Human frozen skin stained with 148Nd-anti-pan-cytokeratin (AE1/AE3) at a dilution of 1:100 (red pseudocolor) and iridium DNA intercalator (blue pseudocolor). Tissue section was fixed in 4% paraformaldehyde for 30 minutes at 4 °C.

Scale bar size = 100 µm.

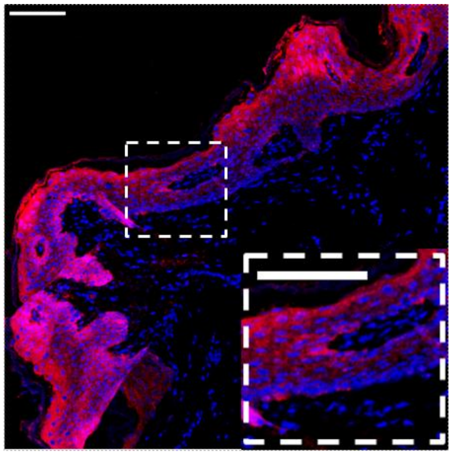

Human skin (FFPE) stained with 148Nd-anti-pan-cytokeratin (AE1/AE3) at a dilution of 1:200 (red pseudocolor) and iridium DNA intercalator (blue pseudocolor). Heat-mediated antigen retrieval was performed using Tris/EDTA buffer pH 9.

Scale bar size = 100 µm.

References

Chang, Q. et al. "Staining of frozen and formalin-fixed, paraffin-embedded tissues with metal-labeled antibodies for Imaging Mass Cytometry analysis." Current Protocols in Cytometry 82 (2017): 12.47.1–12.47.8.

Giesen, C. et al. "Highly multiplexed imaging of tumor tissues with subcellular resolution by mass cytometry." Nature Methods 11 (2014): 417–22.

Safety

Use standard laboratory safety protocols. Read and understand the safety data sheets (SDSs) before handling chemicals. To obtain SDSs, go to fluidigm.com/sds and search for the SDS using either the product name or the part number.

For Research Use Only. Not for use in diagnostic procedures.

This product contains antibodies manufactured by and sold under license from BioLegend® and licensees thereof.

Information in this publication is subject to change without notice. **Limited Use Label License:** The purchase of this Fluidigm Instrument and/or Consumable product conveys to the purchaser the limited, nontransferable right to use with only Fluidigm Consumables and/or Instruments respectively except as approved in writing by Fluidigm. **Patent and license information:** fluidigm.com/legal/notices. **Trademarks:** Fluidigm, the Fluidigm logo, Hyperion, Imaging Mass Cytometry, and IMC are trademarks and/or registered trademarks of Fluidigm Corporation in the United States and/or other countries. All other trademarks are the sole property of their respective owners. © 2020 Fluidigm Corporation. All rights reserved. 24-Apr-2020

Anti-Human CD31/PECAM-1-151Eu

Pathologist-Verified Clone for Imaging Mass Cytometry™

Catalog: 3151025D
Package size and concentration: 25 µg, 0.5 mg/mL
Storage: Store at 4 °C. Do not freeze.
Reactivity: Human

Clone: EPR3094
Isotype: Rabbit IgG
Formulation: Antibody stabilizer with 0.05% sodium azide
Application: IMC-Paraffin

Technical Information

Application: The metal-tagged antibody is designed and formulated for the application of Imaging Mass Cytometry (IMC™) using the Fluidigm Hyperion™ Imaging System on formalin-fixed, paraffin-embedded (FFPE) tissue sections.

Quality control: Each lot of conjugated antibody is quality control-tested by Imaging Mass Cytometry on tissue sections.

Recommended concentration: For optimal performance it is recommended that the antibody be titrated for the desired application. Suggested initial dilution range:
IMC-Paraffin: 1:50 to 1:200

Description

CD31, also known as platelet endothelial cell adhesion molecule-1 (PECAM-1) or endoCAM, is a type I transmembrane glycoprotein. It is expressed by endothelial cells on blood vessels, as well as by monocytes, granulocytes, platelets and a small subset of T cells. It plays a role in wound healing, angiogenesis and removal of aged neutrophils and in cellular migration in an inflammatory situation.

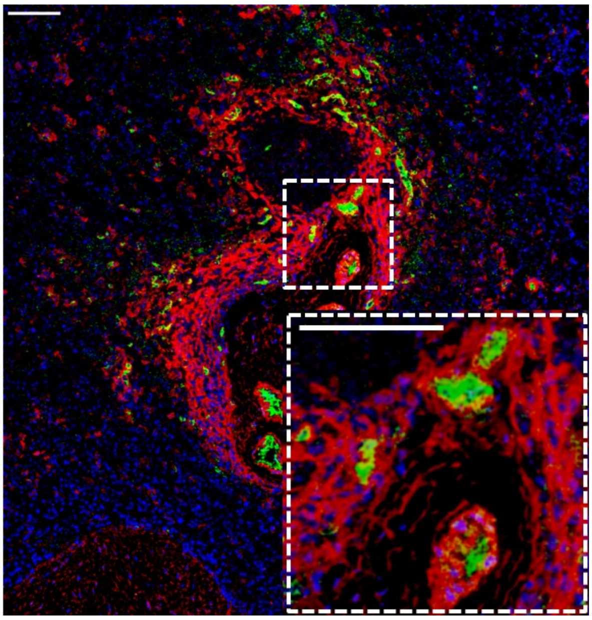

Human spleen (FFPE) stained with 151Eu-anti-CD31 (EPR3094) at a dilution of 1:100 (green pseudocolor), 141Pr-anti-αSMA (1A4) (red pseudocolor), and iridium DNA intercalator (blue pseudocolor). Heat-mediated antigen retrieval was performed using Tris/EDTA buffer pH 9. Scale bar size = 100 µm.

References

Chang, Q. et al. "Staining of frozen and formalin-fixed, paraffin-embedded tissues with metal-labeled antibodies for imaging mass cytometry analysis." *Current Protocols in Cytometry* 82 (2017): 12.47.1–12.47.8.

Giesen, C. et al. "Highly multiplexed imaging of tumor tissues with subcellular resolution by mass cytometry." *Nature Methods* 11 (2014): 417–22.

For Research Use Only. Not for use in diagnostic procedures.

Information in this publication is subject to change without notice. **Safety data sheet information:** www.fluidigm.com/sds. **Patent and license information:** www.fluidigm.com/legalnotices. **Limited Use Label License:** The purchase of this product conveys to the purchaser the limited, non-transferable right to use the purchased consumable or reagent only with Fluidigm Instruments and Systems. **EU's WEEE directive information:** www.fluidigm.com/compliance. Fluidigm, the Fluidigm logo, Hyperion, Imaging Mass Cytometry, and IMC are trademarks and/or registered trademarks of Fluidigm Corporation in the United States and/or other countries. © 2018 Fluidigm Corporation. All rights reserved. 02/2018

abcam

Product datasheet

Anti-Estrogen Receptor alpha antibody [SP1] - BSA and Azide free ab187260

Recombinant RabMAb

14 Images

Overview

**Product name**	Anti-Estrogen Receptor alpha antibody [SP1] - BSA and Azide free
**Description**	Rabbit monoclonal [SP1] to Estrogen Receptor alpha - BSA and Azide free
**Host species**	Rabbit
**Tested applications**	**Suitable for:** mIHC, ICC/IF, Flow Cyt (Intra), WB, IHC-P
**Species reactivity**	**Reacts with:** Human<br>**Predicted to work with:** Mouse, Pig
**Immunogen**	Synthetic peptide. This information is proprietary to Abcam and/or its suppliers.
**Epitope**	C-terminal
**Positive control**	WB: MCF7 cell lysate. IHC-P: Human breast carcinoma, cervix, breast, breast ductal carcinoma and ovarian adenocarcinoma tissue. ICC/IF: MCF7 cells. Flow Cyt (intra): MCF7 cells. mIHC: Human triple-positive breast carcinoma, Human mammary gland tissue sections
**General notes**	ab187260 is the carrier-free version of **ab16660**.

Our **carrier-free** antibodies are typically supplied in a PBS-only formulation, purified and free of BSA, sodium azide and glycerol. The carrier-free buffer and high concentration allow for increased conjugation efficiency.

This conjugation-ready format is designed for use with fluorochromes, metal isotopes, oligonucleotides, and enzymes, which makes them ideal for antibody labelling, functional and cell-based assays, flow-based assays (e.g. mass cytometry) and Multiplex Imaging applications.

Use our **conjugation kits** for antibody conjugates that are ready-to-use in as little as 20 minutes with <1 minute hands-on-time and 100% antibody recovery: available for fluorescent dyes, HRP, biotin and gold.

This product is compatible with the Maxpar® Antibody Labeling Kit from Fluidigm, without the need for antibody preparation. Maxpar® is a trademark of Fluidigm Canada Inc.

This product is a recombinant monoclonal antibody, which offers several advantages including:

- High batch-to-batch consistency and reproducibility
- Improved sensitivity and specificity
- Long-term security of supply
- Animal-free production

For more information **see here**.

This product is FOR RESEARCH USE ONLY. For commercial use, please contact partnerships@abcam.com.

Properties

**Form**	Liquid
**Storage instructions**	Shipped at 4°C. Store at +4°C. Do Not Freeze.
**Storage buffer**	pH: 7.20<br>Constituent: PBS
**Carrier free**	Yes
**Purity**	Affinity purified
**Clonality**	Monoclonal
**Clone number**	SP1
**Isotype**	IgG

Applications

The Abpromise guarantee Our **Abpromise guarantee** covers the use of ab187260 in the following tested applications.

The application notes include recommended starting dilutions; optimal dilutions/concentrations should be determined by the end user.

Application	Abreviews	Notes
**mIHC**		1/200.
**ICC/IF**		Use at an assay dependent concentration.
**Flow Cyt (Intra)**		Use at an assay dependent concentration.
**WB**		Use at an assay dependent concentration. Predicted molecular weight: 67 kDa.
**IHC-P**		Use at an assay dependent concentration.

Target

**Function**	Nuclear hormone receptor. The steroid hormones and their receptors are involved in the regulation of eukaryotic gene expression and affect cellular proliferation and differentiation in target tissues. Can activate the transcriptional activity of TFF1.
**Sequence similarities**	Belongs to the nuclear hormone receptor family. NR3 subfamily.<br>Contains 1 nuclear receptor DNA-binding domain.
**Domain**	Composed of three domains: a modulating N-terminal domain, a DNA-binding domain and a C-terminal ligand-binding domain.
**Post-translational**	Phosphorylated by cyclin A/CDK2. Phosphorylation probably enhances transcriptional activity.<br>Glycosylated; contains N-acetylglucosamine, probably O-linked.

modifications

Ubiquitinated. Deubiquitinated by OTUB1.
Dimethylated by PRMT1 at Arg-260. The methylation may favor cytoplasmic localization.
Palmitoylated (isoform 3). Not biotinylated (isoform 3).

Cellular localization

Nucleus. Cytoplasm. Cell membrane. A minor fraction is associated with the inner membrane and
Nucleus. Cytoplasm. Cell membrane. Associated with the inner membrane via palmitoylation.

Images

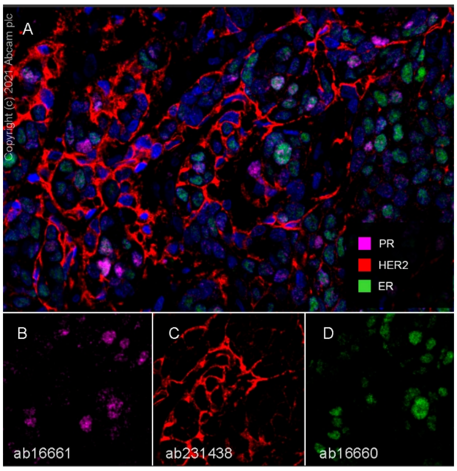

Multiplex immunohistochemistry - Anti-Estrogen Receptor alpha antibody [SP1] - BSA and Azide free (ab187260)

This data was developed using **ab16660**, the same antibody clone in a different buffer formulation.

Multiplex immunohistochemistry (Formalin/PFA-fixed paraffin-embedded sections) analysis of Human triple-positive breast carcinoma tissue sections labeling Estrogen Receptor (ER) with **ab16660**, at a 1/200 dilution (0.07 µg/ml). Heat mediated antigen retrieval with Citrate buffer (pH 6.0, epitope retrieval solution 1) for 20 mins and Opal Polymer HRP Ms + Rb was used as the secondary antibody. DAPI was used as the nuclear counterstain.

Panel A: merged staining of anti-Progesterone Receptor (PR) (magenta; Opal™690), anti-HER2 (red; Opal™570) and anti-Estrogen Receptor (ER) (green; Opal™520) on human triple-positive breast carcinoma.
Panel B: anti-PR stained on nucleus of cancer cells.
Panel C: anti-HER2 stained on membrane of cancer cells.
Panel D: anti-ER stained on nucleus of cancer cells.

The section was incubated in three rounds of staining: in the order of **ab16661** for 30 mins, then **ab16660** and **ab231438** for 10 mins at room temperature. Each round was followed by a separate fluorescent tyramide signal amplification system.

The immunostaining was performed on a Leica Biosystems BOND® RX instrument with an Opal™ 4-color kit. Image acquisition was performed with Leica SP8 confocal microscope.

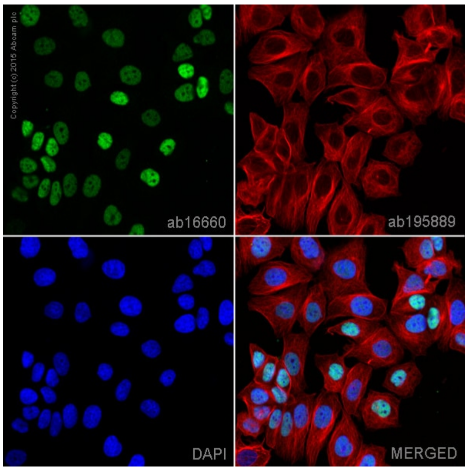

Immunocytochemistry/ Immunofluorescence - Anti-Estrogen Receptor alpha antibody [SP1] - BSA and Azide free (ab187260)

ab16660 staining Estrogen Receptor alpha in MCF7 cells. The cells were fixed with 4% formaldehyde (10min), permeabilized with 0.1% Triton X-100 for 5 minutes and then blocked with 1% BSA/10% normal goat serum/0.3M glycine in 0.1% PBS-Tween for 1h. The cells were then incubated overnight at +4°C with **ab16660** at 1/250 dilution (shown in green) and **ab195889**, Mouse monoclonal [DM1A] to alpha Tubulin (Alexa Fluor® 594), at 2µg/ml (shown in red). Nuclear DNA was labelled with DAPI (shown in blue).
Image was taken with a confocal microscope (Leica-Microsystems, TCS SP8).

This data was developed using the same antibody clone in a different buffer formulation containing PBS, BSA, glycerol, and sodium azide (**ab16660**).

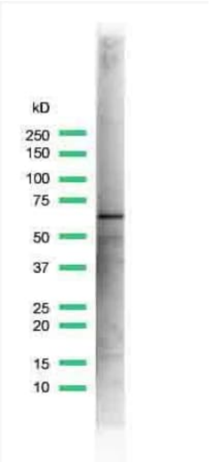

Western blot - Anti-Estrogen Receptor alpha antibody [SP1] - BSA and Azide free (ab187260)

Anti-Estrogen Receptor alpha antibody [SP1] (**ab16660**) at 1/25 dilution + lysate prepared from MCF7 cells

Predicted band size: 67 kDa

This data was developed using **ab16660**, the same antibody clone in a different buffer formulation.

This image was generated using the hybridoma version of the product.

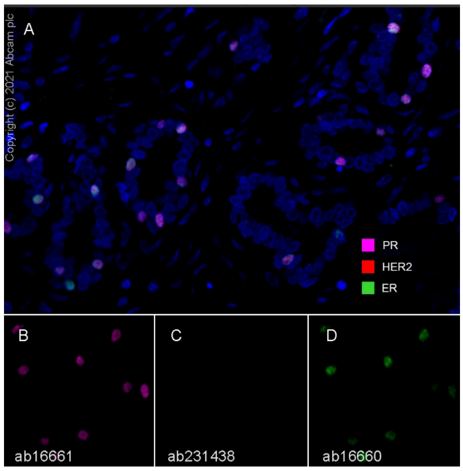

Multiplex immunohistochemistry - Anti-Estrogen Receptor alpha antibody [SP1] - BSA and Azide free (ab187260)

This data was developed using **ab16660**, the same antibody clone in a different buffer formulation.

Multiplex immunohistochemistry (Formalin/PFA-fixed paraffin-embedded sections) analysis of Human mammary gland tissue sections labeling Estrogen Receptor (ER) with **ab16660**, at a 1/200 dilution (0.07 µg/ml). Heat mediated antigen retrieval with Citrate buffer (pH 6.0, epitope retrieval solution 1) for 20 mins and Opal Polymer HRP Ms + Rb was used as the secondary antibody. DAPI was used as the nuclear counterstain.

Panel A: merged staining of anti-Progesterone Receptor (PR) (magenta; Opal™690), anti-HER2 (red; Opal™570) and anti-Estrogen Receptor (ER) (green; Opal™520) on human mammary gland.
Panel B: anti-PR stained on nucleus of some ductal cells.
Panel C: anti-HER2 stained on no cells.
Panel D: anti-ER stained on nucleus of some ductal cells.

The section was incubated in three rounds of staining: in the order of **ab16661** for 30 mins, then **ab16660** and **ab231438** for 10 mins at room temperature. Each round was followed by a separate fluorescent tyramide signal amplification system.

The immunostaining was performed on a Leica Biosystems BOND® RX instrument with an Opal™ 4-color kit. Image acquisition was performed with Leica SP8 confocal microscope.

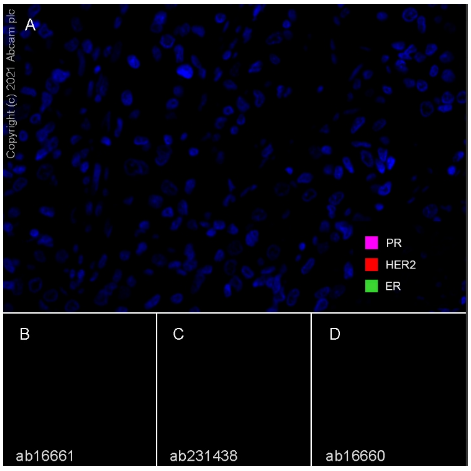

Multiplex immunohistochemistry - Anti-Estrogen Receptor alpha antibody [SP1] - BSA and Azide free (ab187260)

This data was developed using **ab16660**, the same antibody clone in a different buffer formulation.

Multiplex immunohistochemistry (Formalin/PFA-fixed paraffin-embedded sections) analysis of Human triple-negative breast carcinoma tissue sections labeling Estrogen Receptor (ER) with **ab16660**, at a 1/200 dilution (0.07 µg/ml). Heat mediated antigen retrieval with Citrate buffer (pH 6.0, epitope retrieval solution 1) for 20 mins and Opal Polymer HRP Ms + Rb was used as the secondary antibody. DAPI was used as the nuclear counterstain.

Panel A: merged staining of anti-Progesterone Receptor (PR) (magenta; Opal™690), anti-HER2 (red; Opal™570) and anti-Estrogen Receptor (ER) (green; Opal™520) on human triple-negative breast carcinoma.
Panel B: anti-PR stained on no cells.
Panel C: anti-HER2 stained on no cells.
Panel D: anti-ER stained on no cells.

The section was incubated in three rounds of staining: in the order of **ab16661** for 30 mins, then **ab16660** and **ab231438** for 10 mins

at room temperature. Each round was followed by a separate fluorescent tyramide signal amplification system.

The immunostaining was performed on a Leica Biosystems BOND® RX instrument with an Opal™ 4-color kit. Image acquisition was performed with Leica SP8 confocal microscope.

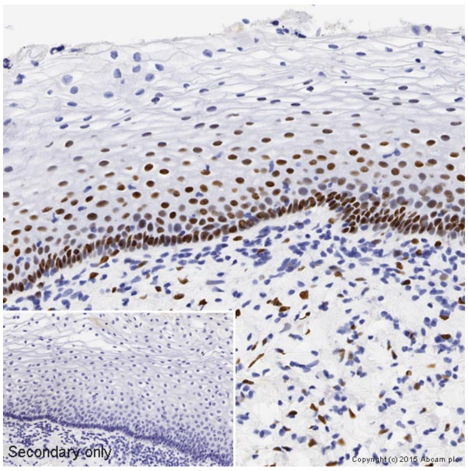

Immunohistochemistry (Formalin/PFA-fixed paraffin-embedded sections) - Anti-Estrogen Receptor alpha antibody [SP1] - BSA and Azide free (ab187260)

IHC image of **ab16660** staining Estrogen Receptor alpha in normal human cervix formalin-fixed paraffin-embedded tissue sections*, performed on a Leica Bond. The section was pre-treated using heat mediated antigen retrieval with sodium citrate buffer (pH6, epitope retrieval solution 1) for 20 mins. The section was then incubated with **ab16660**, 1/250 dilution, for 15 mins at room temperature and detected using an HRP conjugated compact polymer system. DAB was used as the chromogen. The section was then counterstained with haematoxylin and mounted with DPX. No primary antibody was used in the negative control (shown on the inset).

For other IHC staining systems (automated and non-automated) customers should optimize variable parameters such as antigen retrieval conditions, primary antibody concentration and antibody incubation times.

*Tissue obtained from the Human Research Tissue Bank, supported by the NIHR Cambridge Biomedical Research Centre

This data was developed using the same antibody clone in a different buffer formulation containing PBS, BSA, glycerol, and sodium azide (**ab16660**).

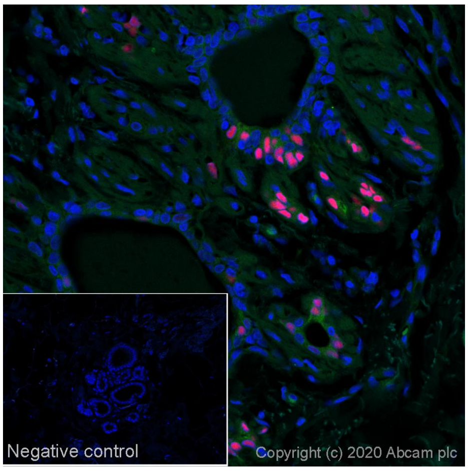

Immunohistochemistry (Formalin/PFA-fixed paraffin-embedded sections) - Anti-Estrogen Receptor alpha antibody [SP1] - BSA and Azide free (ab187260)

Clone SP1 (ab187260) has been successfully conjugated by Abcam. This image was generated using Anti-Estrogen Receptor alpha antibody [SP1] (Alexa Fluor® 647). Please refer to **ab267512** for protocol details.

IHC image of Estrogen Receptor alpha staining in a section of formalin-fixed paraffin-embedded normal human breast*.

The section was pre-treated using heat mediated antigen retrieval with sodium citrate buffer (pH6) in a Biocare Medical NxGen pressure cooker using retrieval settings of 110°C for 20 minutes. Non-specific protein-protein interactions were then blocked in TBS containing 0.025% (v/v) Triton X-100, 0.3M (w/v) glycine and 1% (w/v) BSA for 1h at room temperature. The section was then incubated overnight at +4°C in TBS containing 0.025% (v/v) Triton X-100 and 1% (w/v) BSA with **ab267512** at 1/100 dilution (shown in red) and counterstained using **ab195887**, Mouse monoclonal to alpha Tubulin (Alexa Fluor® 488), at 1/250 dilution (shown in green).

Nuclear DNA was labelled with DAPI (shown in blue). The section was then mounted using Fluoromount®.

Image was taken with a confocal microscope (Leica-Microsystems, TCS SP8).

For other IHC staining systems (automated and non-automated), customers should optimize variable parameters such as antigen retrieval conditions, antibody concentrations and incubation times.

*Tissue obtained from the Human Research Tissue Bank, supported by the NIHR Cambridge Biomedical Research Centre.

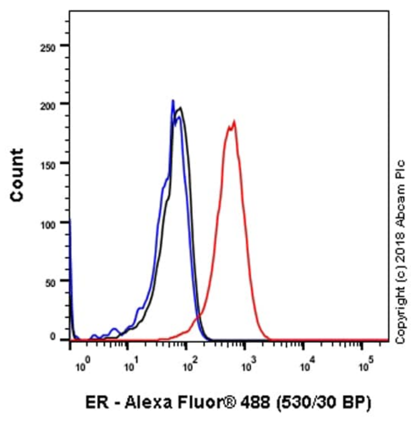

Flow Cytometry (Intracellular) - Anti-Estrogen Receptor alpha antibody [SP1] - BSA and Azide free (ab187260)

Intracellular Flow Cytometry analysis of MCF7 (human breast adenocarcinoma epithelial cell) labeling Estrogen Receptor alpha with purified **ab16660** at 1/200 dilution (1.06µg/ml) (red). Cells were fixed with 4% paraformaldehyde and permeabilised with 90% methanol. Goat anti rabbit IgG (Alexa Fluor®488, **ab150077**) at 1/2000 dilution was used as a secondary antibody. Isotype control - Rabbit monoclonal IgG (**ab172730**) (black). Unlableled control - Unlabelled cells (blue).

This data was developed using the same antibody clone in a different buffer formulation containing PBS, BSA, glycerol, and sodium azide (**ab16660**).

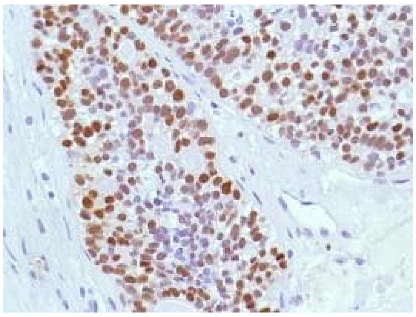

Immunohistochemistry (Formalin/PFA-fixed paraffin-embedded sections) - Anti-Estrogen Receptor alpha antibody [SP1] - BSA and Azide free (ab187260)

Formalin-fixed, paraffin-embedded human ovarian adenocarcinoma tissue stained for Estrogen Receptor alpha using **ab16660** at 1/200 dilution in immunohistochemical analysis.

This data was developed using the same antibody clone in a different buffer formulation containing PBS, BSA, glycerol, and sodium azide (**ab16660**).

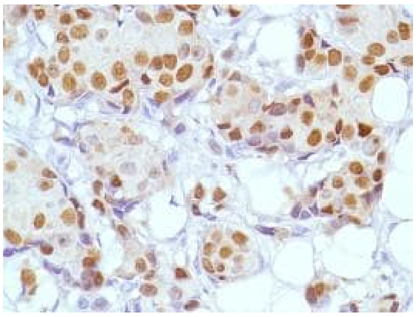

Immunohistochemistry (Formalin/PFA-fixed paraffin-embedded sections) - Anti-Estrogen Receptor alpha antibody [SP1] - BSA and Azide free (ab187260)

Formalin-fixed, paraffin-embedded human breast ductal carcinoma tissue stained for Estrogen Receptor alpha using **ab16660** at 1/200 dilution in immunohistochemical analysis.

This data was developed using the same antibody clone in a different buffer formulation containing PBS, BSA, glycerol, and sodium azide (**ab16660**).

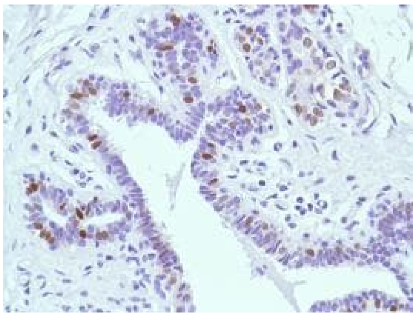

Immunohistochemistry (Formalin/PFA-fixed paraffin-embedded sections) - Anti-Estrogen Receptor alpha antibody [SP1] - BSA and Azide free (ab187260)

Formalin-fixed, paraffin-embedded human breast tissue stained for Estrogen Receptor alpha using **ab16660** at 1/200 dilution in immunohistochemical analysis.

This data was developed using the same antibody clone in a different buffer formulation containing PBS, BSA, glycerol, and sodium azide (**ab16660**).

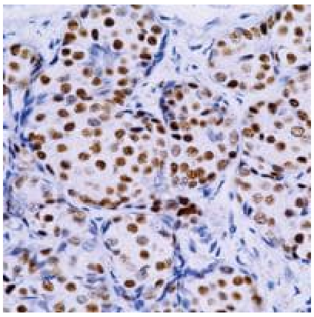

Immunohistochemistry (Formalin/PFA-fixed paraffin-embedded sections) - Anti-Estrogen Receptor alpha antibody [SP1] - BSA and Azide free (ab187260)

Formalin-fixed, paraffin-embedded human breast carcinoma tissue stained for Estrogen Receptor alpha using **ab16660** at 1/200 dilution in immunohistochemical analysis.

This data was developed using the same antibody clone in a different buffer formulation containing PBS, BSA, glycerol, and sodium azide (**ab16660**).

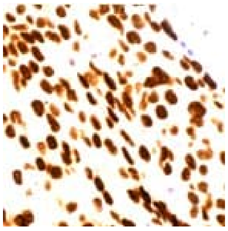

Immunohistochemistry (Formalin/PFA-fixed paraffin-embedded sections) - Anti-Estrogen Receptor alpha antibody [SP1] - BSA and Azide free (ab187260)

Human breast carcinoma stained with **ab16660**.

This data was developed using the same antibody clone in a different buffer formulation containing PBS, BSA, glycerol, and sodium azide (**ab16660**).

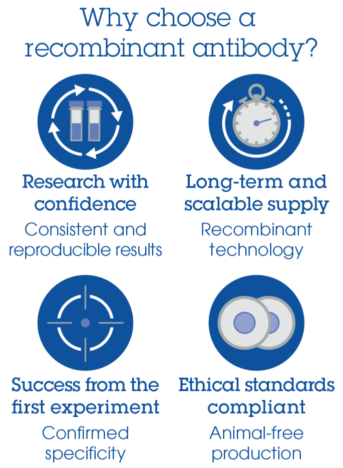

Anti-Estrogen Receptor alpha antibody [SP1] - BSA and Azide free (ab187260)

Please note: All products are "FOR RESEARCH USE ONLY. NOT FOR USE IN DIAGNOSTIC PROCEDURES"

Our Abpromise to you: Quality guaranteed and expert technical support

- Replacement or refund for products not performing as stated on the datasheet
- Valid for 12 months from date of delivery
- Response to your inquiry within 24 hours

- We provide support in Chinese, English, French, German, Japanese and Spanish
- Extensive multi-media technical resources to help you
- We investigate all quality concerns to ensure our products perform to the highest standards

If the product does not perform as described on this datasheet, we will offer a refund or replacement. For full details of the Abpromise, please visit **https://www.abcam.com/abpromise** or contact our technical team.

Terms and conditions

- Guarantee only valid for products bought direct from Abcam or one of our authorized distributors

Anti-CD4 (EPR6855)-156Gd

Pathologist-Verified Clone for Imaging Mass Cytometry

Catalog number: 3156033D
Package size and concentration: 25 µg, 0.5 mg/mL
Clone: EPR6855
Isotype: Rabbit IgG
Pathologist-verified on: Human FFPE
Fluidigm tested on: Human FFPE

Reported reactivity: Human
Formulation: Antibody stabilizer with 0.05% sodium azide
Storage: Store at 4 °C. Do not freeze.
Application: IMC paraffin

Technical Information

Description: CD4, also known as T4, is a transmembrane glycoprotein expressed on the helper subset of T cells (thymocytes) and on immature T cells in the thymus, and weakly on monocytes and dendritic cells. CD4 helps recognize antigens associated with MHC class II molecules via initiation of signal transduction and control of cell-to-cell interaction.

Application: The metal-tagged antibody is designed and formulated for the application of Imaging Mass Cytometry™ (IMC™) using the Fluidigm Hyperion™ Imaging System on formalin-fixed, paraffin-embedded (FFPE) tissue sections.

Quality control: Each lot of conjugated antibody is quality control-tested by Imaging Mass Cytometry on tissue sections.

Recommended concentration: For optimal performance it is recommended that the antibody be titrated for the desired application. Suggested initial dilution range:

IMC paraffin: 1:100 to 1:400

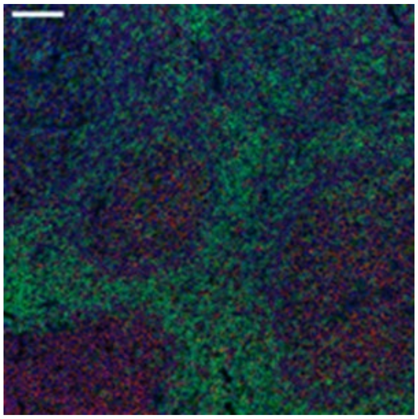

Human tonsil (FFPE) stained with anti-CD4 (ERP6855)-156Gd at a dilution of 1:100 (red pseudocolor), anti-CD20 (H1)-161Dy (green pseudocolor), and iridium DNA intercalator (blue pseudocolor). Heat-mediated antigen retrieval was performed using Tris/EDTA buffer pH 9. Scale bar size = 100 µm.

Safety

Use standard laboratory safety protocols. Read and understand the safety data sheets (SDSs) before handling chemicals. To obtain SDSs, go to fluidigm.com/sds and search for the SDS using either the product name or the part number.

References

Chang, Q. et al. "Staining of frozen and formalin-fixed, paraffin-embedded tissues with metal-labeled antibodies for Imaging Mass Cytometry analysis." Current Protocols in Cytometry 82 (2017): 12.47.1–12.47.8.

Giesen, C. et al. "Highly multiplexed imaging of tumor tissues with subcellular resolution by mass cytometry." Nature Methods 11 (2014): 417–22.

Information in this publication is subject to change without notice. **Limited Use Label License:** The purchase of this Fluidigm Instrument and/or Consumable product conveys to the purchaser the limited, non-transferable right to use with only Fluidigm Consumables and/or Instruments respectively except as approved in writing by Fluidigm: www.fluidigm.com/legal/salesterms. **Patents:** www.fluidigm.com/legal/notices. **Trademarks:** Fluidigm, the Fluidigm logo, Hyperion, Imaging Mass Cytometry, and IMC are trademarks and/or registered trademarks of Fluidigm Corporation or its affiliates in the United States and/or other countries. ©2021 Fluidigm Corporation. All rights reserved. 11/2021

Anti-E-Cadherin (24E10)-158Gd

Pathologist-Verified Clone for Imaging Mass Cytometry™

Catalog number: 3158029D
Package size and concentration: 25 µg, 0.5 mg/mL
Clone: 24E10
Isotype: Rabbit IgG
Pathologist-verified on: Human FFPE, Human Frozen
Fluidigm tested on: Human FFPE, Human Frozen, Mouse FFPE

Reported reactivity: Human, Mouse, Bovine, Cross
Formulation: Antibody stabilizer with 0.05% sodium azide
Storage: Store at 4 °C. Do not freeze.
Application: IMC paraffin, IMC frozen

Technical Information

Description: CD324, also known as epithelial cadherin (E-cadherin) or uvomorulin, is a member of the cadherin superfamily. E-cadherin is a calcium-dependent, transmembrane cell-to-cell adhesion glycoprotein composed of 4 extracellular cadherin repeats and a highly conserved cytoplasmic tail region. It functions as a cell adhesion molecule involved in development, bacterial pathogenesis, and tumor invasion. E-cadherin is widely expressed in epithelial cells in the colon, uterus, liver, keratinocytes, brain, heart, muscle, kidney, and pancreas as well as in erythroid cells.

Application: The metal-tagged antibody is designed and formulated for the application of Imaging Mass Cytometry™ (IMC™) using the Fluidigm Hyperion™ Imaging System on formalin-fixed, paraffin-embedded (FFPE) tissue sections, and frozen tissue sections.

Quality control: Each lot of conjugated antibody is quality control- tested by Imaging Mass Cytometry on tissue sections

Recommended concentration: For optimal performance it is recommended that the antibody be titrated for the desired application. Suggested initial dilution range:
IMC-Paraffin: 1:25 to 1:100
IMC-Frozen: 1:100 to 1:400

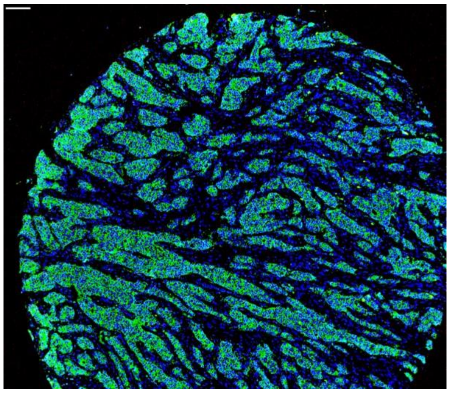

Human breast carcinoma (FFPE) stained with 158Gd-anti-E-cadherin (24E10) at a dilution of 1:50 (green pseudocolor) and iridium DNA intercalator (blue pseudocolor). Heat-mediated antigen retrieval was performed using Tris/EDTA buffer pH 9.
Scale bar size = 100 µm.

References

Chang, Q. et al. "Staining of frozen and formalin-fixed, paraffin-embedded tissues with metal-labeled antibodies for Imaging Mass Cytometry analysis." Current Protocols in Cytometry 82 (2017): 12.47.1–12.47.8.

Giesen, C. et al. "Highly multiplexed imaging of tumor tissues with subcellular resolution by mass cytometry." Nature Methods 11 (2014): 417–22.

Safety

Use standard laboratory safety protocols. Read and understand the safety data sheets (SDSs) before handling chemicals. To obtain SDSs, go to fluidigm.com/sds and search for the SDS using either the product name or the part number.

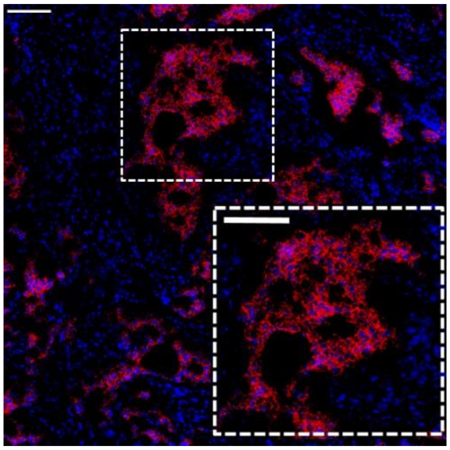

Human frozen breast tumor stained with 158Gd-anti-E-cadherin (24E10) at a dilution of 1:200 (red pseudocolor) and iridium DNA intercalator (blue pseudocolor). Tissue section was fixed in 4% paraformaldehyde for 30 minutes at 4 °C.
Scale bar size = 100 µm.

For technical support visit techsupport.fluidigm.com. | For general support visit fluidigm.com/support.

For Research Use Only. Not for use in diagnostic procedures.

This product contains antibodies manufactured by and sold under license from CST™ and licensees thereof.
Information in this publication is subject to change without notice. **Limited Use Label License:** The purchase of this Fluidigm Instrument and/or Consumable product conveys to the purchaser the limited, nontransferable right to use with only Fluidigm Consumables and/or Instruments respectively except as approved in writing by Fluidigm. **Patent and license information:** fluidigm.com/legal/notices. **Trademarks:** Fluidigm, the Fluidigm logo, Hyperion, Imaging Mass Cytometry, and IMC are trademarks and/or registered trademarks of Fluidigm Corporation in the United States and/or other countries. All other trademarks are the sole property of their respective owners. © 2020 Fluidigm Corporation. All rights reserved. 24-Apr-2020

Anti-Human CD68-159Tb

Pathologist-Verified Clone for Imaging Mass Cytometry

Catalog: 3159035D
Package size and concentration: 25 µg, 0.5 mg/mL
Storage: Store at 4 °C. Do not freeze.
Reactivity: Human

Clone: KP1
Isotype: Mouse IgG1
Formulation: Antibody stabilizer with 0.05% sodium azide
Application: IMC paraffin, IMC frozen

Technical Information

Application: The metal-tagged antibody is designed and formulated for the application of Imaging Mass Cytometry™ (IMC™) using the Fluidigm Hyperion™ Imaging System on formalin-fixed, paraffin-embedded (FFPE) and frozen tissue sections.

Quality control: Each lot of conjugated antibody is quality control-tested by Imaging Mass Cytometry on tissue sections.

Recommended concentration: For optimal performance it is recommended that the antibody be titrated for the desired application. Suggested initial dilution range:
IMC paraffin: 1:25 to 1:100
IMC frozen: 1:200 to 1:800

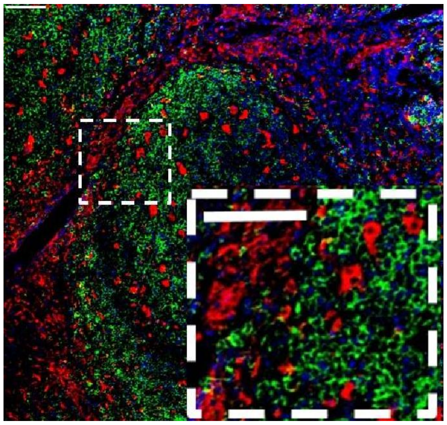

Human tonsil (FFPE) stained with 159Tb-anti-CD68 (KP1) at a dilution of 1:50 (red pseudocolor), 161Dy-anti-CD20 (H1) (green pseudocolor), and iridium DNA intercalator (blue pseudocolor). Heat-mediated antigen retrieval was performed using Tris/EDTA buffer pH 9. Scale bar size = 100 µm.

Description

CD68, also known as macrosialin, is a 110 kDa protein that belongs to the sialomucin family, and it is closely related to the family of highly acidic, highly glycosylated lysosomal-associated membrane proteins (LAMPs). CD68 is predominantly an intracellular protein found in the late endosomal compartment, but it can also be detected in small amounts on the surface of myeloid-derived cells. The function of CD68 is not fully understood, but its structure suggests a role in antigen processing or presentation.

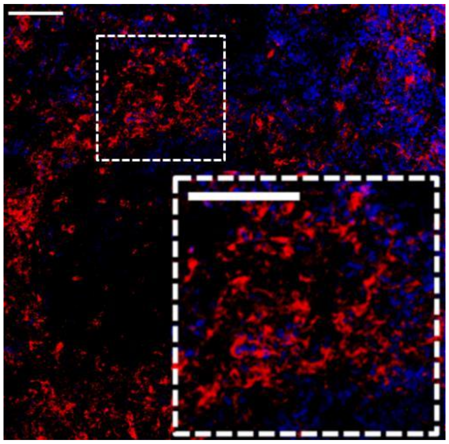

Human frozen tonsil stained with 159Tb-anti-CD68 (KP1) at a dilution of 1:600 (red pseudocolor) and iridium DNA intercalator (blue pseudocolor). Tissue section was fixed in 4% paraformaldehyde for 30 minutes at 4 ºC. Scale bar size = 100 µm.

References

Chang, Q. et al. "Staining of frozen and formalin-fixed, paraffin-embedded tissues with metal-labeled antibodies for Imaging Mass Cytometry analysis." *Current Protocols in Cytometry* 82 (2017): 12.47.1–12.47.8.

Giesen, C. et al. "Highly multiplexed imaging of tumor tissues with subcellular resolution by mass cytometry." *Nature Methods* 11 (2014): 417–22.

For technical support visit techsupport.fluidigm.com. | For general support visit fluidigm.com/support.

For Research Use Only. Not for use in diagnostic procedures.

This product contains antibodies manufactured by and sold under license from BioLegend® and licensees thereof.
Information in this publication is subject to change without notice. **Safety data sheet information:** fluidigm.com/sds. **Patent and license information:** fluidigm.com/legal/notices. **Limited Use Label License:** The purchase of this Fluidigm Instrument and/or Consumable product conveys to the purchaser the limited, nontransferable right to use with only Fluidigm Consumables and/or Instruments respectively except as approved in writing by Fluidigm. **Trademarks:** Fluidigm, the Fluidigm logo, Hyperion, Imaging Mass Cytometry, and IMC are trademarks and/or registered trademarks of Fluidigm Corporation in the United States and/or other countries. All other trademarks are the sole property of their respective owners. © 2019 Fluidigm Corporation. All rights reserved. 11/2019

Revision 4

Store at -20C

#48818

p53 (DO-7) Mouse mAb

Cell Signaling TECHNOLOGY®

Orders: 877-616-CELL (2355) orders@cellsignal.com

Support: 877-678-TECH (8324)

Web: info@cellsignal.com www.cellsignal.com

3 Trask Lane | Danvers | Massachusetts | 01923 | USA

For Research Use Only. Not For Use In Diagnostic Procedures.

Applications:	Reactivity:	Sensitivity:	MW (kDa):	Source/Isotype:	UniProt ID:	Entrez-Gene Id:
WB, IHC-P, IF-IC, FC-FP, ChIP	H	Endogenous	53	Mouse IgG2b	P04637	7157

Product Usage Information

For optimal ChIP results, use 5 µl of antibody and 10 µg of chromatin (approximately 4 x 10^6 cells) per IP. This antibody has been validated using SimpleChIP® Enzymatic Chromatin IP Kits.

Application	Dilution
Western Blotting	1:1000
Immunohistochemistry (Paraffin)	1:100
Immunofluorescence (Immunocytochemistry)	1:400
Flow Cytometry (Fixed/Permeabilized)	1:50
Chromatin IP	1:100

Storage

Supplied in 10 mM sodium HEPES (pH 7.5), 150 mM NaCl, 100 µg/ml BSA, 50% glycerol and less than 0.02% sodium azide. Store at –20°C. Do not aliquot the antibody.

For a carrier-free (BSA and azide free) version of this product see product #46565.

Specificity / Sensitivity

p53 (DO-7) Mouse mAb recognizes endogenous levels of total p53 protein.

Species Reactivity:
Human

Source / Purification

Monoclonal antibody is produced by immunizing animals with recombinant human p53 protein expressed in *E. coli*.

Background

The p53 tumor suppressor protein plays a major role in cellular response to DNA damage and other genomic aberrations. Activation of p53 can lead to either cell cycle arrest and DNA repair or apoptosis (1). p53 is phosphorylated at multiple sites *in vivo* and by several different protein kinases *in vitro* (2,3). DNA damage induces phosphorylation of p53 at Ser15 and Ser20 and leads to a reduced interaction between p53 and its negative regulator, the oncoprotein MDM2 (4). MDM2 inhibits p53 accumulation by targeting it for ubiquitination and proteasomal degradation (5,6). p53 can be phosphorylated by ATM, ATR, and DNA-PK at Ser15 and Ser37. Phosphorylation impairs the ability of MDM2 to bind p53, promoting both the accumulation and activation of p53 in response to DNA damage (4,7). Chk2 and Chk1 can phosphorylate p53 at Ser20, enhancing its tetramerization, stability, and activity (8,9). p53 is phosphorylated at Ser392 *in vivo* (10,11) and by CAK *in vitro* (11). Phosphorylation of p53 at Ser392 is increased in human tumors (12) and has been reported to influence the growth suppressor function, DNA binding, and transcriptional activation of p53 (10,13,14). p53 is phosphorylated at Ser6 and Ser9 by CK1δ and CK1ε both *in vitro* and *in vivo* (13,15). Phosphorylation of p53 at Ser46 regulates the ability of p53 to induce apoptosis (16). Acetylation of p53 is mediated by p300 and CBP acetyltransferases. Inhibition of deacetylation suppressing MDM2 from recruiting HDAC1 complex by p19 (ARF) stabilizes p53. Acetylation appears to play a positive role in the accumulation of p53 protein in stress response (17). Following DNA damage, human p53 becomes acetylated at Lys382 (Lys379 in mouse) *in vivo* to enhance p53-DNA binding (18). Deacetylation of p53 occurs through interaction with the SIRT1 protein, a deacetylase that may be involved in cellular aging and the DNA damage response (19).

1. Levine, A.J. (1997) *Cell* 88, 323-31.
2. Meek, D.W. (1994) *Semin Cancer Biol* 5, 203-10.
3. Milczarek, G.J. et al. (1997) *Life Sci* 60, 1-11.
4. Shieh, S.Y. et al. (1997) *Cell* 91, 325-34.
5. Chehab, N.H. et al. (1999) *Proc Natl Acad Sci U S A* 96, 13777-82.
6. Honda, R. et al. (1997) *FEBS Lett* 420, 25-7.
7. Tibbetts, R.S. et al. (1999) *Genes Dev* 13, 152-7.
8. Shieh, S.Y. et al. (1999) *EMBO J* 18, 1815-23.
9. Hirao, A. et al. (2000) *Science* 287, 1824-7.
10. Hao, M. et al. (1996) *J Biol Chem* 271, 29380-5.
11. Lu, H. et al. (1997) *Mol Cell Biol* 17, 5923-34.
12. Ullrich, S.J. et al. (1993) *Proc Natl Acad Sci U S A* 90, 5954-8.
13. Kohn, K.W. (1999) *Mol Biol Cell* 10, 2703-34.
14. Lohrum, M. and Scheidtmann, K.H. (1996) *Oncogene* 13, 2527-39.
15. Knippschild, U. et al. (1997) *Oncogene* 15, 1727-36.
16. Oda, K. et al. (2000) *Cell* 102, 849-62.
17. Ito, A. et al. (2001) *EMBO J* 20, 1331-40.
18. Sakaguchi, K. et al. (1998) *Genes Dev* 12, 2831-41.
19. Solomon, J.M. et al. (2006) *Mol Cell Biol* 26, 28-38.

Species reactivity is determined by testing in at least one approved application (e.g., western blot).

IMPORTANT: For western blots, incubate membrane with diluted primary antibody in 5% w/v nonfat dry milk, 1X TBS, 0.1% Tween® 20 at 4°C with gentle shaking, overnight.

APPLICATIONS KEY **WB:** Western Blotting **IHC-P:** Immunohistochemistry (Paraffin) **IF-IC:** Immunofluorescence (Immunocytochemistry) **FC-FP:** Flow Cytometry (Fixed/Permeabilized) **ChIP:** Chromatin IP

CROSS-REACTIVITY KEY **H:** human **M:** mouse **R:** rat **Hm:** hamster **Mk:** monkey **Vir:** virus **Mi:** mink **C:** chicken **Dm:** D. melanogaster **X:** Xenopus **Z:** zebrafish **B:** bovine **Dg:** dog **Pg:** pig **Sc:** S. cerevisiae **Ce:** C. elegans **Hr:** horse **GP:** Guinea Pig **Rab:** rabbit **All:** all species expected

Cell Signaling Technology is a trademark of Cell Signaling Technology, Inc.
Alexa Fluor is a registered trademark of Life Technologies Corporation.
All other trademarks are the property of their respective owners. Visit cellsignal.com/trademarks for more information.

Revision 4

#48818

p53 (DO-7) Mouse mAb

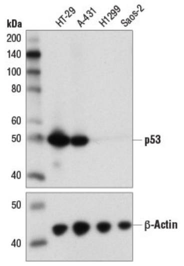

Western blot analysis of extracts from various cell lines using p53 (DO-7) Mouse mAb (upper) and β-Actin (D6A8) Rabbit mAb #8457 (lower).

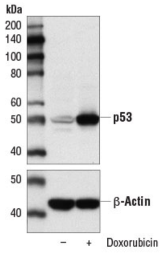

Western blot analysis of extracts from MCF7 cells, untreated (-) or treated with Doxorubicin #5927 (0.5 µM, 24 hr; +), using p53 (DO-7) Mouse mAb (upper) and β-Actin (D6A8) Rabbit mAb #8457 (lower).

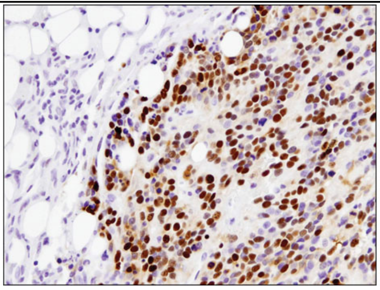

Immunohistochemical analysis of paraffin-embedded human breast carcinoma using p53 (DO-7) Mouse mAb.

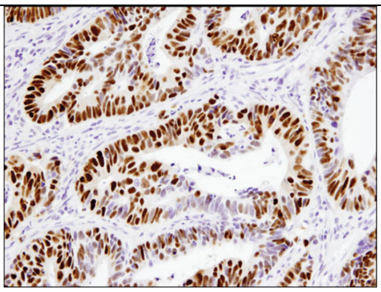

Immunohistochemical analysis of paraffin-embedded human colon carcinoma using p53 (DO-7) Mouse mAb.

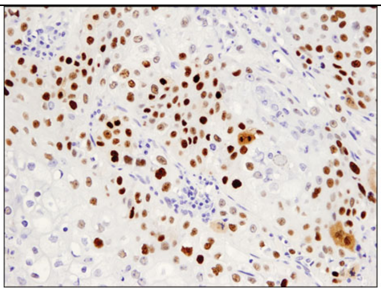

Immunohistochemical analysis of paraffin-embedded human squamous cell lung carcinoma using p53 (DO-7) Mouse mAb.

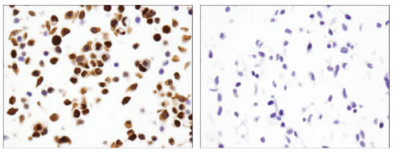

Immunohistochemical analysis of paraffin-embedded HT-29 (left) and Saos-2 (right) cells using p53 (DO-7) Mouse mAb.

Revision 4

#48818

p53 (DO-7) Mouse mAb

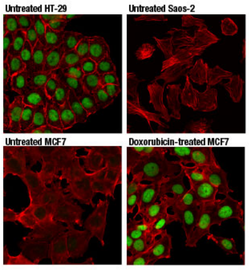

Confocal immunofluorescent analysis of untreated HT-29 cells (top left), untreated Saos-2 cells (top right), untreated MCF7 cells (bottom left), and MCF7 cells treated with Doxorubicin #5927 (0.5 µM, 24 hr; bottom right), using p53 (DO-7) Mouse mAb (green). Actin filaments were labeled with DyLight™ 554 Phalloidin #13054 (red).

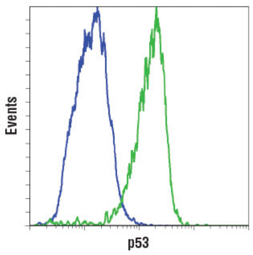

Flow cytometric analysis of H1299 cells (blue) and HT-29 cells (green) using p53 (DO-7) Mouse mAb. Anti-mouse IgG (H+L), $F(ab')_2$ Fragment (Alexa Fluor® 488 Conjugate) #4408 was used as a secondary antibody.

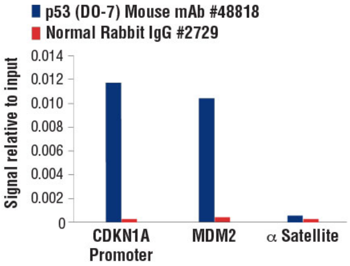

Chromatin immunoprecipitations were performed with cross-linked chromatin from HCT 116 cells treated with UV (100 J/m^2 followed by a 3 hr recovery) and either p53 (DO-7) Mouse mAb or Normal Rabbit IgG #2729 using SimpleChIP® Enzymatic Chromatin IP Kit (Magnetic Beads) #9003. The enriched DNA was quantified by real-time PCR using SimpleChIP® Human CDKN1A Promoter Primers #6449, human MDM2 intron 2 primers, and SimpleChIP® Human α Satellite Repeat Primers #4486. The amount of immunoprecipitated DNA in each sample is represented as signal relative to the total amount of input chromatin, which is equivalent to one.

#48818

p53 (DO-7) Mouse mAb

Limited Uses

Except as otherwise expressly agreed in a writing signed by a legally authorized representative of CST, the following terms apply to Products provided by CST, its affiliates or its distributors. Any Customer's terms and conditions that are in addition to, or different from, those contained herein, unless separately accepted in writing by a legally authorized representative of CST, are rejected and are of no force or effect.

Products are labeled with For Research Use Only or a similar labeling statement and have not been approved, cleared, or licensed by the FDA or other regulatory foreign or domestic entity, for any purpose. Customer shall not use any Product for any diagnostic or therapeutic purpose, or otherwise in any manner that conflicts with its labeling statement. Products sold or licensed by CST are provided for Customer as the end-user and solely for research and development uses. Any use of Product for diagnostic, prophylactic or therapeutic purposes, or any purchase of Product for resale (alone or as a component) or other commercial purpose, requires a separate license from CST. Customer shall (a) not sell, license, loan, donate or otherwise transfer or make available any Product to any third party, whether alone or in combination with other materials, or use the Products to manufacture any commercial products, (b) not copy, modify, reverse engineer, decompile, disassemble or otherwise attempt to discover the underlying structure or technology of the Products, or use the Products for the purpose of developing any products or services that would compete with CST products or services, (c) not alter or remove from the Products any trademarks, trade names, logos, patent or copyright notices or markings, (d) use the Products solely in accordance with CST Product Terms of Sale and any applicable documentation, and (e) comply with any license, terms of service or similar agreement with respect to any third party products or services used by Customer in connection with the Products.

Anti-Human CD20-161Dy

Pathologist-Verified Clone for Imaging Mass Cytometry™

Catalog: 3161029D
Package size and concentration: 25 µg, 0.5 mg/mL
Storage: Store at 4 °C. Do not freeze.
Reactivity: Human

Clone: H1
Isotype: Mouse IgG2a
Formulation: Antibody stabilizer with 0.05% sodium azide
Application: IMC-Paraffin

Technical Information

Application: The metal-tagged antibody is designed and formulated for the application of Imaging Mass Cytometry (IMC™) using the Fluidigm Hyperion™ Imaging System on formalin-fixed, paraffin-embedded (FFPE) tissue sections.

Quality control: Each lot of conjugated antibody is quality control-tested by Imaging Mass Cytometry on tissue sections.

Recommended concentration: For optimal performance it is recommended that the antibody be titrated for the desired application.
Suggested initial dilution range:
IMC-Paraffin: 1:200 to 1:800

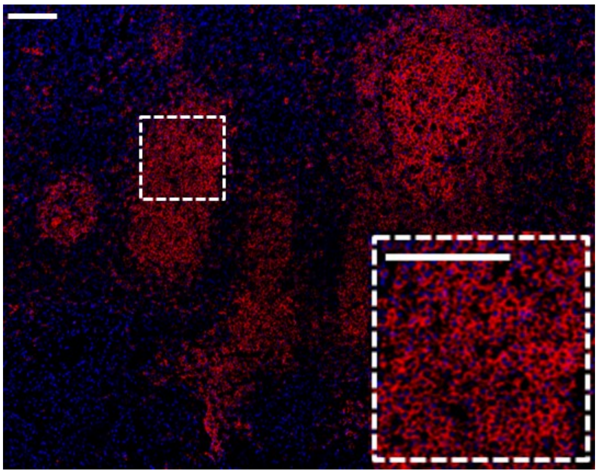

Human tonsil (FFPE) stained with 161Dy-anti-CD20 (H1) at a dilution of 1:400 (red pseudocolor) and iridium DNA intercalator (blue pseudocolor). Heat-mediated antigen retrieval was performed using Tris/EDTA buffer pH 9. Scale bar size = 100 µm.

Description

CD20, also known as B1 and Bp35, is a transmembrane protein. CD20 is expressed on pre-B cells, immature and mature B cells and some follicular dendritic cells. Phosphorylation of CD20 is involved in B cell activation and is associated with various Src family kinases. CD20 can exist in a complex with MHC, CD53, CD81 and CD82.

References

Chang, Q. et al. "Staining of frozen and formalin-fixed, paraffin-embedded tissues with metal-labeled antibodies for imaging mass cytometry analysis." *Current Protocols in Cytometry* 82 (2017): 12.47.1–12.47.8.

Giesen, C. et al. "Highly multiplexed imaging of tumor tissues with subcellular resolution by mass cytometry." *Nature Methods* 11 (2014): 417–22.

For Research Use Only. Not for use in diagnostic procedures.

Information in this publication is subject to change without notice. **Safety data sheet information:** www.fluidigm.com/sds. **Patent and license information:** www.fluidigm.com/legalnotices. **Limited Use Label License:** The purchase of this product conveys to the purchaser the limited, non-transferable right to use the purchased consumable or reagent only with Fluidigm Instruments and Systems. **EU's WEEE directive information:** www.fluidigm.com/compliance. Fluidigm, the Fluidigm logo, Hyperion, Imaging Mass Cytometry, and IMC are trademarks or registered trademarks of Fluidigm Corporation in the United States and/or other countries. © 2017 Fluidigm Corporation. All rights reserved. 10/2017

Anti-Human CD8α-162Dy

Pathologist-Verified Clone for Imaging Mass Cytometry™

Catalog: 3162035D
Package size and concentration: 25 µg, 0.5 mg/mL
Storage: Store at 4 °C. Do not freeze.
Reactivity: Human

Clone: D8A8Y
Isotype: Rabbit IgG
Formulation: Antibody stabilizer with 0.05% sodium azide
Application: IMC-Paraffin

Technical Information

Application: The metal-tagged antibody is designed and formulated for the application of Imaging Mass Cytometry (IMC™) using the Fluidigm Hyperion™ Imaging System on formalin-fixed, paraffin-embedded (FFPE) tissue sections.

Quality control: Each lot of conjugated antibody is quality control-tested by Imaging Mass Cytometry on tissue sections.

Recommended concentration: For optimal performance it is recommended that the antibody be titrated for the desired application. Suggested initial dilution range:
IMC-Paraffin: 1:25 to 1:100

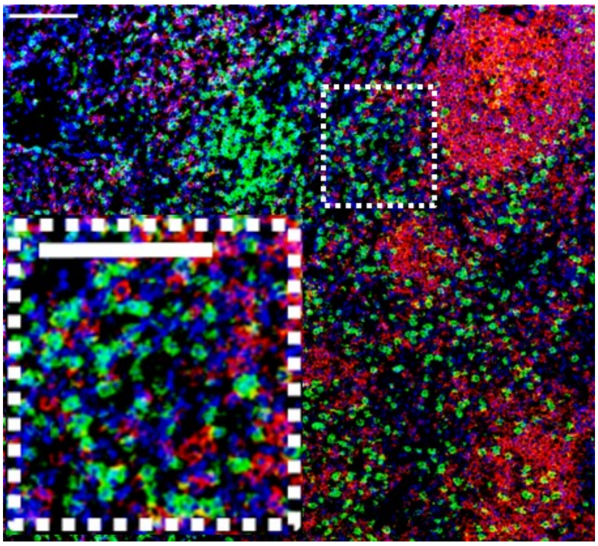

Human tonsil (FFPE) stained with 162Dy-anti-CD8α (D8A8Y) at a dilution of 1:50 (green pseudocolor), 161Dy-anti-CD20 (H1) (red pseudocolor), and iridium DNA intercalator (blue pseudocolor). Heat-mediated antigen retrieval was performed using Tris/EDTA buffer pH 9. Scale bar size = 100 µm.

Description

CD8, also known as T8 and Leu2, is a type I membrane glycoprotein consisting of two disulfide-linked chains (CD8a, CD8b). CD8 is a member of the immunoglobulin superfamily found on the majority of thymocytes, a subset of peripheral blood T cells, and NK cells (which express almost exclusively CD8a homodimers). CD8 acts as a co-receptor with MHC class I-restricted T cell receptors in antigen recognition and T cell activation and has been shown to play a role in thymic differentiation. Two domains in CD8a are important for function: the extracellular IgSF domain binds the α3 domain of MHC class I, and the cytoplasmic CXCP motif binds the tyrosine kinase p56 Lck.

References

Chang, Q. et al. "Staining of frozen and formalin-fixed, paraffin-embedded tissues with metal-labeled antibodies for imaging mass cytometry analysis." *Current Protocols in Cytometry* 82 (2017): 12.47.1–12.47.8.

Giesen, C. et al. "Highly multiplexed imaging of tumor tissues with subcellular resolution by mass cytometry." *Nature Methods* 11 (2014): 417–22.

For technical support visit http://techsupport.fluidigm.com. | For general support visit www.fluidigm.com/support.

For Research Use Only. Not for use in diagnostic procedures.

This product contains antibodies manufactured by and sold under license from CST™ and licensees thereof.
Information in this publication is subject to change without notice. **Safety data sheet information:** www.fluidigm.com/sds. **Patent and license information:** www.fluidigm.com/legalnotices. **Limited Use Label License:** The purchase of this product conveys to the purchaser the limited, non-transferable right to use the purchased consumable or reagent only with Fluidigm Instruments and Systems. **EU's WEEE directive information:** www.fluidigm.com/compliance. Fluidigm, the Fluidigm logo, Hyperion, Imaging Mass Cytometry, and IMC are trademarks or registered trademarks of Fluidigm Corporation in the United States and/or other countries. All other trademarks are the sole property of their respective owners. © 2017 Fluidigm Corporation. All rights reserved. 10/2017

abcam

Product datasheet

Anti-Progesterone Receptor antibody [SP2] - BSA and Azide free ab239793

Recombinant RabMAb

8 Images

Overview

**Product name**	Anti-Progesterone Receptor antibody [SP2] - BSA and Azide free
**Description**	Rabbit monoclonal [SP2] to Progesterone Receptor - BSA and Azide free
**Host species**	Rabbit
**Tested applications**	**Suitable for:** Flow Cyt, mIHC, WB, ICC/IF, IHC-P
**Species reactivity**	**Reacts with:** Human<br>**Predicted to work with:** Rat, Rabbit
**Immunogen**	Recombinant fragment. This information is proprietary to Abcam and/or its suppliers.
**Epitope**	Amino acids 412-526
**Positive control**	Breast carcinomas IHC-P: Human breast carcinoma tissue. ICC/IF: T-47D cells Flow Cyt: T-47D cells mIHC: Human mammary gland tissue sections, Human triple-positive breast carcinoma tissue sections
**General notes**	ab239793 is the carrier-free version of **ab16661**.

Our **carrier-free** antibodies are typically supplied in a PBS-only formulation, purified and free of BSA, sodium azide and glycerol. The carrier-free buffer and high concentration allow for increased conjugation efficiency.

This conjugation-ready format is designed for use with fluorochromes, metal isotopes, oligonucleotides, and enzymes, which makes them ideal for antibody labelling, functional and cell-based assays, flow-based assays (e.g. mass cytometry) and Multiplex Imaging applications.

Use our **conjugation kits** for antibody conjugates that are ready-to-use in as little as 20 minutes with <1 minute hands-on-time and 100% antibody recovery: available for fluorescent dyes, HRP, biotin and gold.

This product is compatible with the Maxpar® Antibody Labeling Kit from Fluidigm, without the need for antibody preparation. Maxpar® is a trademark of Fluidigm Canada Inc.

This product is a recombinant monoclonal antibody, which offers several advantages including:

- High batch-to-batch consistency and reproducibility
- Improved sensitivity and specificity
- Long-term security of supply
- Animal-free production

For more information **see here**.

This product is FOR RESEARCH USE ONLY. For commercial use, please contact partnerships@abcam.com.

Properties

**Form**	Liquid
**Storage instructions**	Shipped at 4°C. Store at +4°C. Do Not Freeze.
**Storage buffer**	pH: 7.20<br>Constituent: PBS
**Carrier free**	Yes
**Purity**	Affinity purified
**Clonality**	Monoclonal
**Clone number**	SP2
**Isotype**	IgG

Applications

The Abpromise guarantee Our **Abpromise guarantee** covers the use of ab239793 in the following tested applications.

The application notes include recommended starting dilutions; optimal dilutions/concentrations should be determined by the end user.

Application	Abreviews	Notes
**Flow Cyt**		Use at an assay dependent concentration.<br>**ab199376** - Rabbit monoclonal IgG, is suitable for use as an isotype control with this antibody.
**mIHC**		1/6000.
**WB**		Use at an assay dependent concentration. Predicted molecular weight: 99120 kDa.
**ICC/IF**		Use at an assay dependent concentration.
**IHC-P**		Use at an assay dependent concentration.<br>Staining of formalin-fixed tissues is required by boiling tissue sections in 10mM citrate buffer, pH 6.0 for 10 min followed by cooling at RT for 20 min.

Target

Function

The steroid hormones and their receptors are involved in the regulation of eukaryotic gene expression and affect cellular proliferation and differentiation in target tissues. Progesterone receptor isoform B (PRB) is involved activation of c-SRC/MAPK signaling on hormone stimulation.
Isoform A: inactive in stimulating c-Src/MAPK signaling on hormone stimulation.

Isoform 4: Increases mitochondrial membrane potential and cellular respiration upon stimulation by progesterone.

Sequence similarities

Belongs to the nuclear hormone receptor family. NR3 subfamily.
Contains 1 nuclear receptor DNA-binding domain.

Domain

Composed of three domains: a modulating N-terminal domain, a DNA-binding domain and a C-terminal ligand-binding domain.

Post-translational modifications

Phosphorylated on multiple serine sites. Several of these sites are hormone-dependent. Phosphorylation on Ser-294 occurs preferentially on isoform B, is highly hormone-dependent and modulates ubiquitination and sumoylation on Lys-388. Phosphorylation on Ser-102 and Ser-345 also requires induction by hormone. Basal phosphorylation on Ser-81, Ser-162, Ser-190 and Ser-400 is increased in response to progesterone and can be phosphorylated in vitro by the CDK2-A1 complex. Increased levels of phosphorylation on Ser-400 also in the presence of EGF, heregulin, IGF, PMA and FBS. Phosphorylation at this site by CDK2 is ligand-independent, and increases nuclear translocation and transcriptional activity. Phosphorylation at Ser-162 and Ser-294, but not at Ser-190, is impaired during the G(2)/M phase of the cell cycle. Phosphorylation on Ser-345 by ERK1/2 MAPK is required for interaction with SP1.
Sumoylation is hormone-dependent and represses transcriptional activity. Sumoylation on all three sites is enhanced by PIAS3. Desumoylated by SENP1. Sumoylation on Lys-388, the main site of sumoylation, is repressed by ubiquitination on the same site, and modulated by phosphorylation at Ser-294.
Ubiquitination is hormone-dependent and represses sumoylation on the same site. Promoted by MAPK-mediated phosphorylation on Ser-294.
Palmitoylated by ZDHHC7 and ZDHHC21. Palmitoylation is required for plasma membrane targeting and for rapid intracellular signaling via ERK and AKT kinases and cAMP generation.

Cellular localization

Nucleus. Cytoplasm. Nucleoplasmic shuttling is both homone- and cell cycle-dependent. On hormone stimulation, retained in the cytoplasm in the G(1) and G(2)/M phases; Mitochondrion outer membrane and Nucleus. Cytoplasm. Mainly nuclear.

Images

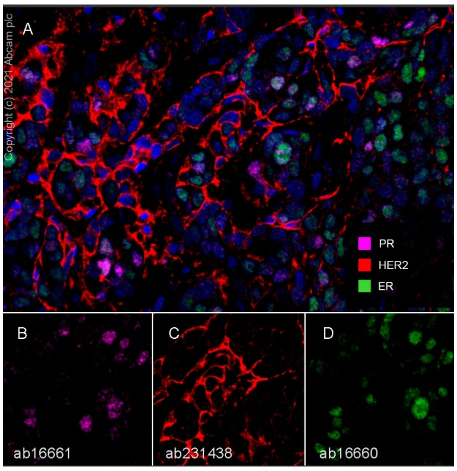

Multiplex immunohistochemistry - Anti-Progesterone Receptor antibody [SP2] - BSA and Azide free (ab239793)

This data was developed using **ab16661**, the same antibody clone in a different buffer formulation.

Multiplex immunohistochemistry (Formalin/PFA-fixed paraffin-embedded sections) analysis of Human triple-positive breast carcinoma tissue sections labeling Progesterone Receptor (PR) with **ab16661**, at a 1/6000 dilution (0.2 µg/ml). Heat mediated antigen retrieval with Citrate buffer (pH 6.0, epitope retrieval solution 1) for 20 mins and Opal Polymer HRP Ms + Rb was used as the secondary antibody. DAPI was used as the nuclear counterstain.

Panel A: merged staining of anti-Progesterone Receptor (PR) (magenta; Opal™690), anti-HER2 (red; Opal™570) and anti-Estrogen Receptor (ER) (green; Opal™520) on human triple-positive breast carcinoma.
Panel B: anti-PR stained on nucleus of cancer cells.
Panel C: anti-HER2 stained on membrane of cancer cells.

Panel D: anti-ER stained on nucleus of cancer cells.

The section was incubated in three rounds of staining: in the order of **ab16661** for 30 mins, then **ab16660** and **ab231438** for 10 mins at room temperature. Each round was followed by a separate fluorescent tyramide signal amplification system.

The immunostaining was performed on a Leica Biosystems BOND® RX instrument with an Opal™ 4-color kit. Image acquisition was performed with Leica SP8 confocal microscope.

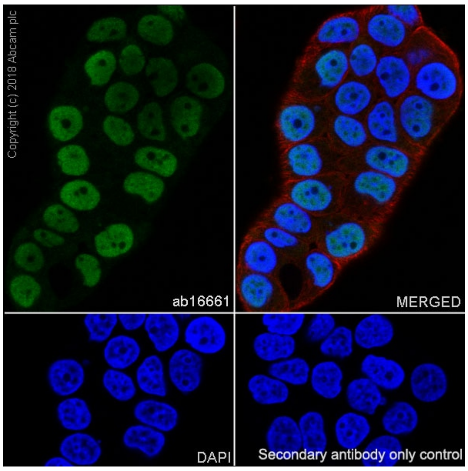

Immunocytochemistry/ Immunofluorescence - Anti-Progesterone Receptor antibody [SP2] - BSA and Azide free (ab239793)

Immunocytochemistry/ Immunofluorescence analysis of T-47D (human ductal breast epithelial tumor epithelial cell) cells labeling Progesterone Receptor with purified **ab16661** at 1:100 (2.28 µg/ml). Cells were fixed in 4% paraformaldehyde and permeabilized with 0.1% Triton X-100. Cells were counterstained with Ab195889 Anti-alpha Tubulin antibody [DM1A] - Microtubule Marker (Alexa Fluor® 594) 1:200 (2.5 µg/ml). Goat anti rabbit IgG (Alexa Fluor® 488, **ab150077**) was used as the secondary antibody at 1:1000 (2 µg/ml) dilution. DAPI nuclear counterstain. PBS instead of the primary antibody was used as the secondary antibody only control.This data was developed using the same antibody clone in a different buffer formulation containing PBS, BSA, glycerol, and sodium azide (ab239793)

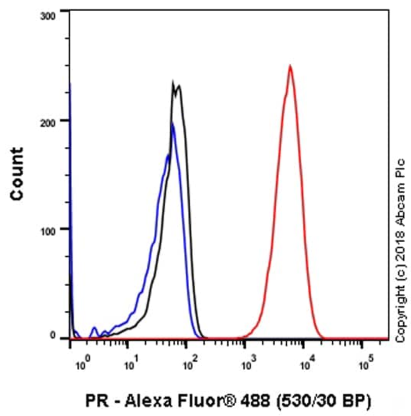

Flow Cytometry - Anti-Progesterone Receptor antibody [SP2] - BSA and Azide free (ab239793)

Flow Cytometry analysis of T-47D (human ductal breast epithelial tumor epithelial cell) cells labeling Progesterone Receptor with purified **ab16661** at 1:220 dilution (1.04 µg/ml) - Red. Cells were fixed with 4% paraformaldehyde . A Goat anti rabbit IgG (Alexa Fluor® 488, **ab150077**) secondary antibody was used at 1:2000 dilution. Isotype control - Rabbit monoclonal IgG (**ab172730**) - Black. Unlabeled control - Blue.This data was developed using the same antibody clone in a different buffer formulation containing PBS, BSA, glycerol, and sodium azide (ab239793)

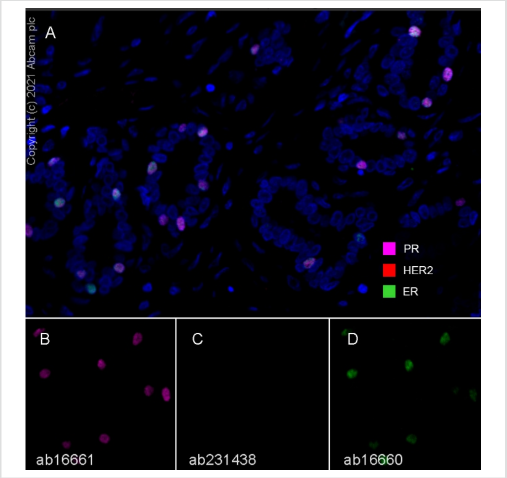

Multiplex immunohistochemistry - Anti-Progesterone Receptor antibody [SP2] - BSA and Azide free (ab239793)

This data was developed using **ab16661**, the same antibody clone in a different buffer formulation.

Multiplex immunohistochemistry (Formalin/PFA-fixed paraffin-embedded sections) analysis of Human mammary gland tissue sections labeling Progesterone Receptor (PR) with **ab16661**, at a 1/6000 dilution (0.2 µg/ml). Heat mediated antigen retrieval with Citrate buffer (pH 6.0, epitope retrieval solution 1) for 20 mins and Opal Polymer HRP Ms + Rb was used as the secondary antibody. DAPI was used as the nuclear counterstain.

Panel A: merged staining of anti-Progesterone Receptor (PR) (magenta; Opal™690), anti-HER2 (red; Opal™570) and anti-Estrogen Receptor (ER) (green; Opal™520) on human mammary gland.
Panel B: anti-PR stained on nucleus of some ductal cells.
Panel C: anti-HER2 stained on no cells.
Panel D: anti-ER stained on nucleus of some ductal cells.

The section was incubated in three rounds of staining: in the order of **ab16661** for 30 mins, then **ab16660** and **ab231438** for 10 mins at room temperature. Each round was followed by a separate fluorescent tyramide signal amplification system.

The immunostaining was performed on a Leica Biosystems BOND® RX instrument with an Opal™ 4-color kit. Image acquisition was performed with Leica SP8 confocal microscope.

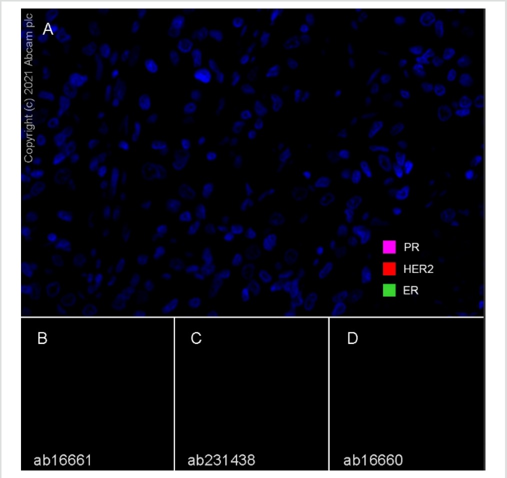

Multiplex immunohistochemistry - Anti-Progesterone Receptor antibody [SP2] - BSA and Azide free (ab239793)

This data was developed using **ab16661**, the same antibody clone in a different buffer formulation.

Multiplex immunohistochemistry (Formalin/PFA-fixed paraffin-embedded sections) analysis of Human triple-negative breast carcinoma tissue sections labeling Progesterone Receptor (PR) with **ab16661**, at a 1/6000 dilution (0.2 µg/ml). Heat mediated antigen retrieval with Citrate buffer (pH 6.0, epitope retrieval solution 1) for 20 mins and Opal Polymer HRP Ms + Rb was used as the secondary antibody. DAPI was used as the nuclear counterstain.

Panel A: merged staining of anti-Progesterone Receptor (PR) (magenta; Opal™690), anti-HER2 (red; Opal™570) and anti-Estrogen Receptor (ER) (green; Opal™520) on human triple-negative breast carcinoma.
Panel B: anti-PR stained on no cells.

Panel C: anti-HER2 stained on no cells.

Panel D: anti-ER stained on no cells.

The section was incubated in three rounds of staining: in the order of **ab16661** for 30 mins, then **ab16660** and **ab231438** for 10 mins at room temperature. Each round was followed by a separate fluorescent tyramide signal amplification system.

The immunostaining was performed on a Leica Biosystems BOND® RX instrument with an Opal™ 4-color kit. Image acquisition was performed with Leica SP8 confocal microscope.

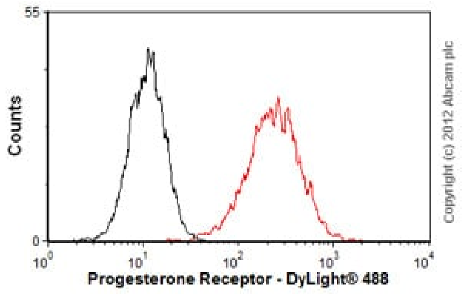

Flow Cytometry - Anti-Progesterone Receptor antibody [SP2] - BSA and Azide free (ab239793)

Overlay histogram showing T47D cells stained with **ab16661** (red line). The cells were fixed with 80% methanol (5 min) and then permeabilized with 0.1% PBS-Tween for 20 min. The cells were then incubated in 1x PBS / 10% normal goat serum / 0.3M glycine to block non-specific protein-protein interactions followed by the antibody (**ab16661**, 1/100 dilution) for 30 min at 22°C. The secondary antibody used was DyLight® 488 goat anti-rabbit IgG (H+L) (**ab96899**) at 1/500 dilution for 30 min at 22°C. Isotype control antibody (black line) was rabbit IgG (monoclonal) (1µg/1x10^6 cells) used under the same conditions. Acquisition of >5,000 events was performed.

This data was developed using the same antibody clone in a different buffer formulation containing PBS, BSA, glycerol, and sodium azide (**ab16661**).

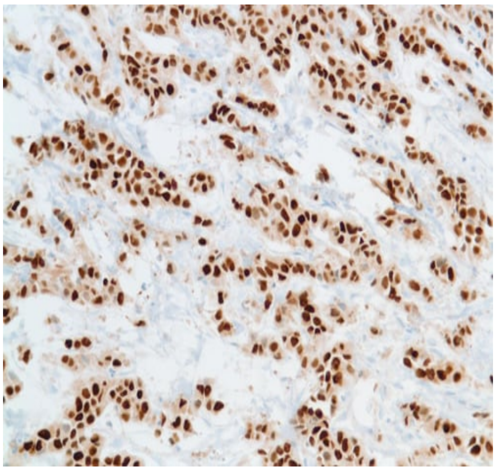

Immunohistochemistry (Formalin/PFA-fixed paraffin-embedded sections) - Anti-Progesterone Receptor antibody [SP2] - BSA and Azide free (ab239793)

Immunohistochemistry analysis of human breast carcinoma tissue labelling SP2 with **ab16661**.

This data was developed using the same antibody clone in a different buffer formulation containing Tris buffered saline, BSA, and sodium azide (**ab16661**).

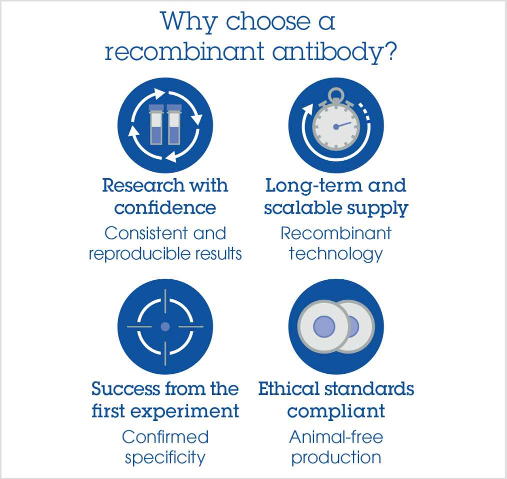

Anti-Progesterone Receptor antibody [SP2] - BSA and Azide free (ab239793)

Please note: All products are "FOR RESEARCH USE ONLY. NOT FOR USE IN DIAGNOSTIC PROCEDURES"

Our Abpromise to you: Quality guaranteed and expert technical support

- Replacement or refund for products not performing as stated on the datasheet
- Valid for 12 months from date of delivery
- Response to your inquiry within 24 hours

- We provide support in Chinese, English, French, German, Japanese and Spanish
- Extensive multi-media technical resources to help you
- We investigate all quality concerns to ensure our products perform to the highest standards

If the product does not perform as described on this datasheet, we will offer a refund or replacement. For full details of the Abpromise, please visit **https://www.abcam.com/abpromise** or contact our technical team.

Terms and conditions

- Guarantee only valid for products bought direct from Abcam or one of our authorized distributors

Anti-Human VEGF-163Dy

Pathologist-Verified Clone for Imaging Mass Cytometry™

Catalog: 3163028D
Package size and concentration: 25 µg, 0.5 mg/mL
Storage: Store at 4 °C. Do not freeze.
Reactivity: Human

Clone: G153-694
Isotype: Mouse IgG2b
Formulation: Antibody stabilizer with 0.05% sodium azide
Application: IMC-Paraffin

Technical Information

Application: The metal-tagged antibody is designed and formulated for the application of Imaging Mass Cytometry (IMC™) using the Fluidigm Hyperion™ Imaging System on formalin-fixed, paraffin-embedded (FFPE) tissue sections.

Quality control: Each lot of conjugated antibody is quality control-tested by Imaging Mass Cytometry on tissue sections.

Recommended concentration: For optimal performance it is recommended that the antibody be titrated for the desired application. Suggested initial dilution range:
IMC-Paraffin: 1:50 to 1:200

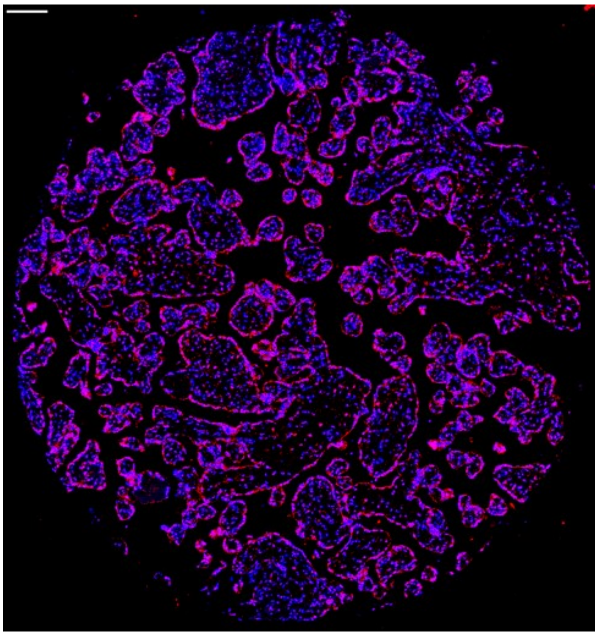

Human placenta (FFPE) stained with 163Dy-anti-VEGF (G153-694) at a dilution of 1:100 (red pseudocolor) and iridium DNA intercalator (blue pseudocolor).
Heat-mediated antigen retrieval was performed using Tris/EDTA buffer pH 9. Scale bar size = 100 µm.

Description

VEGF (vascular endothelial growth factor), also known as VEGFA, is a heparin-binding, dimeric glycosylated protein of 46–48 kDa that is related to the PDGF/VEGF family of growth factors. VEGF is secreted by pituitary cells, monocytes/macrophages, smooth muscle cells and keratinocytes. It is also secreted by tumor cells and other cells exposed to hypoxia. VEGF is a highly specific mitogen for vascular endothelial cells and induces angiogenesis and permeabilization of blood vessels.

References

Chang, Q. et al. "Staining of frozen and formalin-fixed, paraffin-embedded tissues with metal-labeled antibodies for imaging mass cytometry analysis." *Current Protocols in Cytometry* 82 (2017): 12.47.1–12.47.8.

Giesen, C. et al. "Highly multiplexed imaging of tumor tissues with subcellular resolution by mass cytometry." *Nature Methods* 11 (2014): 417–22.

For technical support visit http://techsupport.fluidigm.com. | For general support visit www.fluidigm.com/support.

For Research Use Only. Not for use in diagnostic procedures.

Information in this publication is subject to change without notice. **Safety data sheet information:** www.fluidigm.com/sds. **Patent and license information:** www.fluidigm.com/legalnotices. **Limited Use Label License:** The purchase of this product conveys to the purchaser the limited, non-transferable right to use the purchased consumable or reagent only with Fluidigm Instruments and Systems. **EU's WEEE directive information:** www.fluidigm.com/compliance. Fluidigm, the Fluidigm logo, Hyperion, Imaging Mass Cytometry, and IMC are trademarks and/or registered trademarks of Fluidigm Corporation in the United States and/or other countries. © 2018 Fluidigm Corporation. All rights reserved. 02/2018

Anti-β-Catenin-165Ho

Pathologist-Verified Clone for Imaging Mass Cytometry™

Catalog: 3165032D
Package size and concentration: 25 µg, 0.5 mg/mL
Storage: Store at 4 °C. Do not freeze.
Reactivity: Rat, Mouse, Human, Guinea Pig, Monkey

Clone: D13A1
Isotype: Rabbit IgG
Formulation: Antibody stabilizer with 0.05% sodium azide
Application: IMC-Paraffin

Technical Information

Application: The metal-tagged antibody is designed and formulated for the application of Imaging Mass Cytometry (IMC™) using the Fluidigm Hyperion™ Imaging System on formalin-fixed, paraffin-embedded (FFPE) tissue sections.

Quality control: Each lot of conjugated antibody is quality control-tested by Imaging Mass Cytometry on tissue sections.

Recommended concentration: For optimal performance it is recommended that the antibody be titrated for the desired application. Suggested initial dilution range:
IMC-Paraffin: 1:100 to 1:400

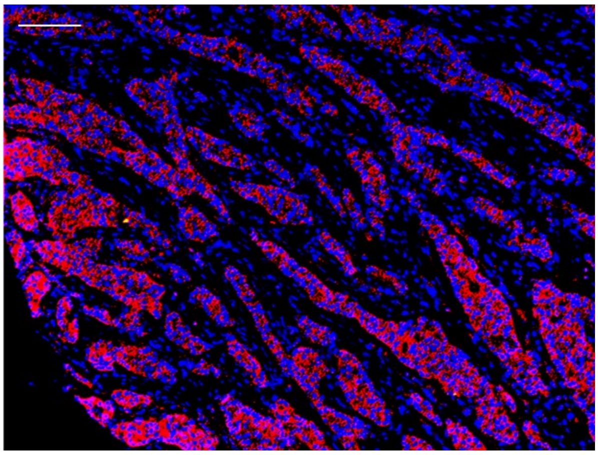

Human breast carcinoma (FFPE) stained with 165Ho-anti-β-catenin (D13A1) at a dilution of 1:200 (red pseudocolor) and iridium DNA intercalator (blue pseudocolor). Heat-mediated antigen retrieval was performed using Tris/EDTA buffer pH 9. Scale bar size = 100 µm.

Description

β-catenin is a 92 kDa intracellular protein that binds to the cytoplasmic tail of E-cadherin to mediate cellular adhesion. In addition, it is a key downstream effector in the Wnt signaling pathway. In the absence of Wnt binding its receptor, β-catenin is phosphorylated and resides in the cytoplasm, where it is eventually targeted for degradation by ubiquitination. Upon Wnt binding, β-catenin becomes dephosphorylated, translocates to the nucleus and modulates gene expression in partnership with the transcription factors T cell factor (TCF) and lymphocyte enhancer binding factor (LEF). Expression of β-catenin is found in a wide variety of nonimmune and immune tissues, including thymocytes and T and B lymphocytes. Clone D13A1 recognizes endogenous β-catenin protein only when residues Ser33, Ser37 and Thr41 are not phosphorylated.

References

Chang, Q. et al. "Staining of frozen and formalin-fixed, paraffin-embedded tissues with metal-labeled antibodies for imaging mass cytometry analysis." *Current Protocols in Cytometry* 82 (2017): 12.47.1–12.47.8.

Giesen, C. et al. "Highly multiplexed imaging of tumor tissues with subcellular resolution by mass cytometry." *Nature Methods* 11 (2014): 417–22.

For Research Use Only. Not for use in diagnostic procedures.

This product contains antibodies manufactured by and sold under license from CST™ and licensees thereof.
Information in this publication is subject to change without notice. **Safety data sheet information:** www.fluidigm.com/sds. **Patent and license information:** www.fluidigm.com/legalnotices. **Limited Use Label License:** The purchase of this product conveys to the purchaser the limited, non-transferable right to use the purchased consumable or reagent only with Fluidigm Instruments and Systems. **EU's WEEE directive information:** www.fluidigm.com/compliance. Fluidigm, the Fluidigm logo, Hyperion, Imaging Mass Cytometry, and IMC are trademarks or registered trademarks of Fluidigm Corporation in the United States and/or other countries. All other trademarks are the sole property of their respective owners. © 2017 Fluidigm Corporation. All rights reserved. 10/2017

Purified anti-Podoplanin (Lymphatic Endothelial Marker) Antibody

**Catalog# / Size**	916605 / 25 µg<br>916606 / 100 µg
**Clone**	D2-40
**Regulatory Status**	RUO
**Other Names**	Podoplanin, T1-alpha, hT1alpha-1, hT1alpha-2, PA2.26 antigen, glycoprotein 36, lung type-I cell membrane-associated glycoprotein (T1A-2)
**Isotype**	Mouse IgG1, κ
**Description**	Podoplanin is expressed by a variety of normal cells, which include breast and prostate myopepithelial cells, follicular dendritic cells, basal keratinocytes of the skin and cervix (focal), type I pneumocytes, ependymal cells, and fetal cerebral germinal matrix cells. Podoplanin is a marker of lymphatic differentiation because it is expressed by normal human lymphatic endothelium, but not by vascular endothelium. It is diagnostically a useful marker for the evaluation of a variety of neoplasms, including Kaposi sarcoma mesothelioma, testicular germ cell tumors, and cutanneous sebaceous neoplasms.

Product Details

**Verified Reactivity**	Human
**Antibody Type**	Monoclonal
**Host Species**	Mouse
**Immunogen**	M2A protein derived from human germ cell tumors.
**Formulation**	Phosphate-buffered solution, pH 7.2.
**Preparation**	The antibody was purified by affinity chromatography.
**Concentration**	1.0 mg/ml
**Storage & Handling**	The antibody solution should be stored undiluted between 2°C and 8°C.
**Application**	IHC-P - *Quality tested*<br>WB - *Verified*<br>ELISA Detection, ICC - *Reported in the literature, not verified in house*
**Recommended Usage**	Each lot of this antibody is quality control tested by immunohistochemistry. For immunohistochemistry on formalin-fixed paraffin-embedded tissue, a concentration range of 0.5 - 5.0 µg/ml is suggested. For Western blotting, the suggested use of this reagent is 2.0 - 5.0 µg per ml. It is recommended that the reagent be titrated for optimal performance for each application.
**Application Notes**	Additional reported applications (for the relevant formats) include: ELISA Detection[5] and immunocytochemistry[4].

Application References

(PubMed link indicates BioLegend citation)

1. Hamanaka T, *et al.* 2011. *Invest. Ophthalmol. Vis. Sci.* 52:8849. (IHC-P)
2. Choi WW, *et al.* 2005. *Mod. Pathol.* 18:143. (IHC-P)
3. Chu AY, *et al.* 2005. *Mod. Pathol.* 18:105. (IHC-P) PubMed
4. Yoon C, *et al.* 2008. *Blood* 112:1129. (ICC) PubMed
5. Schacht V, *et al.* 2005. *American Journal of Pathology* 166:913. (ELISA Detection, WB) PubMed
6. Marks A, *et al.* 1999. *British Journal of Cancer* 80:569.

Product Citations

1. Burlingame EA, *et al.* 2021. Cell Rep Methods. 1:. PubMed
2. Yoon C, *et al.* 2008. Blood. 112:1129-1138. PubMed
3. Francescone R, *et al.* 2020. Cancer Discovery. 11(2):446-479. PubMed
4. Schacht V, *et al.* 2005. Am J Pathol. 166:913-921. PubMed
5. Chu A, *et al.* 2005. Mod Pathol. 18:105-110. PubMed

**RRID**	AB_2565819 (BioLegend Cat. No. 916605)<br>AB_2565820 (BioLegend Cat. No. 916606)

Antigen Details

**Structure**	35 kD.
**Distribution**	Membrane and plasma membrane.
**Function**	May be involved in cell migration and/or actin cytoskeleton organization. When expressed in keratinocytes, induces changes in cell morphology with transfected cells showing an elongated shape, numerous membrane protusions, major reorganization of the actin cytoskeleton, increased motility and decreased cell adhesion. Required for normal lung cell proliferation and alveolus formation at birth. Induces platelet aggregation. Does not have any effect on folic acid or amino acid transport and does not function as a water channel or as a regulator of aquaporin-type water channels.
**Interaction**	Cytoskeletal signaling.
**Biology Area**	Cancer Biomarkers, Cell Biology, Neuroinflammation, Neuroscience, Neuroscience Cell Markers
**Gene ID**	10630

Related Protocols

Active Protocols: Sandwich ELISA - Video

Western Blotting Protocol

Immunohistochemistry Protocol for Paraffin-Embedded Sections

Other Formats

D2-40 Lymphatic Endothelial Marker Monoclonal Antibody, Purified anti-Podoplanin (Lymphatic Endothelial Marker), Alexa Fluor® 594 anti-Podoplanin/Lymphatic Endothelial Marker, Alexa Fluor® 647 anti-Podoplanin (Lymphatic Endothelial Marker)

Product Data

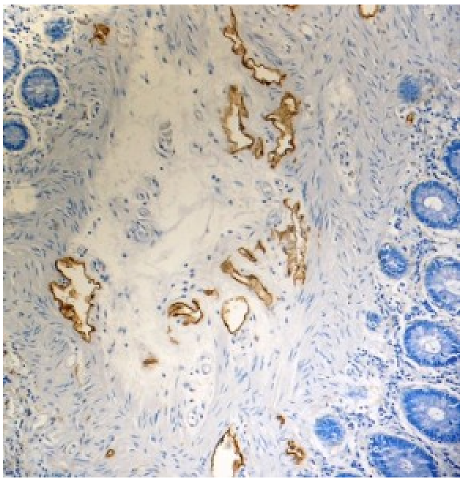

IHC staining of purified anti-Podoplanin (Lymphatic Endothelial Marker) antibody (clone D2-40) on formalin-fixed paraffin-embedded human colon tissue. The tissue was incubated with 1.3 µg/ml of the primary antibody at room temperature for 60 minutes. BioLegend's Ultra-Streptavidin (USA) HRP kit (Cat. No. 929901) was used for detection followed by hematoxylin counterstaining, according to the protocol provided. The image was captured with a 40X objective.

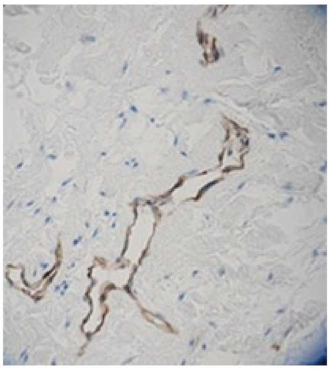

IHC staining of purified anti-Podoplanin (Lymphatic Endothelial Marker)antibody (clone D2-40) on formalin-fixed paraffin-embedded human colon tissue. The tissue was incubated with 1 µg/ml of the primary antibody for 60 minutes at room temperature. BioLegend´s Ultra-Streptavidin (USA) HRP kit (Multi-Species, DAB, Cat. No. 929901) was used for detection followed by hematoxylin counterstaining, according to the protocol provided. The image was captured with a 40X objective.

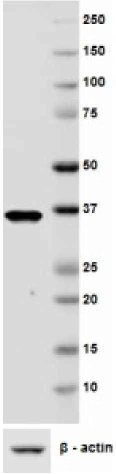

Western blot analysis of cell lysates from HeLa using Podoplanin Mouse primary antibody and HRP Goat anti-Mouse secondary antibody (Cat. No. 405306). Direct-Blot™ HRP anti-β-actin (Cat. No. 643807) was used as a loading control.

For research use only. Not for diagnostic use. Not for resale. BioLegend will not be held responsible for patent infringement or other violations that may occur with the use of our products.

*These products may be covered by one or more Limited Use Label Licenses (see the BioLegend Catalog or our website, www.biolegend.com/ordering#license). BioLegend products may not be transferred to third parties, resold, modified for resale, or used to manufacture commercial products, reverse engineer functionally similar materials, or to provide a service to third parties without written approval of BioLegend. By use of these products you accept the terms and conditions of all applicable Limited Use Label Licenses. Unless otherwise indicated, these products are for research use only and are not intended for human or animal diagnostic, therapeutic or commercial use.

8999 BioLegend Way, San Diego, CA 92121 www.biolegend.com
Toll-Free Phone: 1-877-Bio-Legend (246-5343) Phone: (858) 768-5800 Fax: (877) 455-9587

Anti-Ki-67 (B56)-168Er

Pathologist-Verified Clone for Imaging Mass Cytometry™

Catalog number: 3168022D
Package size and concentration: 25 µg, 0.5 mg/mL
Clone: B56
Isotype: Mouse IgG1
Pathologist-verified on: Human FFPE, Human Frozen
Fluidigm tested on: Human FFPE, Human-Frozen, Mouse FFPE

Reported reactivity: Human, Mouse, Rat, Porcine, Cross
Formulation: Antibody stabilizer with 0.05% sodium azide
Storage: Store at 4 °C. Do not freeze.
Application: IMC paraffin, IMC frozen

Technical Information

Description: Ki-67 protein, also known as MKI67, is a nuclear protein that is associated with cellular proliferation. Ki-67 protein is expressed in all cell types. It is present during active phases of the cell cycle (G1, S, G2, and mitosis) and not during the resting phase, G0. Because of this, Ki-67 is an excellent marker for determining the fraction of proliferating cells within a given population of cells.

Application: The metal-tagged antibody is designed and formulated for the application of Imaging Mass Cytometry™ (IMC™) using the Fluidigm Hyperion™ Imaging System on formalin-fixed, paraffin-embedded (FFPE) tissue sections, and frozen tissue sections.

Quality control: Each lot of conjugated antibody is quality control- tested by Imaging Mass Cytometry on tissue sections

Recommended concentration: For optimal performance it is recommended that the antibody be titrated for the desired application. Suggested initial dilution range:
IMC-Paraffin: 1:25 to 1:100
IMC-Frozen: 1:50 to 1:200

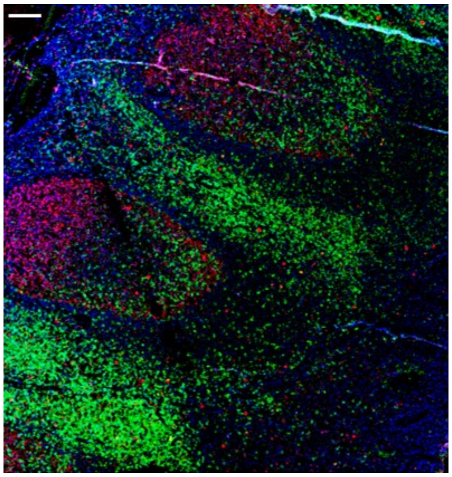

Human tonsil (FFPE) stained with 168Er-anti-Ki-67 (B56) at a dilution of 1:50 (red pseudocolor), 170Er-anti-CD3 (poly) (green pseudocolor), and iridium DNA intercalator (blue pseudocolor). Heat-mediated antigen retrieval was performed using Tris/EDTA buffer pH 9.
Scale bar size = 100 µm.

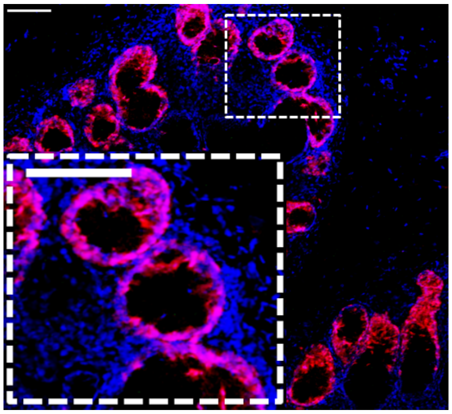

Human frozen colon stained with 168Er-anti-Ki-67 at a dilution of 1:100 (B56) (red pseudocolor) and iridium DNA intercalator (blue pseudocolor). Tissue section was fixed in 4% paraformaldehyde for 30 minutes at 4 °C.
Scale bar size = 100 µm.

References

Chang, Q. et al. "Staining of frozen and formalin-fixed, paraffin-embedded tissues with metal-labeled antibodies for Imaging Mass Cytometry analysis." Current Protocols in Cytometry 82 (2017): 12.47.1–12.47.8.

Giesen, C. et al. "Highly multiplexed imaging of tumor tissues with subcellular resolution by mass cytometry." Nature Methods 11 (2014): 417–22.

Safety

Use standard laboratory safety protocols. Read and understand the safety data sheets (SDSs) before handling chemicals. To obtain SDSs, go to fluidigm.com/sds and search for the SDS using either the product name or the part number.

For Research Use Only. Not for use in diagnostic procedures.

Information in this publication is subject to change without notice. **Limited Use Label License:** The purchase of this Fluidigm Instrument and/or Consumable product conveys to the purchaser the limited, nontransferable right to use with only Fluidigm Consumables and/or Instruments respectively except as approved in writing by Fluidigm. **Patent and license information:** fluidigm.com/legal/notices. **Trademarks:** Fluidigm, the Fluidigm logo, Hyperion, Imaging Mass Cytometry, and IMC are trademarks and/or registered trademarks of Fluidigm Corporation in the United States and/or other countries. All other trademarks are the sole property of their respective owners. © 2020 Fluidigm Corporation. All rights reserved. May 2020

Anti-Collagen Type I (Polyclonal)-169Tm

Pathologist-Verified Clone for Imaging Mass Cytometry™

Catalog number: 3169023D
Package size and concentration: 25 μg, 0.5 mg/mL
Clone: Poly
Isotype: Goat polyclonal
Pathologist-verified on: Human FFPE, Human Frozen
Fluidigm tested on: Human FFPE, Human Frozen, Mouse FFPE

Reported reactivity: Human, Mouse
Formulation: Antibody stabilizer with 0.05% sodium azide
Storage: Store at 4 °C. Do not freeze.
Application: IMC paraffin, IMC frozen

Technical Information

Description: Collagen type I is a fibrillar-forming, extracellular matrix protein composed of 2 alpha 1 chains and 1 alpha 2 chain in a triple helix. It is the predominant form of collagen in the human body. It forms the fibrils of tendon, ligaments, and bones, and it plays a role in scar tissue formation. Mutations in this gene are associated with osteogenesis imperfecta types I–IV, Ehlers-Danlos syndrome type VIIA, Ehlers-Danlos syndrome classical type, Caffey disease and idiopathic osteoporosis.

Application: The metal-tagged antibody is designed and formulated for the application of Imaging Mass Cytometry™ (IMC™) using the Fluidigm Hyperion™ Imaging System on formalin-fixed, paraffin-embedded (FFPE) tissue sections, and frozen tissue sections.

Quality control: Each lot of conjugated antibody is quality control- tested by Imaging Mass Cytometry on tissue sections

Recommended concentration: For optimal performance it is recommended that the antibody be titrated for the desired application. Suggested initial dilution range:

IMC-Paraffin: 1:150 to 1:600

IMC-Frozen: 1:200 to 1:800

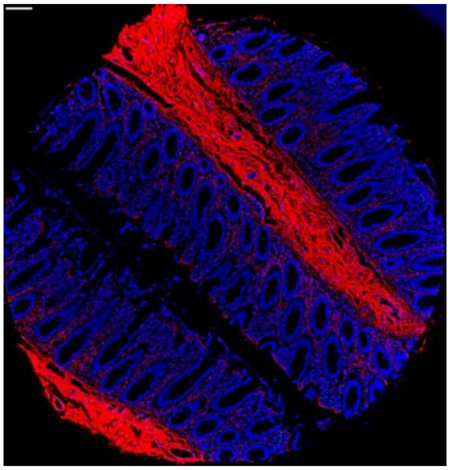

Human normal colon (FFPE) stained with 169Tm-anti-collagen I (poly) at a dilution of 1:300 (red pseudocolor) and iridium DNA intercalator (blue pseudocolor). Heat-mediated antigen retrieval was performed using Tris/EDTA buffer pH 9.

Scale bar size = 100 μm.

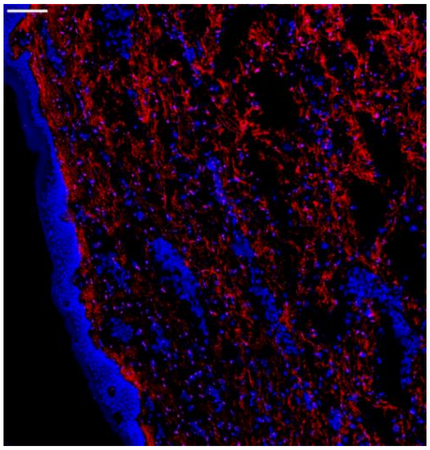

Human frozen colon stained with 169Tm-anti-collagen I (poly) at a dilution of 1:500 (red pseudocolor) and iridium DNA intercalator (blue pseudocolor). Tissue section was fixed in 4% paraformaldehyde for 30 minutes at 4 °C.

Scale bar size = 100 μm.

References

Chang, Q. et al. "Staining of frozen and formalin-fixed, paraffin-embedded tissues with metal-labeled antibodies for Imaging Mass Cytometry analysis." Current Protocols in Cytometry 82 (2017): 12.47.1–12.47.8.

Giesen, C. et al. "Highly multiplexed imaging of tumor tissues with subcellular resolution by mass cytometry." Nature Methods 11 (2014): 417–22.

Safety

Use standard laboratory safety protocols. Read and understand the safety data sheets (SDSs) before handling chemicals. To obtain SDSs, go to fluidigm.com/sds and search for the SDS using either the product name or the part number.

For Research Use Only. Not for use in diagnostic procedures.

Information in this publication is subject to change without notice. **Limited Use Label License:** The purchase of this Fluidigm Instrument and/or Consumable product conveys to the purchaser the limited, nontransferable right to use with only Fluidigm Consumables and/or Instruments respectively except as approved in writing by Fluidigm. **Patent and license information:** fluidigm.com/legal/notices. **Trademarks:** Fluidigm, the Fluidigm logo, Hyperion, Imaging Mass Cytometry, and IMC are trademarks and/or registered trademarks of Fluidigm Corporation in the United States and/or other countries. All other trademarks are the sole property of their respective owners. © 2020 Fluidigm Corporation. All rights reserved. 24-Apr-2020

Anti-CD3 (Polyclonal, C-Terminal)-170Er

Pathologist-Verified Clone for Imaging Mass Cytometry™

Catalog number: 3170019D
Package size and concentration: 25 µg, 0.5 mg/mL
Clone: Poly C-Terminal
Host Species: Rabbit
Pathologist-verified on: Human FFPE
Fluidigm tested on: Human FFPE, Mouse FFPE

Reported reactivity: Human, Mouse, Chimpanzee
Formulation: Antibody stabilizer with 0.05% sodium azide
Storage: Store at 4 °C. Do not freeze.
Application: IMC paraffin

Technical Information

Description: CD3 is a transmembrane subunit of the T cell receptor (TCR) complex. It is a member of the immunoglobulin superfamily, and it plays a role in antigen recognition, signal transduction, and T cell activation. CD3 is expressed by T lymphocytes (thymocytes) at the highest levels on mature cell types.

Application: The metal-tagged antibody is designed and formulated for the application of Imaging Mass Cytometry™ (IMC™) using the Fluidigm Hyperion™ Imaging System on formalin-fixed, paraffin-embedded (FFPE) tissue sections.

Quality control: Each lot of conjugated antibody is quality control- tested by Imaging Mass Cytometry on tissue sections

Recommended concentration: For optimal performance it is recommended that the antibody be titrated for the desired application. Suggested initial dilution range:
IMC-Paraffin: 1:50 to 1:200

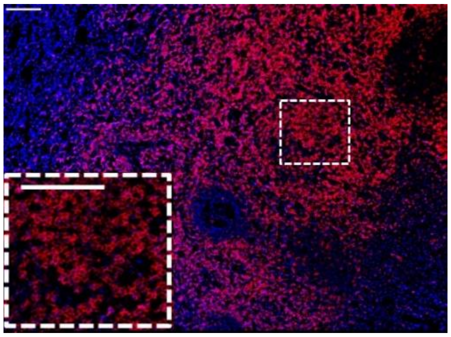

Human tonsil (FFPE) stained with 170Er-anti-CD3 (poly) at a dilution of 1:100 (red pseudocolor) and iridium DNA intercalator (blue pseudocolor). Heat-mediated antigen retrieval was performed using Tris/EDTA buffer pH 9.
Scale bar size = 100 µm.

References

Chang, Q. et al. "Staining of frozen and formalin-fixed, paraffin-embedded tissues with metal-labeled antibodies for Imaging Mass Cytometry analysis." Current Protocols in Cytometry 82 (2017): 12.47.1–12.47.8.

Giesen, C. et al. "Highly multiplexed imaging of tumor tissues with subcellular resolution by mass cytometry." Nature Methods 11 (2014): 417–22.

Safety

Use standard laboratory safety protocols. Read and understand the safety data sheets (SDSs) before handling chemicals. To obtain SDSs, go to fluidigm.com/sds and search for the SDS using either the product name or the part number.

For Research Use Only. Not for use in diagnostic procedures.

Information in this publication is subject to change without notice. **Limited Use Label License:** The purchase of this Fluidigm Instrument and/or Consumable product conveys to the purchaser the limited, nontransferable right to use with only Fluidigm Consumables and/or Instruments respectively except as approved in writing by Fluidigm. **Patent and license information:** fluidigm.com/legal/notices. **Trademarks:** Fluidigm, the Fluidigm logo, Hyperion, Imaging Mass Cytometry, and IMC are trademarks and/or registered trademarks of Fluidigm Corporation in the United States and/or other countries. All other trademarks are the sole property of their respective owners. © 2020 Fluidigm Corporation. All rights reserved. April 2021

Anti-pERK1/2 [T202/Y204] (D13.14.4E)-171Yb

Pathologist-Verified Clone for Imaging Mass Cytometry™

Catalog number: 3171021D
Package size and concentration: 25 µg, 0.5 mg/mL
Clone: D13.14.4E
Isotype: Rabbit IgG
Pathologist-verified on: Human FFPE
Fluidigm tested on: Human FFPE, Mouse FFPE

Reported reactivity: Human, Mouse, Rat, Bovine, Canine, Porcine, Hamster, Monkey
Formulation: Antibody stabilizer with 0.05% sodium azide
Storage: Store at 4 °C. Do not freeze.
Application: IMC paraffin

Technical Information

Description: ERK1 and ERK2, also known as p44 and p42 MAPKs, are similar (85% sequence identity) members of the mitogen-activated protein kinase (MAPK) family of serine/threonine protein kinases. ERK1/2 signaling is important in the cellular response to a wide range of stimuli including growth factors, cytokines, and mitogens. The signal cascade upstream of ERK1/2 typically begins with receptor tyrosine kinases phosphorylating members of the Raf family and other MAP kinase kinase kinases (MAP3Ks), which thereby activate MEK1 and MEK2, the MAP kinase kinases (MAPKKs) directly responsible for phosphorylation of ERK1 and ERK2. ERK1 and ERK2 are activated through phosphorylation of the activation loop residues Thr202/Tyr204 and Thr185/Tyr187, and dual phosphorylation is required for full activity. ERK1/2 can activate the RSK family of kinases in the cytoplasm and transcription factors including Elk-1 in the nucleus.

Application: The metal-tagged antibody is designed and formulated for the application of Imaging Mass Cytometry™ (IMC™) using the Fluidigm Hyperion™ Imaging System on formalin-fixed, paraffin-embedded (FFPE) tissue sections.

Quality control: Each lot of conjugated antibody is quality control- tested by Imaging Mass Cytometry on tissue sections

Recommended concentration: For optimal performance it is recommended that the antibody be titrated for the desired application. Suggested initial dilution range:
IMC-Paraffin: 1:25 to 1:100

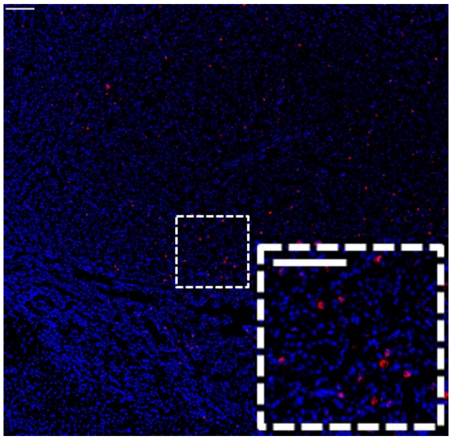

Human hepatocellular carcinoma (FFPE) stained with 171Yb-anti-pERK1/2 (D13.14.4E) at a dilution of 1:50 (red pseudocolor) and iridium DNA intercalator (blue pseudocolor). Heat-mediated antigen retrieval was performed using Tris/EDTA buffer pH 9.
Scale bar size = 100 µm.

References

Chang, Q. et al. "Staining of frozen and formalin-fixed, paraffin-embedded tissues with metal-labeled antibodies for Imaging Mass Cytometry analysis." Current Protocols in Cytometry 82 (2017): 12.47.1–12.47.8.

Giesen, C. et al. "Highly multiplexed imaging of tumor tissues with subcellular resolution by mass cytometry." Nature Methods 11 (2014): 417–22.

Safety

Use standard laboratory safety protocols. Read and understand the safety data sheets (SDSs) before handling chemicals. To obtain SDSs, go to fluidigm.com/sds and search for the SDS using either the product name or the part number.

For Research Use Only. Not for use in diagnostic procedures.

This product contains antibodies manufactured by and sold under license from CST™ and licensees thereof.
Information in this publication is subject to change without notice. **Limited Use Label License:** The purchase of this Fluidigm Instrument and/or Consumable product conveys to the purchaser the limited, nontransferable right to use with only Fluidigm Consumables and/or Instruments respectively except as approved in writing by Fluidigm. **Patent and license information:** fluidigm.com/legal/notices. **Trademarks:** Fluidigm, the Fluidigm logo, Hyperion, Imaging Mass Cytometry, and IMC are trademarks and/or registered trademarks of Fluidigm Corporation in the United States and/or other countries. All other trademarks are the sole property of their respective owners. © 2020 Fluidigm Corporation. All rights reserved. 04-2020

Anti-pS6 [S235/S236] (N7-548)-175Lu

Pathologist-Verified Clone for Imaging Mass Cytometry™

Catalog number: 3175031D
Package size and concentration: 25 µg, 0.5 mg/mL
Clone: N7-548
Isotype: Mouse IgG1
Pathologist-verified on: Human FFPE
Fluidigm tested on: Human FFPE, Mouse FFPE

Reported reactivity: Human, Mouse, Rat
Formulation: Antibody stabilizer with 0.05% sodium azide
Storage: Store at 4 °C. Do not freeze.
Application: IMC paraffin

Technical Information

Description: Ribosomal protein S6 belongs to the S6E family of ribosomal proteins and is a component of the 40S ribosomal subunit. It plays a role in regulation of translation and thus relates to the growth of cells. Phosphorylation of S6 at multiple C-terminal serine residues, including S235, S236, S240, and S244, activates it. Activated S6 up-regulates ribosomal translation of RNA species. These phosphorylations are mediated by various kinases, including p70S6K and PKCδ, and activated through cellular responses to extrinsic factors. The N7-548 monoclonal antibody specifically detects the S6 ribosomal protein phosphorylated at S235 and S236.

Application: The metal-tagged antibody is designed and formulated for the application of Imaging Mass Cytometry™ (IMC™) using the Fluidigm Hyperion™ Imaging System on formalin-fixed, paraffin-embedded (FFPE) tissue sections.

Quality control: Each lot of conjugated antibody is quality control- tested by Imaging Mass Cytometry on tissue sections

Recommended concentration: For optimal performance it is recommended that the antibody be titrated for the desired application. Suggested initial dilution range:
IMC-Paraffin: 1:50 to 1:200

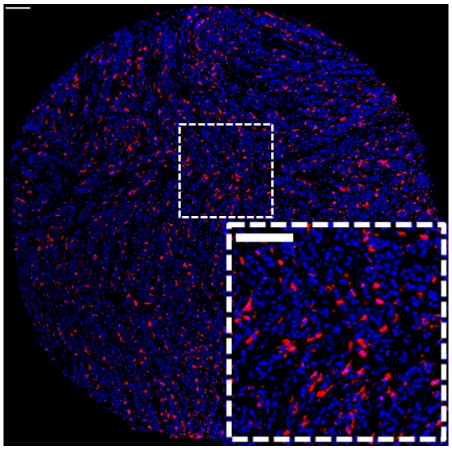

Human breast carcinoma (FFPE) stained with 175Lu-anti-pS6 (N7-548) at a dilution of 1:100 (red pseudocolor) and iridium DNA intercalator (blue pseudocolor). Heat-mediated antigen retrieval was performed using Tris/EDTA buffer pH 9.
Scale bar size = 100 µm.

References

Chang, Q. et al. "Staining of frozen and formalin-fixed, paraffin-embedded tissues with metal-labeled antibodies for Imaging Mass Cytometry analysis." Current Protocols in Cytometry 82 (2017): 12.47.1–12.47.8.

Giesen, C. et al. "Highly multiplexed imaging of tumor tissues with subcellular resolution by mass cytometry." Nature Methods 11 (2014): 417–22.

Safety

Use standard laboratory safety protocols. Read and understand the safety data sheets (SDSs) before handling chemicals. To obtain SDSs, go to fluidigm.com/sds and search for the SDS using either the product name or the part number.

For Research Use Only. Not for use in diagnostic procedures.

Information in this publication is subject to change without notice. **Limited Use Label License:** The purchase of this Fluidigm Instrument and/or Consumable product conveys to the purchaser the limited, nontransferable right to use with only Fluidigm Consumables and/or Instruments respectively except as approved in writing by Fluidigm. **Patent and license information:** fluidigm.com/legal/notices. **Trademarks:** Fluidigm, the Fluidigm logo, Hyperion, Imaging Mass Cytometry, and IMC are trademarks and/or registered trademarks of Fluidigm Corporation in the United States and/or other countries. All other trademarks are the sole property of their respective owners. © 2020 Fluidigm Corporation. All rights reserved. 04-2020

Anti-Histone 3 (D1H2)-176Yb

Pathologist-Verified Clone for Imaging Mass Cytometry™

Catalog number: 3176023D
Package size and concentration: 25 µg, 0.5 mg/mL
Clone: D1H2
Isotype: Rabbit IgG
Pathologist-verified on: Human FFPE
Fluidigm tested on: Human FFPE, Mouse FFPE

Reported reactivity: Human, Mouse, Rat, Bovine, Hamster, Monkey
Formulation: Antibody stabilizer with 0.05% sodium azide
Storage: Store at 4 °C. Do not freeze.
Application: IMC paraffin

Technical Information

Description: Histone 3 is a 17 kDa nuclear protein that is a component of an octamer containing pairs of each of 4 core histones (H2A, H2B, H3, H4). Histone 3, featuring a main globular domain and a long N-terminal tail, is involved with nucleosome structure of chromosomal fiber in eukaryotes. Histone 3 can be modified by phosphorylation, acetylation, ubiquitination, ribosylation and methylation. The N-terminal tail of histone 3 protrudes from the globular nucleosome core and can undergo several different types of post-translational modification that influence cellular processes.

Application: The metal-tagged antibody is designed and formulated for the application of Imaging Mass Cytometry™ (IMC™) using the Fluidigm Hyperion™ Imaging System on formalin-fixed, paraffin-embedded (FFPE) tissue sections.

Quality control: Each lot of conjugated antibody is quality control- tested by Imaging Mass Cytometry on tissue sections

Recommended concentration: For optimal performance it is recommended that the antibody be titrated for the desired application. Suggested initial dilution range:
IMC-Paraffin: 1:50 to 1:600

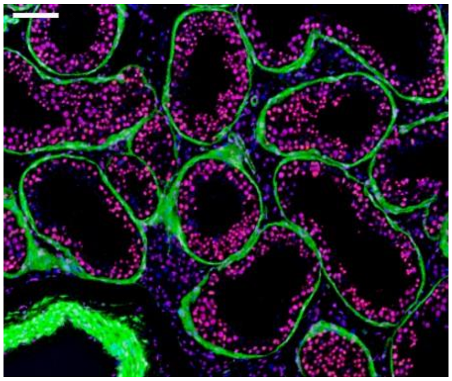

Human testis (FFPE) stained with 176Yb-anti-histone 3 (D1H2) at a dilution of 1:300 (red pseudocolor), 141Pr-anti-aSMA (1A4) (green pseudocolor), and iridium DNA intercalator (blue pseudocolor). Heat-mediated antigen retrieval was performed using Tris/EDTA buffer pH 9.
Scale bar size = 100 µm.

References

Chang, Q. et al. "Staining of frozen and formalin-fixed, paraffin-embedded tissues with metal-labeled antibodies for Imaging Mass Cytometry analysis." Current Protocols in Cytometry 82 (2017): 12.47.1–12.47.8.

Giesen, C. et al. "Highly multiplexed imaging of tumor tissues with subcellular resolution by mass cytometry." Nature Methods 11 (2014): 417–22.

Safety

Use standard laboratory safety protocols. Read and understand the safety data sheets (SDSs) before handling chemicals. To obtain SDSs, go to fluidigm.com/sds and search for the SDS using either the product name or the part number.

For technical support visit techsupport.fluidigm.com. | For general support visit fluidigm.com/support.

For Research Use Only. Not for use in diagnostic procedures.

This product contains antibodies manufactured by and sold under license from CST™ and licensees thereof.

Information in this publication is subject to change without notice. **Limited Use Label License:** The purchase of this Fluidigm Instrument and/or Consumable product conveys to the purchaser the limited, nontransferable right to use with only Fluidigm Consumables and/or Instruments respectively except as approved in writing by Fluidigm. **Patent and license information:** fluidigm.com/legal/notices. **Trademarks:** Fluidigm, the Fluidigm logo, Hyperion, Imaging Mass Cytometry, and IMC are trademarks and/or registered trademarks of Fluidigm Corporation in the United States and/or other countries. All other trademarks are the sole property of their respective owners. © 2020 Fluidigm Corporation. All rights reserved. 24-Apr-2020

Cell-ID Intercalator-Ir

**Catalog number, concentration:**	201192A, 125 µM<br>201192B, 500 µM
**Package size:**	500 µL

Storage: Upon receiving this product, aliquot and freeze at –20 °C. Frozen aliquots should be used only once after thawing.

Application: Cell identification on CyTOF® suspension mass cytometry systems

Technical Information

Description: Cell-ID™ Intercalator-Ir is a cationic nucleic acid intercalator that contains natural abundance iridium (191Ir and 193Ir) and is used to identify nucleated cells in CyTOF system analysis. When cells are stained with Intercalator-Ir, it binds to cellular nucleic acid, and detection of both stable isotopes enables identification of nucleated cells. It is a live-cell membrane-impermeable dye and therefore requires cells to be fixed and/or permeabilized before staining.

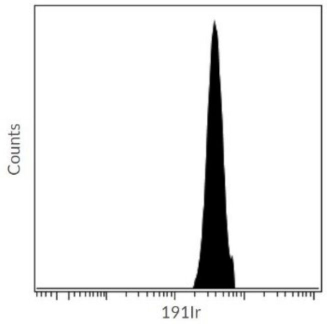

Human PBMC were stained with Cell-ID Intercalator-Ir. Cells shown are gated on total viable cells.

Important Product Notes

- Cell-ID Intercalator-Ir is a highly concentrated metal intercalator solution. It must be diluted in accordance with the applicable protocols to avoid early failure of the detector.
- Upon receiving this product, divide into single-use aliquots and freeze them at –20 °C. Frozen aliquots of Cell-ID Intercalator-Ir should be used only once immediately after thawing to room temperature. Avoid multiple freeze/thaw cycles as this may alter the chemical and cell-binding properties of the reagent.
- We recommend that you determine the optimal staining concentration for Cell-ID Intercalator-Ir by titrating the reagent for individual cell types and experiments. We suggest that the intercalator concentration in the staining solution not exceed 1 µM and optimal signal intensity is between 300 and 1,000 dual counts in the 191Ir channel.
- To minimize signal overlap due to the mass channel proximity, we recommend titrating Cell-ID Intercalator-Ir prior to use with platinum (194Pt)-labeled antibodies or Cell-ID Cisplatin-194Pt as a viability reagent.
- Detect the Cell-ID Intercalator-Ir metal isotope in the 191Ir or 193Ir mass channel of the CyTOF suspension mass cytometry system you will use for sample acquisition. Add the Cell-ID Intercalator-Ir metal isotopes to your acquisition template (.tem) prior to acquisition of samples. Refer to your CyTOF system user guide for information on how to add elements to the acquisition template and run samples using CyTOF Software.

Applicable Protocols

Before using this product, refer to any of the following documents for more information:

- Maxpar® Cell Surface Staining with Fresh Fix Protocol (400276)
- Maxpar Cytoplasmic/Secreted Antigen Staining with Fresh Fix Protocol (400279)
- Maxpar Nuclear Antigen Staining with Fresh Fix Protocol (400277)
- Maxpar Phosphoprotein Staining with Fresh Fix Protocol (400278)
- Maxpar Direct Immune Profiling Assay Cell Staining and Data Acquisition User Guide (400286)
- Cell-ID 20-Plex Pd Barcoding Kit User Guide (PRD023)

Safety

Use standard laboratory safety protocols. Read and understand the safety data sheets (SDSs) before handling chemicals. To obtain SDSs, go to fluidigm.com/sds and search for the SDS using either the product name or the part number.

For Research Use Only. Not for use in diagnostic procedures.

Information in this publication is subject to change without notice. **Patent and license information:** fluidigm.com/legal/notices. **Limited Use Label License:** The purchase of this Fluidigm Instrument and/or Consumable product conveys to the purchaser the limited, nontransferable right to use with only Fluidigm Consumables and/or Instruments respectively except as approved in writing by Fluidigm. **Trademarks:** Fluidigm, the Fluidigm logo, Cell-ID, CyTOF, and Maxpar are trademarks and/or registered trademarks of Fluidigm Corporation in the United States and/or other countries. © 2021 Fluidigm Corporation. All rights reserved. 04/2021
